# Supplementary material for: A multi-modal foundation model for brain disease diagnosis and medical imaging
Source: Patterns (N Y). 2026 Apr 14;7(6):101538. doi: 10.1016/j.patter.2026.101538 (PMC13280722; doi:10.1016/j.patter.2026.101538)
Supplement: Document S1. Figures S1–S38 and Table S1 [file mmc1.pdf]

Patterns, Volume *7*

Supplemental information

A multi-modal foundation model

for brain disease diagnosis and medical imaging

Guoxun Zhang, Zebin Gao, Caohui Duan, Jiaxin Liu, Yuerong Lizhu, Yaou Liu, Qian Chen, Ling Wang, Kailun Fei, Tianyun Wang, YuJia Chen, Yanchen Guo, Feng Xu, Yuchen Guo, Xin Lou, and Qionghai Dai

A multi-modal foundation model for brain disease diagnosis and medical imaging

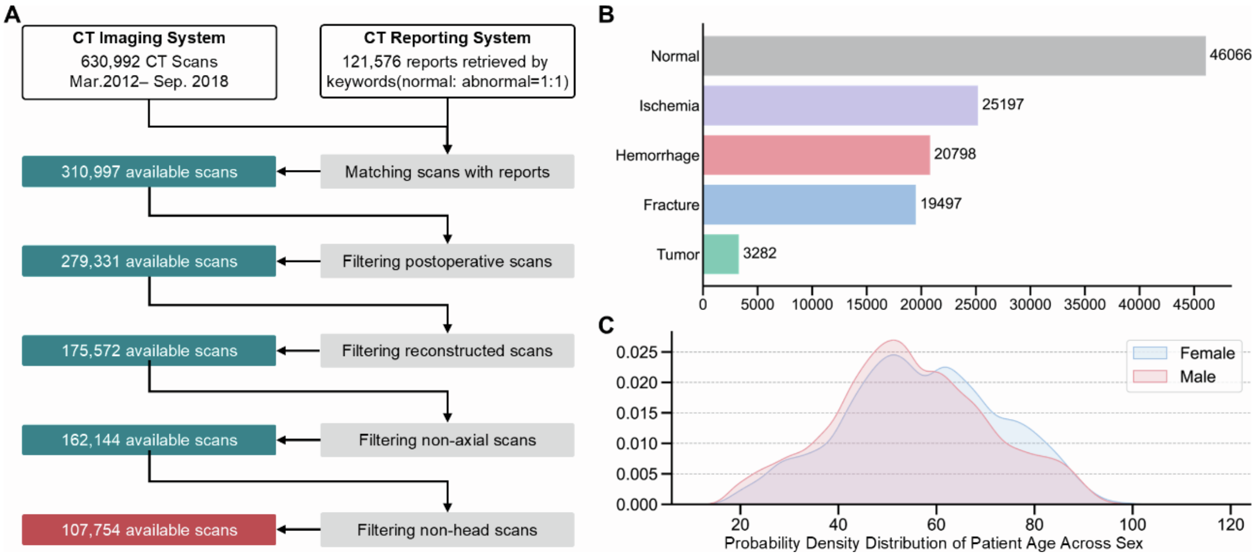

Fig. S1. Dataset overview of BrainCT-3M, used for pre-training Brainfound

(A) Data collection and preprocessing. A total of 630,992 brain CT scans and 121,576 associated diagnostic reports were collected as the source database. The data were subsequently screened using image quality criteria and a natural language processing (NLP) based report filtering procedure (Methods), culminating in the curated pre-training dataset BrainCT-3M. BrainCT-3M comprises 107,754 brain CT scans with matched reports, totaling over 3 million images.

(B) Distribution of CT studies across examination types in BrainCT-3M.

(C) Patient age distribution in BrainCT-3M.

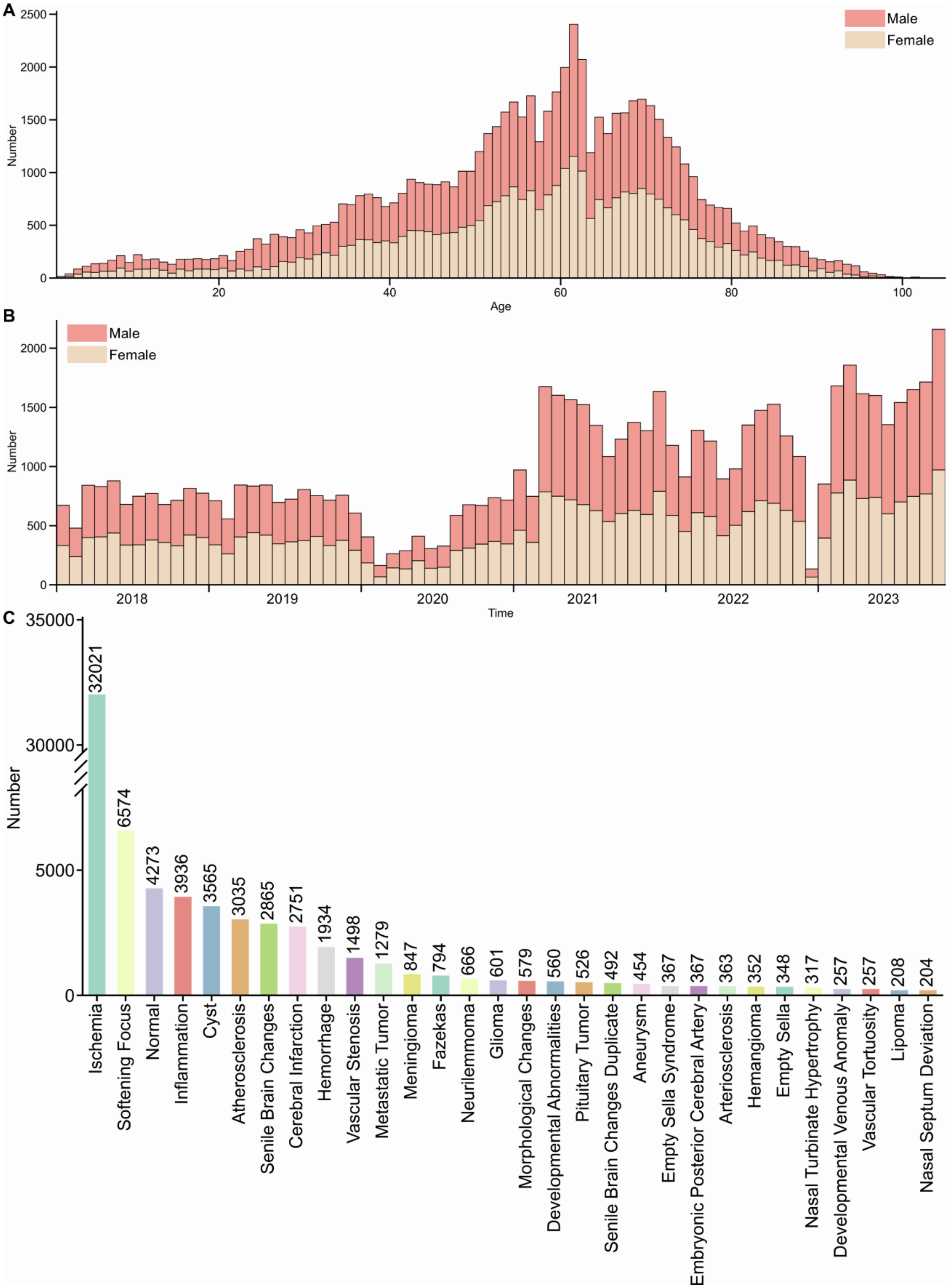

13 **Fig. S2. Dataset overview of BrainMRI-7M, used for pre-training Brainfound**

(A) Patient age distribution in BrainMRI-7M. BrainMRI-7M consists of brain MRI studies from 68,653 patients, totaling approximately 7 million images.

(B) Distribution of examination dates in BrainMRI-7M. The dataset spans a five-year period, from 2018 to 2023.

(C) Token and term-frequency statistics for the associated BrainMRI-7M reports. Report text was tokenized using an automated NLP pipeline. Among 68,653 MRI reports, the top 30 most frequent clinical terms are shown.

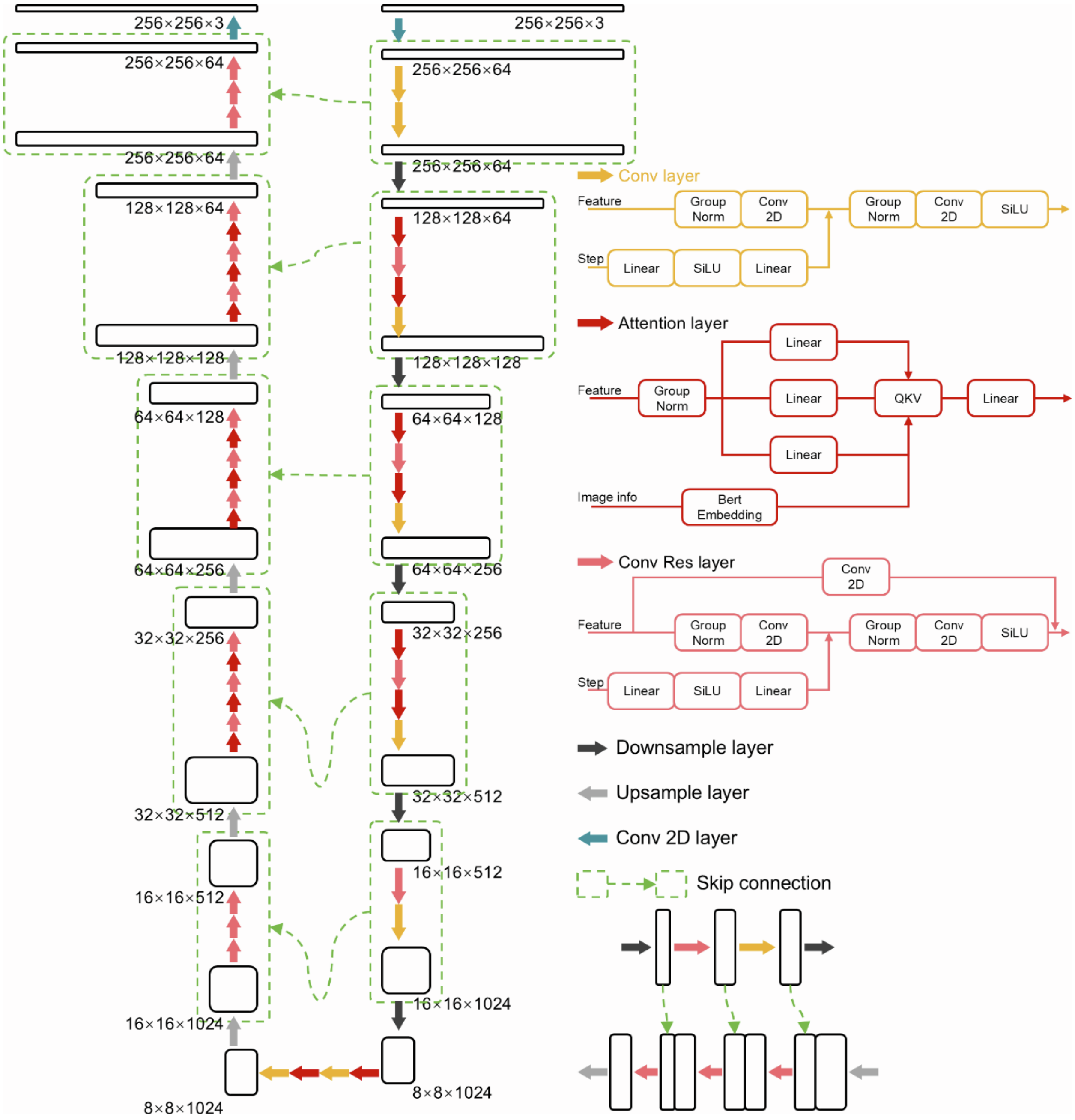

Fig. S3. Architecture of the visual module in Brainfound

The visual module adopts a U-shaped encoder–decoder architecture. Input images are progressively downsampled across encoder stages to aggregate multi-scale and global contextual features, and are subsequently upsampled in the decoder for image reconstruction. Textual conditioning is provided by a BERT-based text encoder, and image–text fusion is implemented via cross-attention layers that modulate the reconstruction process. The left panel shows the overall network architecture, and the right panel provides a legend explaining the icons used in the diagram.

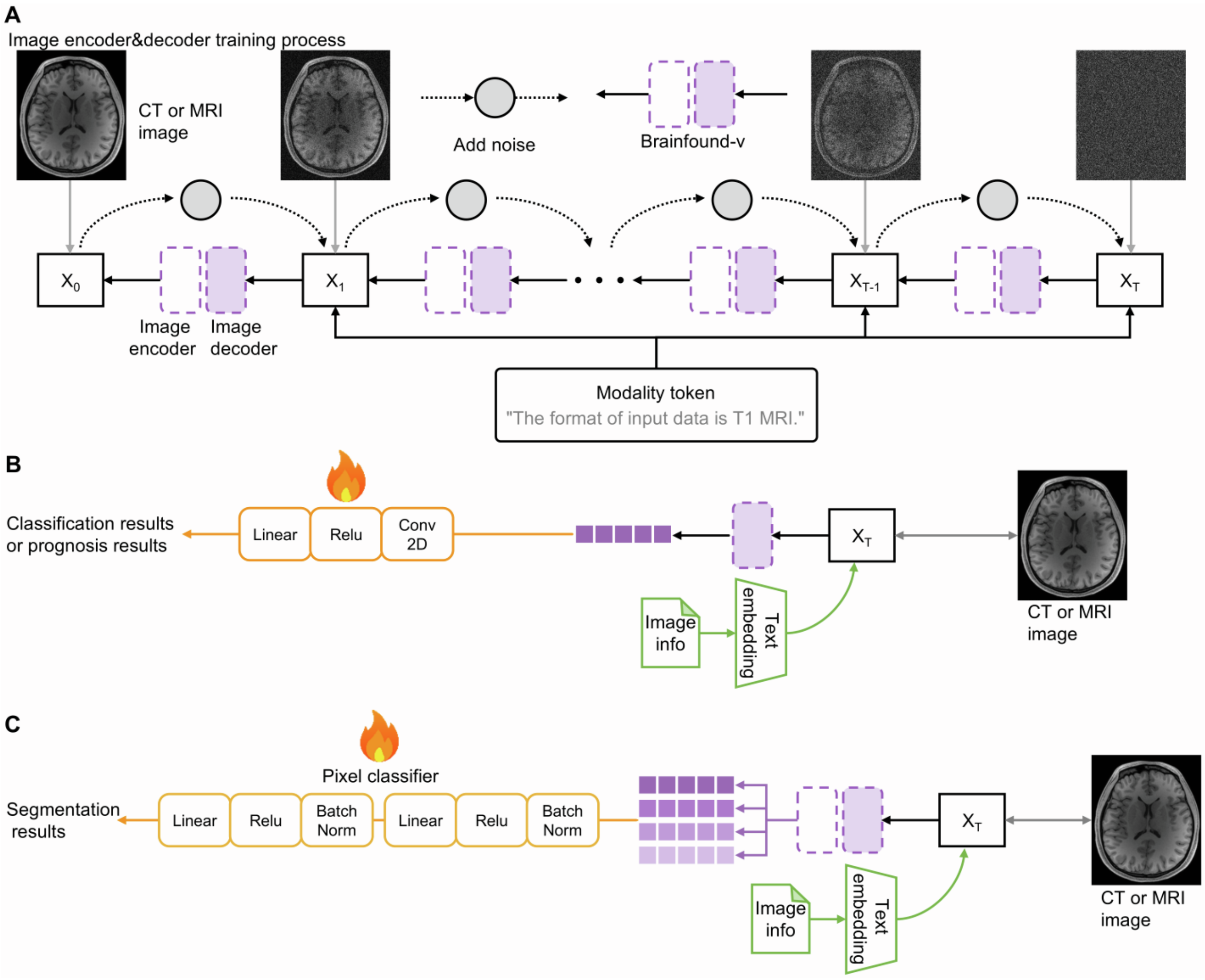

Fig. S4. Pre-training and task-specific fine-tuning of the Brainfound image encoder-decoder

(A) Pretraining of the image encoder–decoder following the DDPM framework. Clean brain CT or MRI images are progressively corrupted by additive noise to obtain noisy samples. The model is trained to denoise and reconstruct the original image from noise. A modality token (CT or MRI) is provided as a conditioning input to the visual network to guide reconstruction.

(B) Classification fine-tuning. Feature representations extracted by the pretrained Brainfound image encoder are fed into an MLP classifier to predict diagnostic labels. During downstream adaptation, the classifier head is fine-tuned using a limited amount of labeled data.

(C) Segmentation fine-tuning. Multi-scale features from the pretrained encoder–decoder are aggregated and passed to a pixel-wise classifier to produce lesion localization maps or segmentation masks. The pixel-wise classifier is fine-tuned with a limited amount of labeled data.

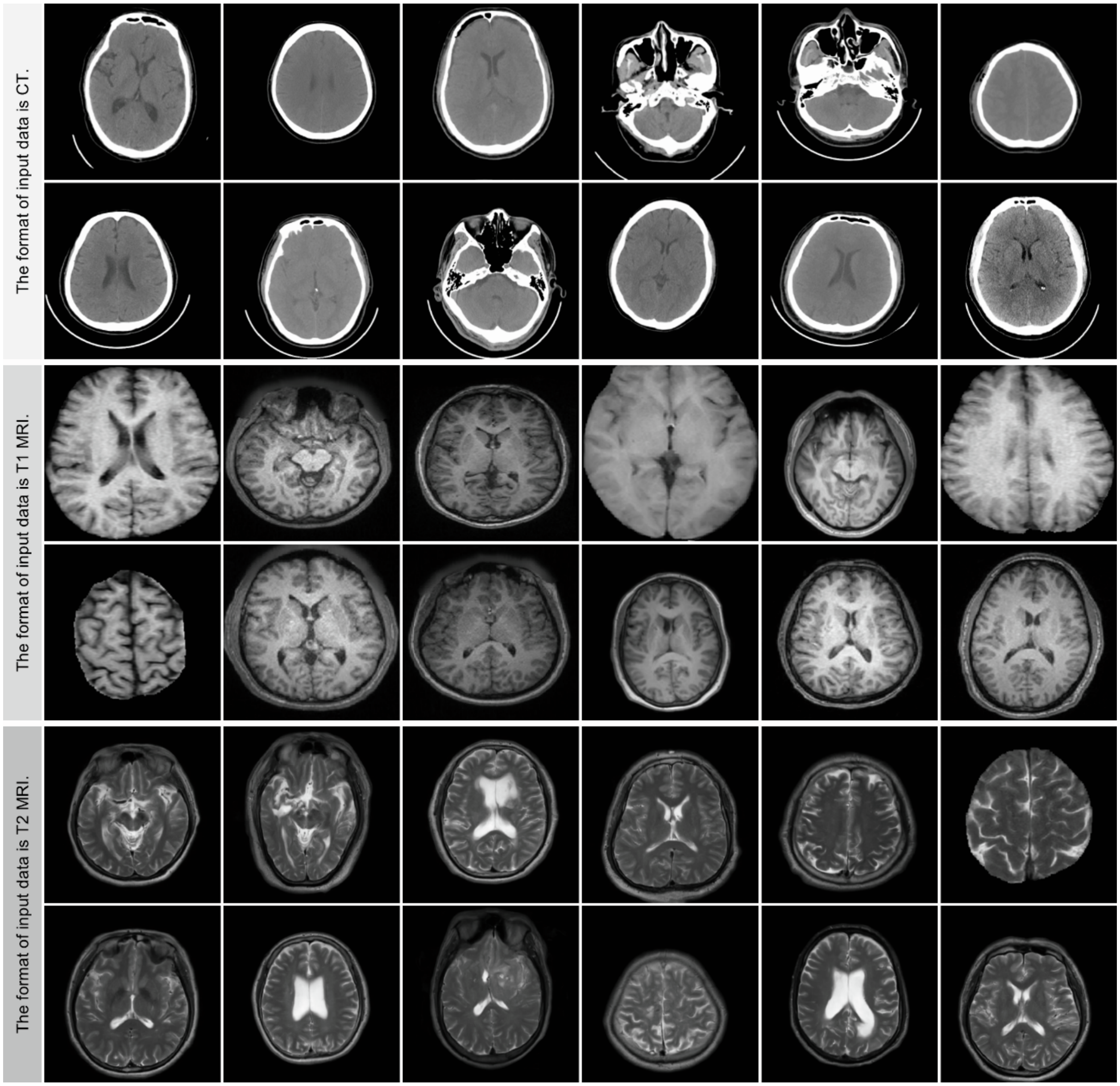

Fig. S5. Modality-conditioned image samples generated by the Brainfound vision module during diffusion pretraining

Representative generated samples are shown under modality conditioning. Brain CT, brain MRI T1WI, and brain T2WI are displayed in separate panels. Each modality panel contains 12 generated images.

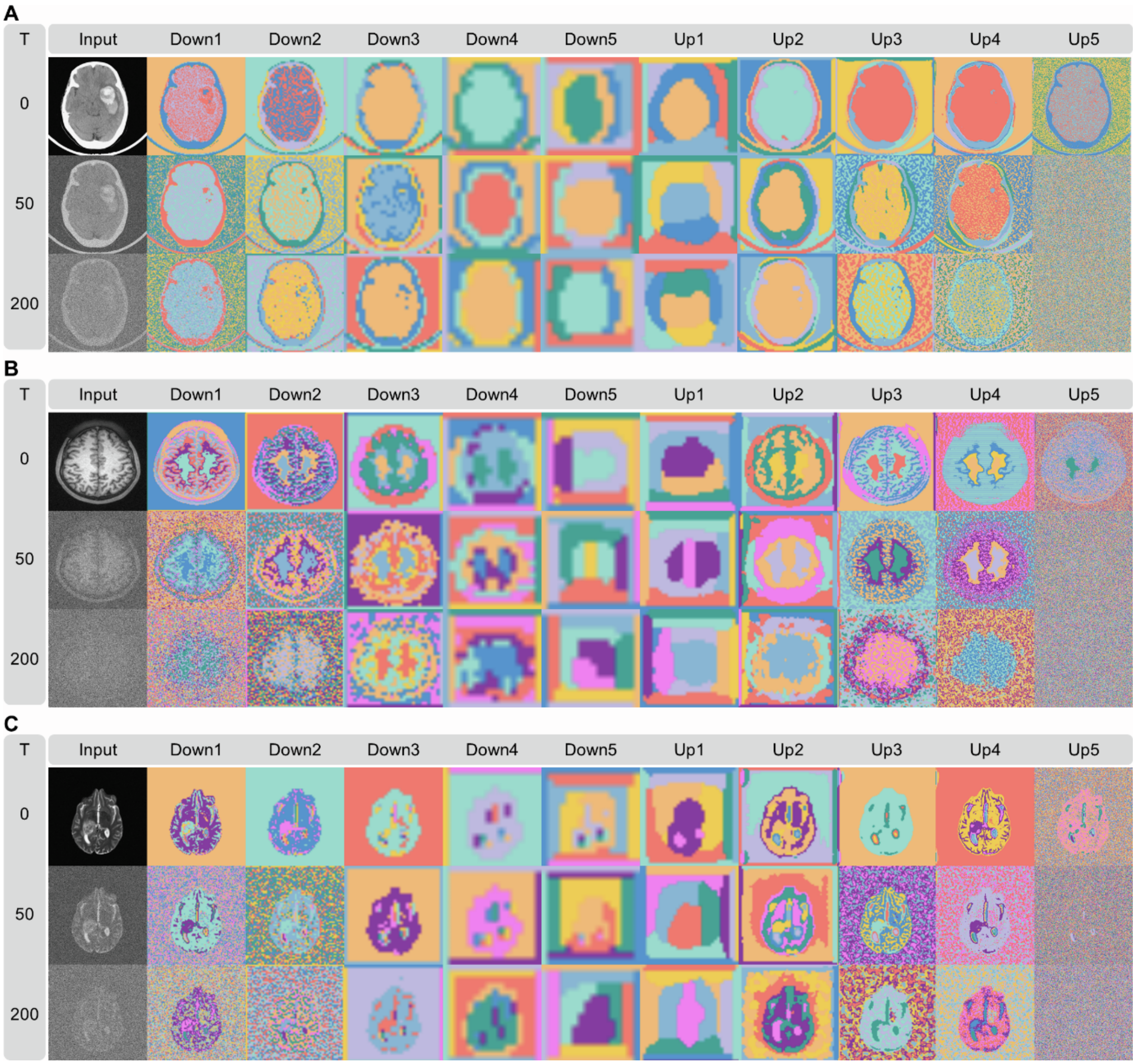

Fig. S6. Visualization of intermediate feature representations extracted by Brainfound

Feature maps are visualized from ten intermediate convolutional blocks of the diffusion U-shaped architecture, including five encoder stages (Down1 – Down5) and five decoder stages (Up1 – Up5), at three diffusion timesteps (T = 0, 50, 200). The first column shows the input images at the corresponding noise levels. Subsequent columns show k-means cluster assignment maps computed from the layer features (k = 10); each color denotes one cluster.

(A) using brain CT images as the input.

(B) using brain T1WI as the input.

(C) using brain T2WI as the input.

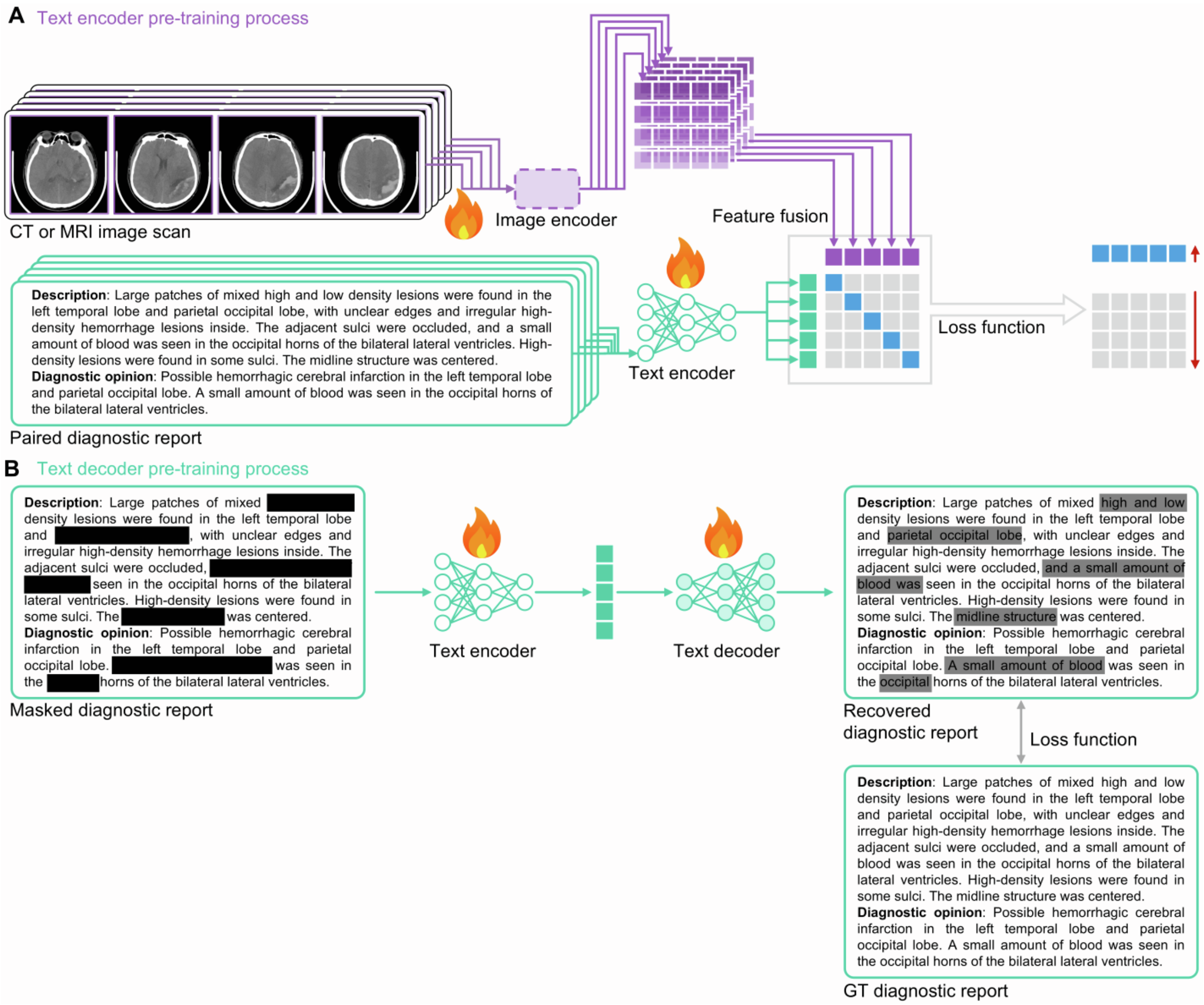

Fig. S7. Image‑text alignment of Brainfound

(A) CLIP-style image–text alignment during pretraining. Brain imaging studies are encoded by the image encoder to obtain image embeddings, and the corresponding clinical reports are encoded by the text encoder to obtain text embeddings. The two latent space features calculate cosine similarity for contrastive learning as a loss function. The contrastive loss function is applied to increase the similarity of matched image–report pairs and decrease the similarity of mismatched pairs within a batch.

(B) Pre-training of the text decoder. Clinical reports are corrupted by phrase- or sentence-level masking and passed through the text encoder. The text decoder reconstructs the complete report, and the reconstruction loss is computed against the original report text. During this stage, the text encoder is kept fixed, and only the text decoder parameters are updated.

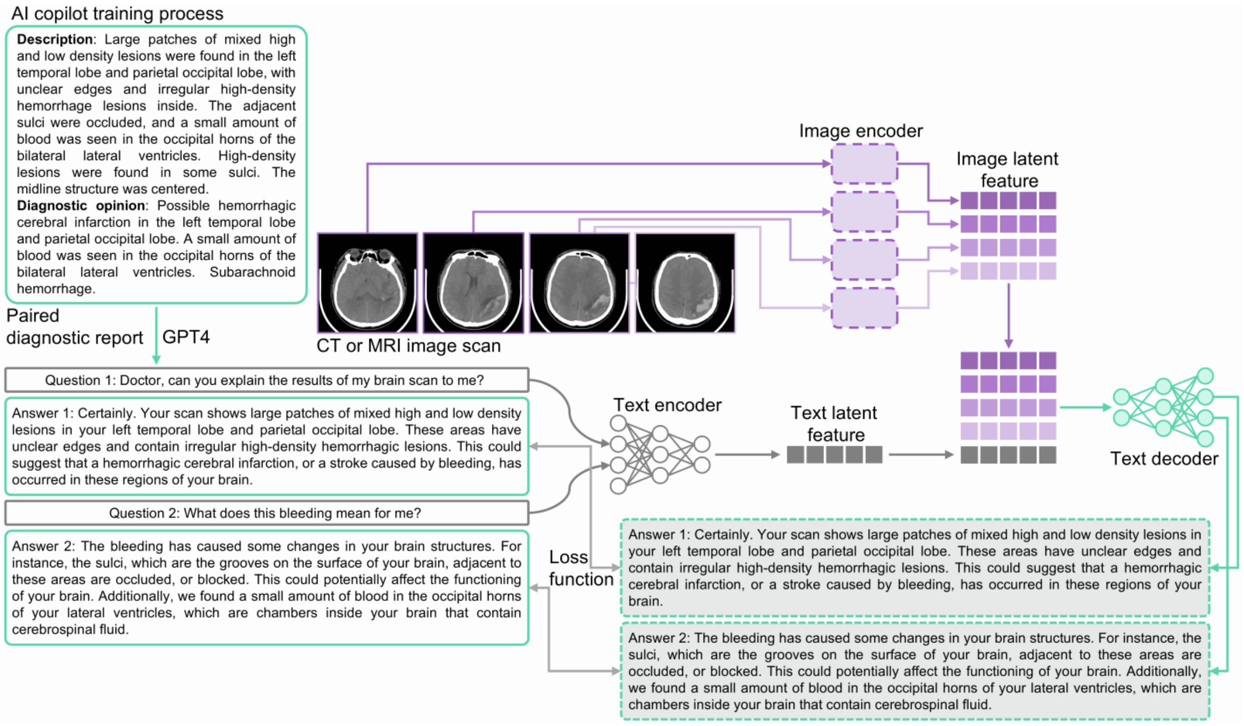

Fig. S8. Training pipeline for human-AI conversation in Brainfound

Given a diagnostic report, multi-turn open-ended dialogues are automatically generated using a large language model (GPT-4) to construct instruction–response pairs under diverse prompting templates. During training, user questions are encoded by the text encoder to produce text embeddings, and the corresponding imaging studies are encoded by the image encoder to produce image embeddings. The fused multimodal representation is provided to the text decoder, which predicts the response. The output of the text decoder and the answers during the conversation are used to compute the loss function, which subsequently guides the optimization of both the text encoder and decoder.

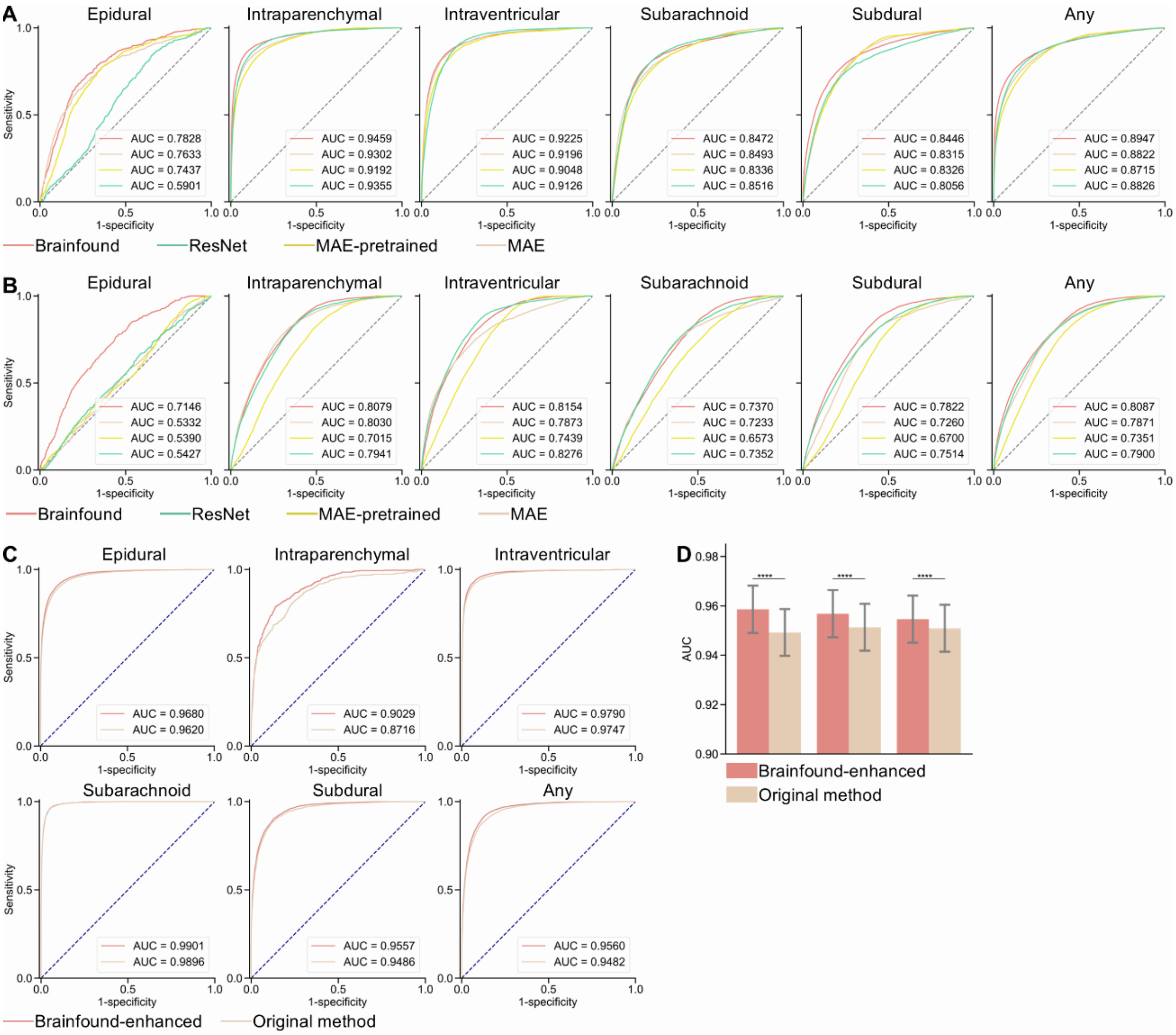

Fig. S9. Brain hemorrhage classification performance on the RSNA dataset

(A) ROC curves for six hemorrhage categories: Epidural, Intraparenchymal, Intraventricular, Subarachnoid, Subdural, and Any, after full-parameter fine-tuning of Brainfound and baseline models (ResNet, MAE-pretrained, and MAE). MAE-pretrained denotes an MAE model pretrained on BrainCT-3M, whereas MAE denotes an MAE model pretrained on a large-scale natural-image dataset.

(B) ROC curves for the same categories under a frozen-backbone setting, where the feature extractor is kept fixed, and only the classification head is fine-tuned for Brainfound and baselines. Other settings are identical to panel (A).

(C) ROC curve obtained by replacing the backbone in a high-performing RSNA competition pipeline with the pretrained Brainfound backbone.

(D) Summary results corresponding to panel (C). Each experiment was repeated three times with different random seeds, and performance is reported across runs.

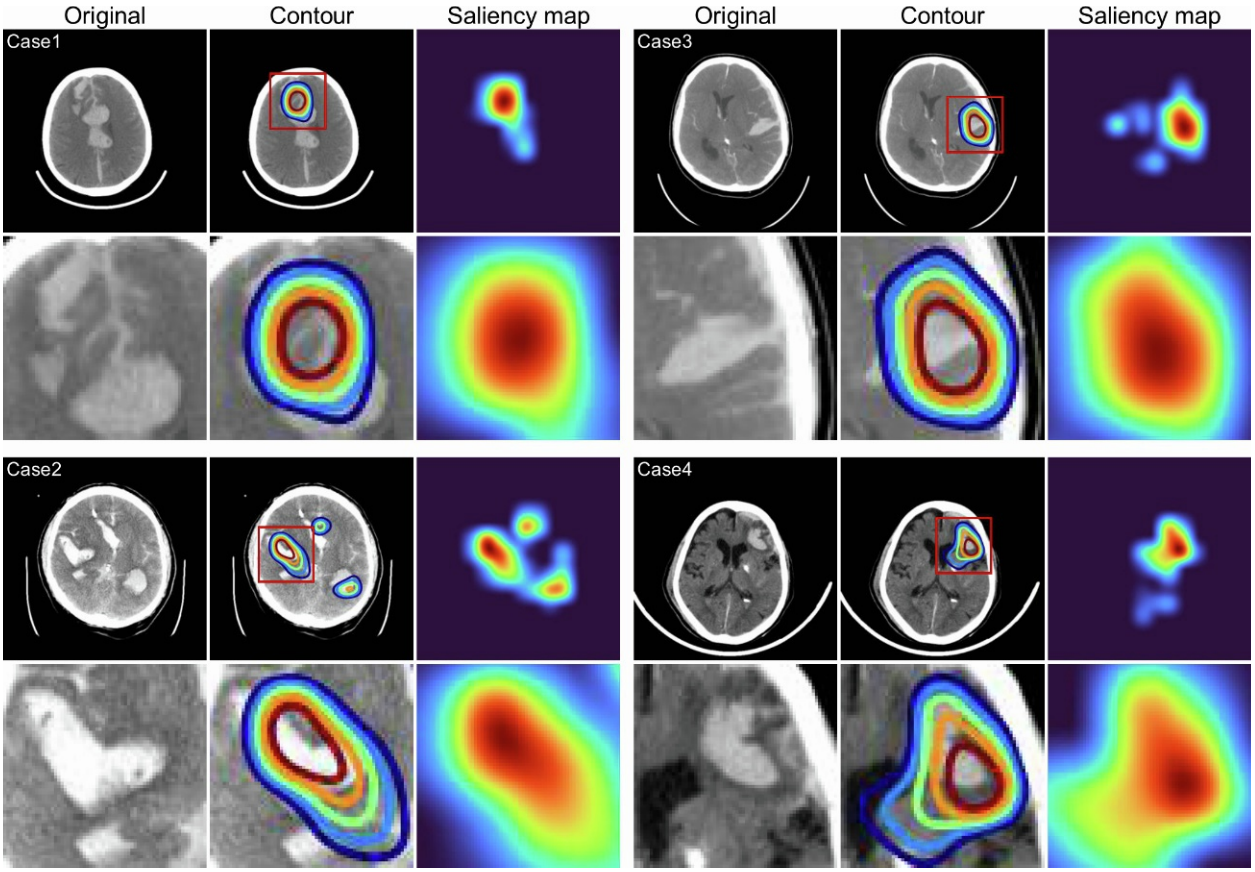

Fig. S10. Saliency maps produced by Brainfound for cerebral hemorrhage classification on the RSNA dataset

Representative saliency visualizations are shown for four brain CT images. For each example, the original CT image is shown in columns 1 and 4, the corresponding saliency contours are shown in columns 2 and 5, and the saliency heatmaps produced by Brainfound are shown in columns 3 and 6. Rows 2 and 4 provide enlarged views of the regions indicated by red boxes in rows 1 and 3, respectively.

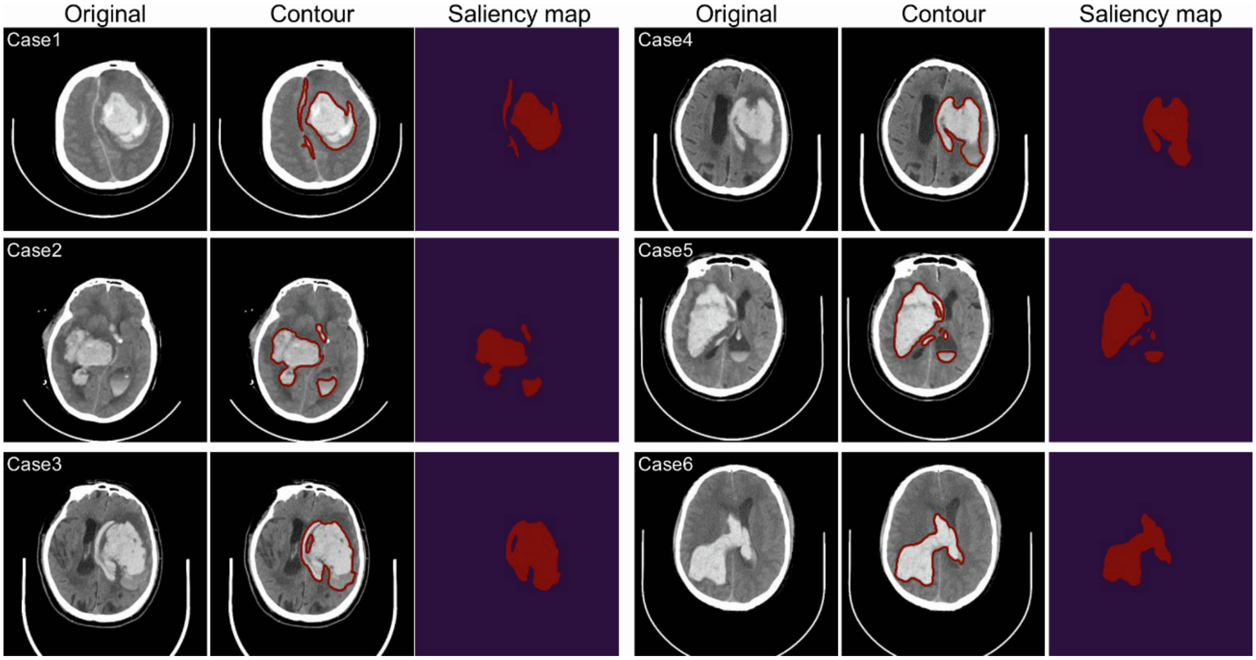

Fig. S11. Saliency maps generated by Brainfound for cerebral hemorrhage segmentation on the RSNA dataset

Representative saliency visualizations are shown for six brain CT images. For each example, the original CT image is shown in columns 1 and 4, the corresponding saliency outlines are shown in columns 2 and 5, and the saliency heatmaps generated by Brainfound are shown in columns 3 and 6.

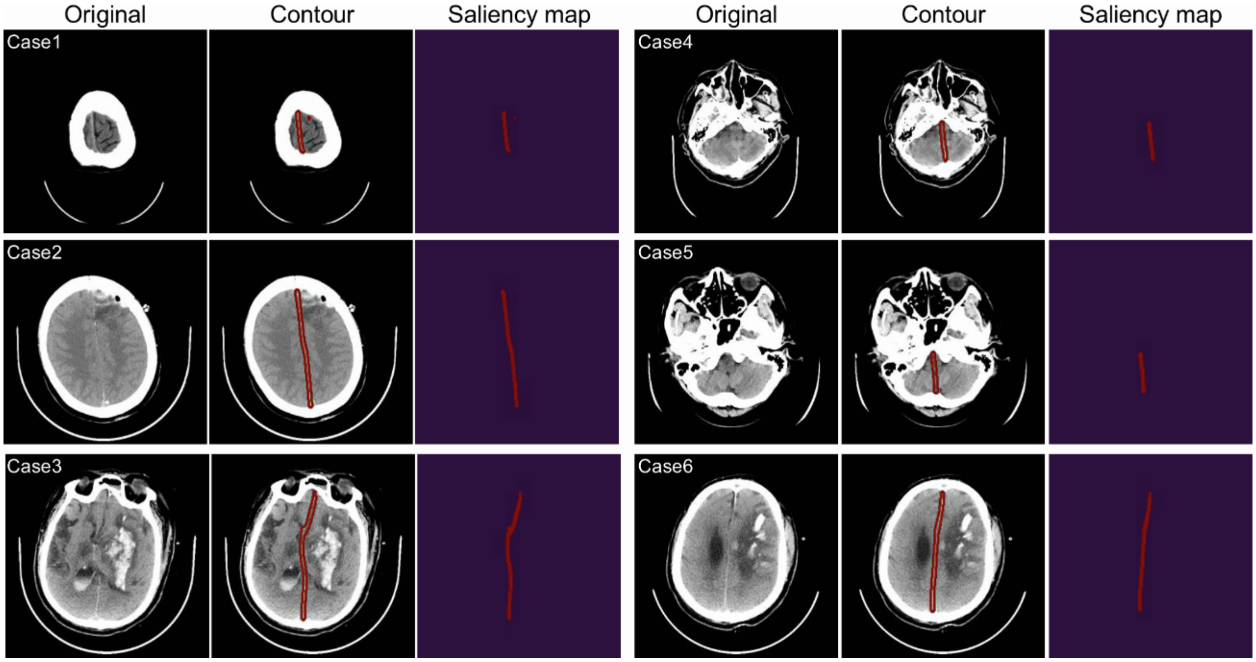

Fig. S12. Saliency maps generated by Brainfound for midline segmentation

Representative saliency visualizations are shown for six brain CT examples. For each example, the original CT image is shown in columns 1 and 4, the corresponding saliency outlines are shown in columns 2 and 5, and the saliency heatmaps generated by Brainfound are shown in columns 3 and 6.

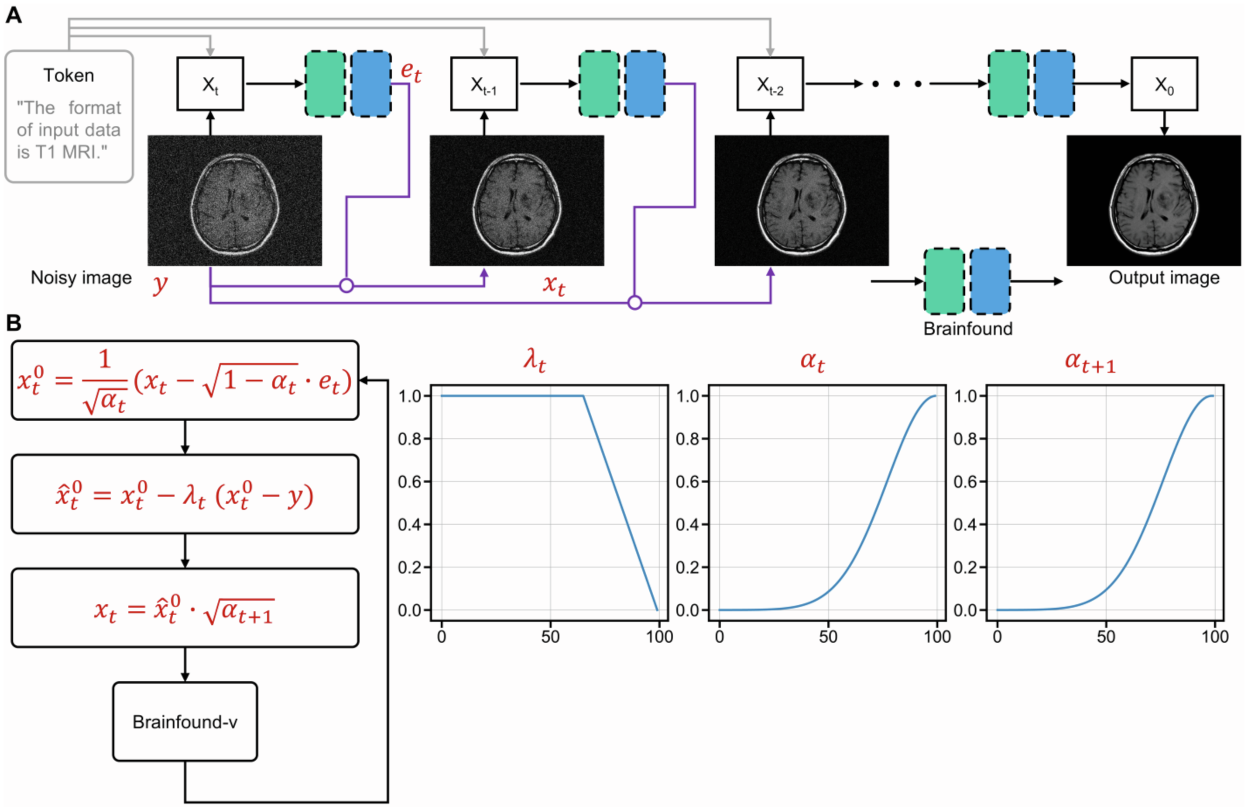

Fig. S13. Zero-shot denoising procedure of Brainfound

(A) Schematic illustration of the iterative denoising process. A noisy input image is incorporated into the DDPM based restoration procedure through repeated refinement steps, yielding a denoised output image. (B) Computation details for the iterative updates. The three curves in the right panel show the values of selected hyperparameters across iterations.

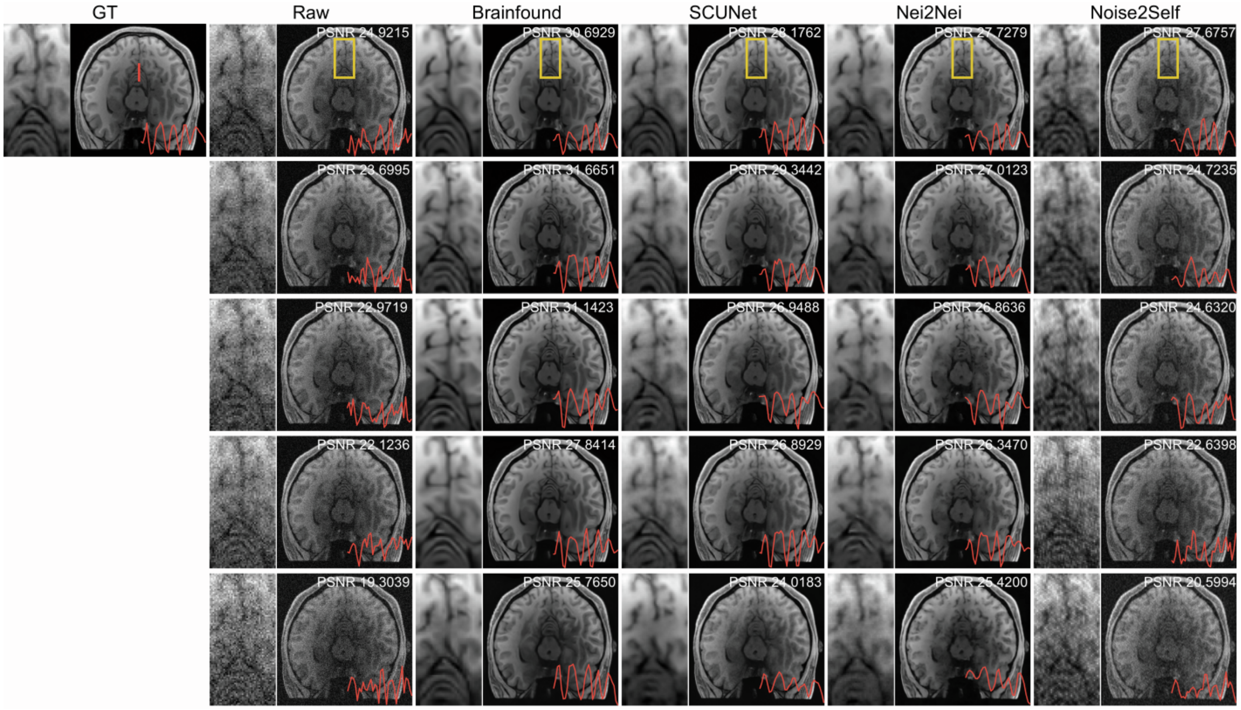

Fig. S14. Comparison of the denoising performance on 3T MRI images with simulated noise

Columns from left to right show the clean reference image, the noisy input image, the output of Brainfound, the output of SCUNnet, the output of Nei2Nei, and the output of Noise2Self. Rows 1 to 5 correspond to progressively increasing noise levels. The PSNR for each image is shown in the upper left corner. The region indicated by the yellow box is enlarged for visualization. Intensity profiles along the red line are shown for comparison.

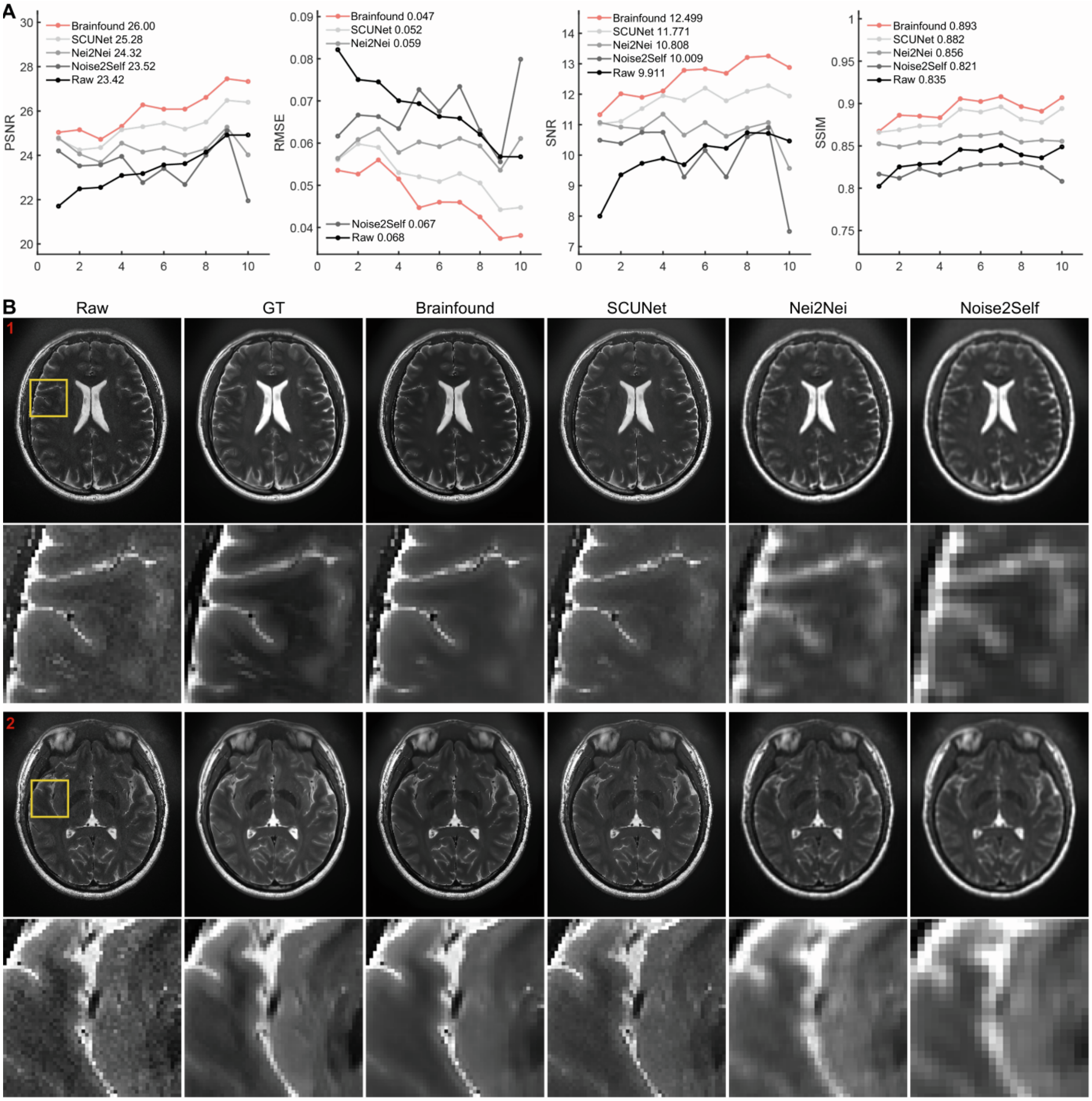

Fig. S15. The enhancement on 5T MRI T2 weighted images using Brainfound

(A) Quantitative comparison of Brainfound, SCUNet, Nei2Nei, and Noise2Self for MRI image enhancement on the test set captured by 5T MRI (n=10) at Beijing Friendship Hospital. Metrics are reported as PSNR, RMSE, SNR, and SSIM.

(B) Representative examples of 5T T2 weighted image denoising. Columns from left to right show the low SNR input, the high SNR reference, and the outputs of Brainfound, SCUNet, Nei2Nei, and Noise2Self. Regions indicated by yellow boxes in rows 1 and 3 are enlarged for visualization.

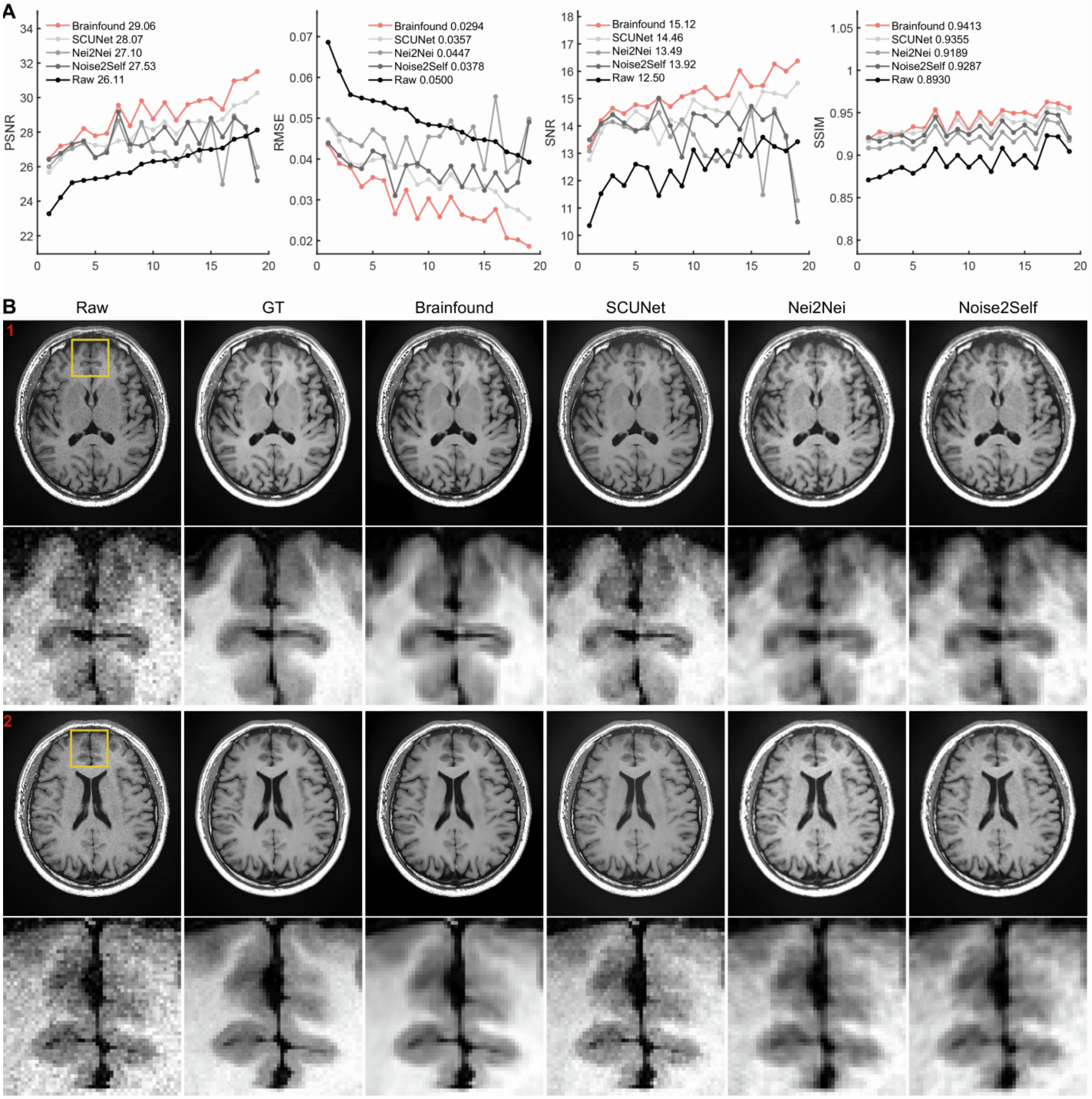

Fig. S16. The enhancement on 5T MRI T1 weighted images using Brainfound

(A) Quantitative comparison of Brainfound, SCUNet, Nei2Nei, and Noise2Self for MRI image enhancement on the external test dataset (n=19) captured by 5T MRI at Beijing Friendship Hospital. Metrics are reported as PSNR, RMSE, SNR, and SSIM.

(B) Representative examples of 5T T1 weighted image denoising. Columns from left to right show the low SNR input, the high SNR reference, and the outputs of Brainfound, SCUNet, Nei2Nei, and Noise2Self. Regions indicated by yellow boxes in rows 1 and 3 are enlarged for visualization.

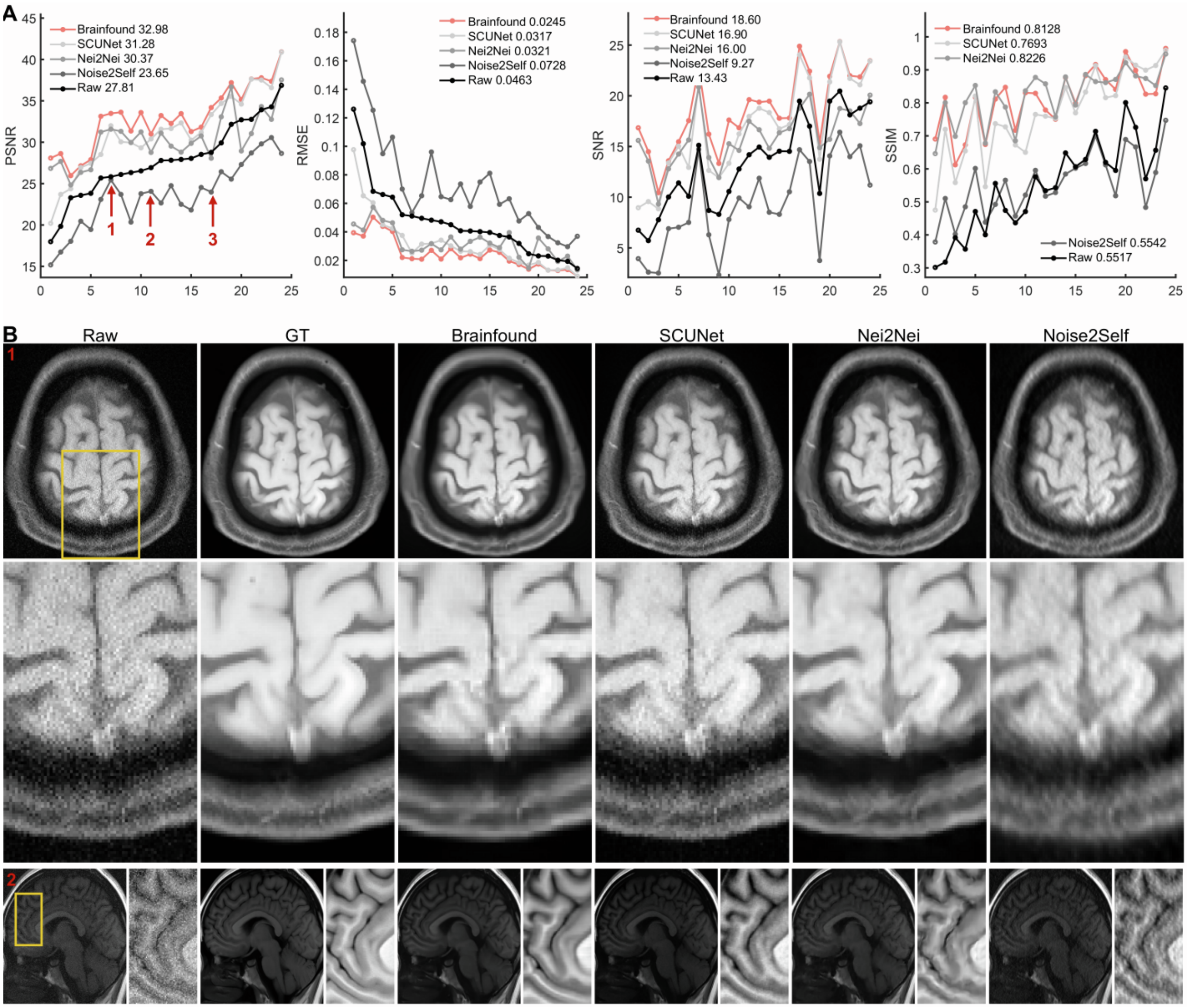

Fig. S17. The enhancement on real world 5T MRI images from an external center

(A) Quantitative comparison of Brainfound, SCUNet, Nei2Nei, and Noise2Self for MRI image enhancement on the external test dataset (n=25) captured at the Shanghai United Imaging center. Metrics are reported as PSNR, RMSE, SNR, and SSIM.

(B) Representative examples corresponding to the two cases indicated by arrows in panel (A). Displayed from left to right are the original noisy image, the high SNR GT image, the image enhanced by Brainfound, the image enhanced by SCUNet, the image enhanced by Nei2Nei, and the image enhanced by Noise2Self. The region outlined by the yellow box is enlarged for visualization.

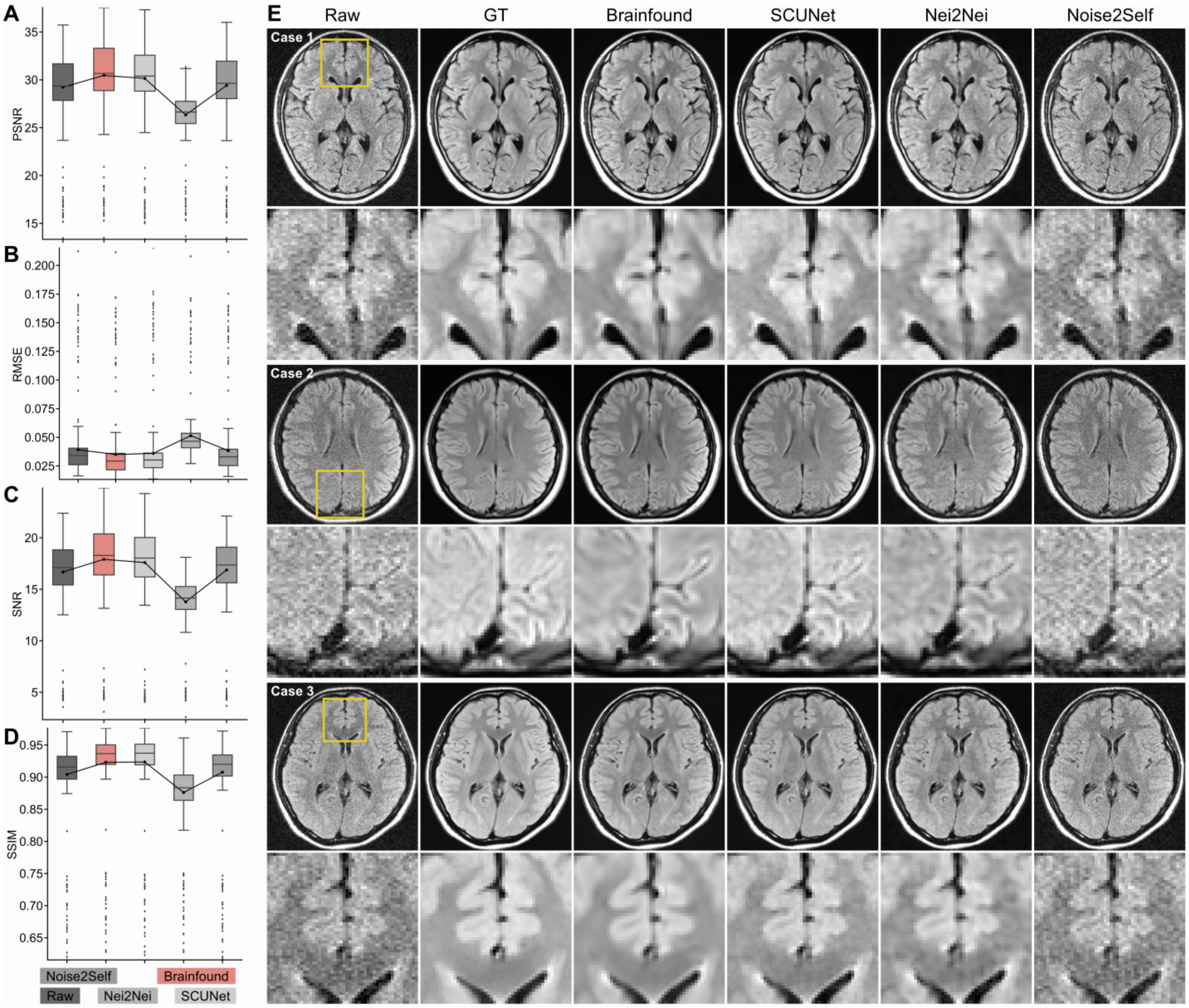

Fig. S18. Comparison of zero-shot enhancement on 0.3T FLAIR images

(A-D) Quantitative comparison of image enhancement results using four methods: Brainfound, Noise2Self, Nei2Nei, SCUNet on the 0.3T FLAIR dataset (with n=450). PSNR, RMSE, SNR, and SSIM were evaluated, respectively. Brainfound achieved the best scores in all metrics except SSIM.

(E) Representative denoising examples. From left to right: the original image, high SNR reference image, Brainfound-enhanced image, SCUNet-enhanced image, Nei2Nei-enhanced image, and Noise2Self-enhanced image. The region indicated by the yellow box is enlarged for visualization.

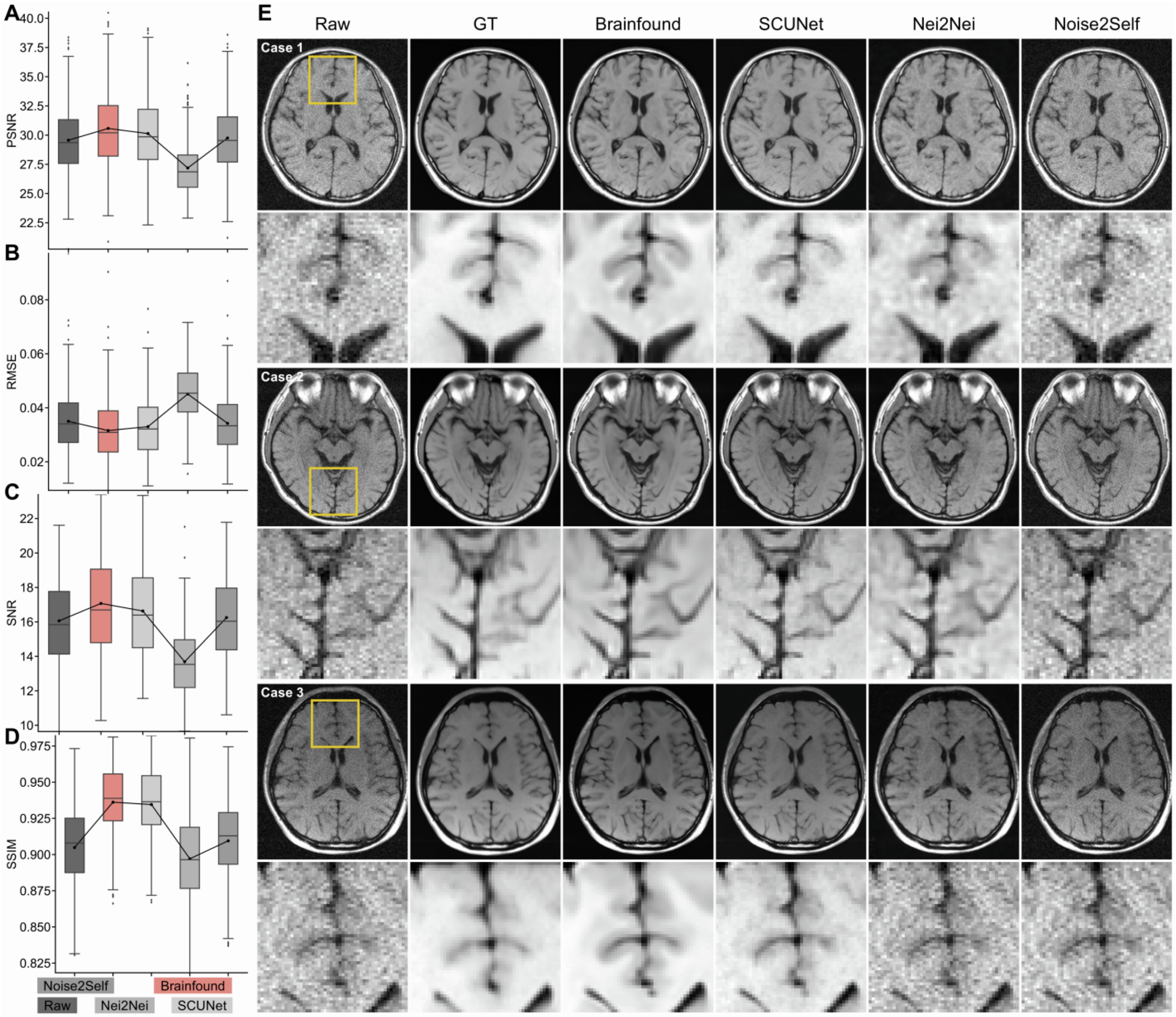

Fig. S19. Comparison of zero-shot enhancement on 0.3T T1WI

(A-D) Quantitative comparison of image enhancement results using four methods: Brainfound, Noise2Self, Nei2Nei, SCUNet on the 0.3T T1 weighted dataset (with n=450). PSNR, RMSE, SNR, and SSIM were respectively computed. Brainfound achieved the best scores in all metrics.

(E) Representative denoising examples. From left to right: the original image, high SNR reference image, Brainfound-enhanced image, SCUNet-enhanced image, Nei2Nei-enhanced image, and Noise2Self-enhanced image. The yellow-boxed area is enlarged for display.

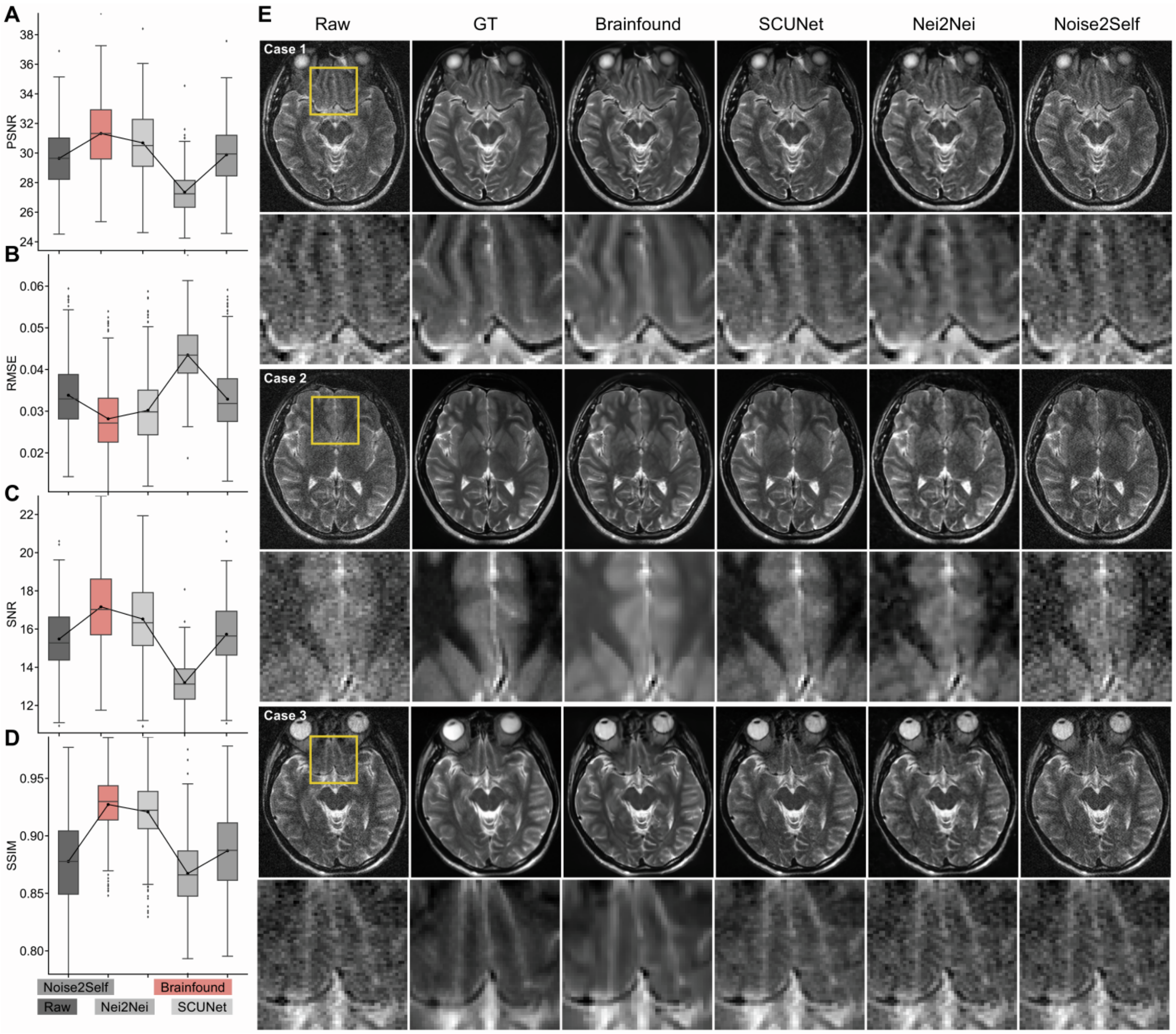

Fig. S20. Comparison of zero-shot enhancement on 0.3T T2WI

(A-D) Quantitative comparison of image enhancement results using four methods: Brainfound, Noise2Self, Nei2Nei, SCUNet (with n=450). PSNR, RMSE, SNR, and SSIM were respectively calculated. Brainfound achieved the best scores in all metrics.

(E) Representative denoising examples. From left to right: the original image, high SNR reference image, Brainfound-enhanced image, SCUNet-enhanced image, Nei2Nei-enhanced image, and Noise2Self-enhanced image. The yellow-boxed area is enlarged for display.

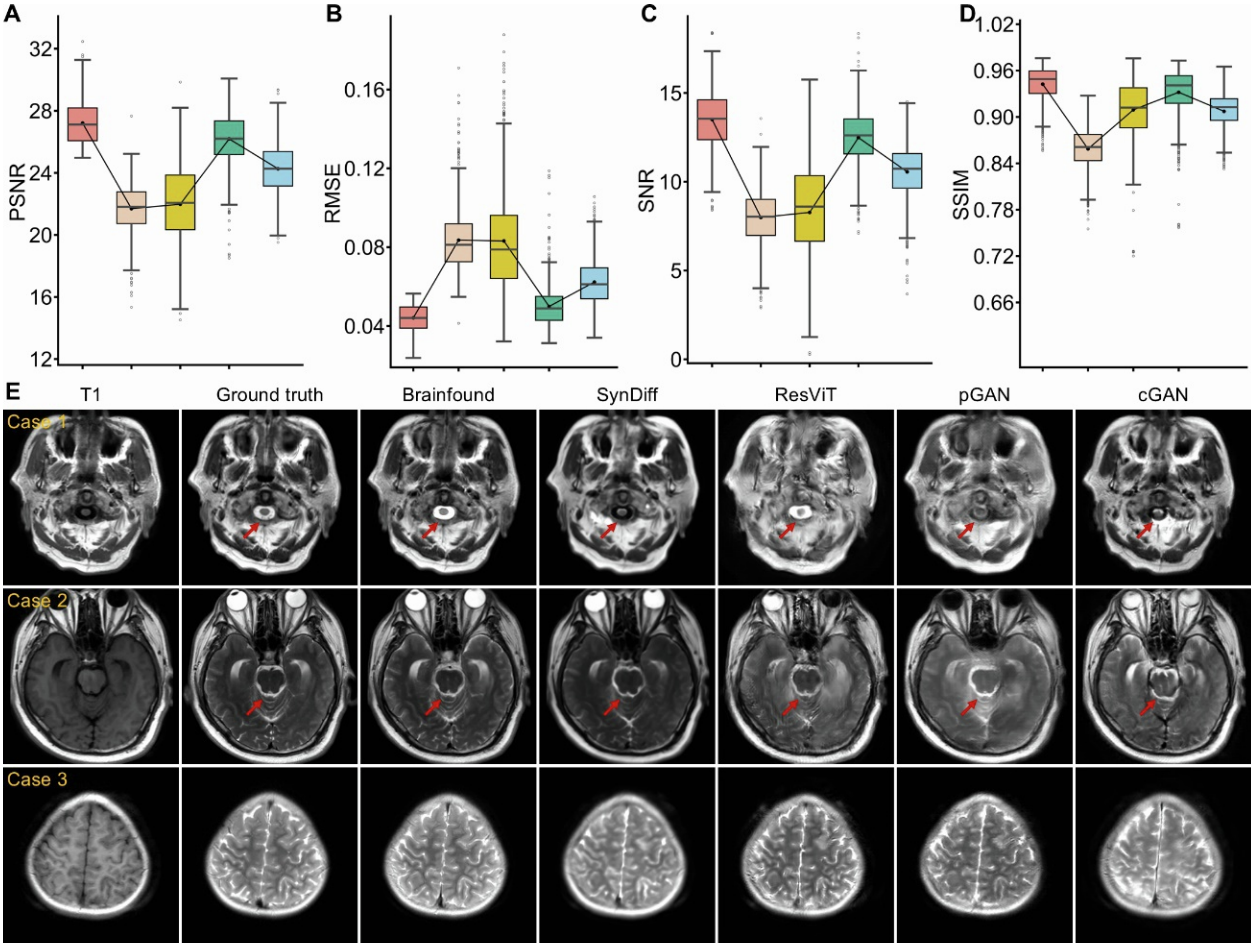

Fig. S21. Performance of Brainfound for MRI modality translation from T1WI to T2WI

(A-D) Quantitative evaluation of image modality translation with Brainfound, SynDiff, ResViT, pGAN, and cGAN (n=1936). Metrics include PSNR, RMSE, SNR, and SSIM.

(E) Representative T1 to T2 translation examples. From left to right: original T1WI, paired T2WI, results from Brainfound, SynDiff, ResViT, pGAN, and cGAN. In case 1, Brainfound accurately identifies the cerebrospinal fluid (CSF) around the spinal cord that shows low signal intensity on T1WI and converts these areas into high signal intensity on generated T2WI. In case 2, Brainfound generated a clearer image in which the pons and their surrounding structures look sharp and have good contrast. In case 3, Brainfound achieves a higher resolution in conversion tasks, while other methods result in slightly blurry images.

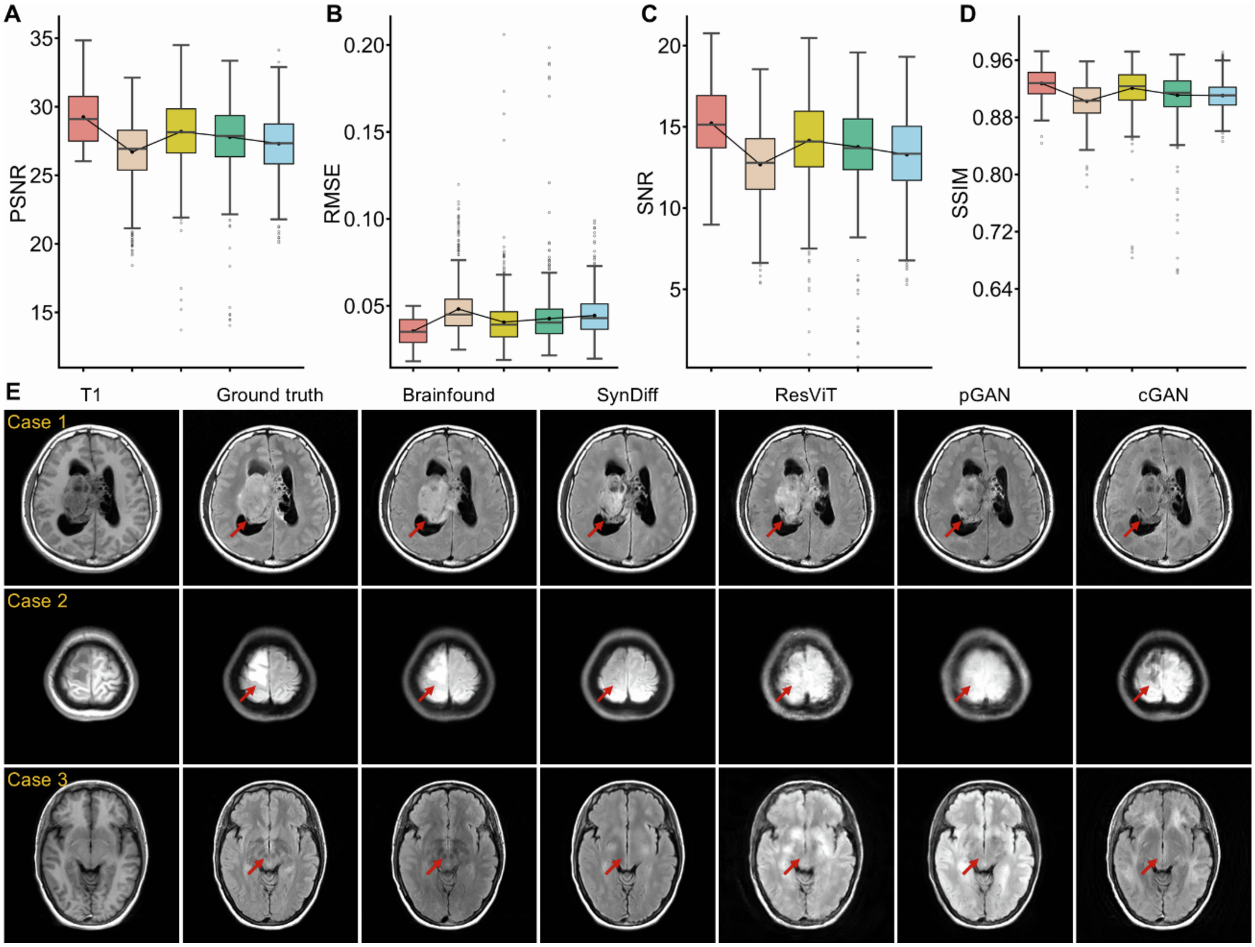

Fig. S22. Performance of Brainfound for MRI modality translation from T1WI to FLAIR

(A-D) Image modality translation was assessed quantitatively with Brainfound, SynDiff, ResViT, pGAN, and cGAN (n=1936). Brainfound showed superior performance across all metrics, including PSNR, RMSE, SNR, and SSIM.

(E) Three cases of T1WI-to-FLAIR image translation via five methods. In case 1, Brainfound more effectively transformed the situation of the tumor. In case 2, Brainfound accurately converted the edema lesion with low signal intensity on T1WI into high signal intensity on FLAIR. In case 3, Brainfound accurately generated the distinct darkened areas of the red nucleus and substantia nigra on the FLAIR image.

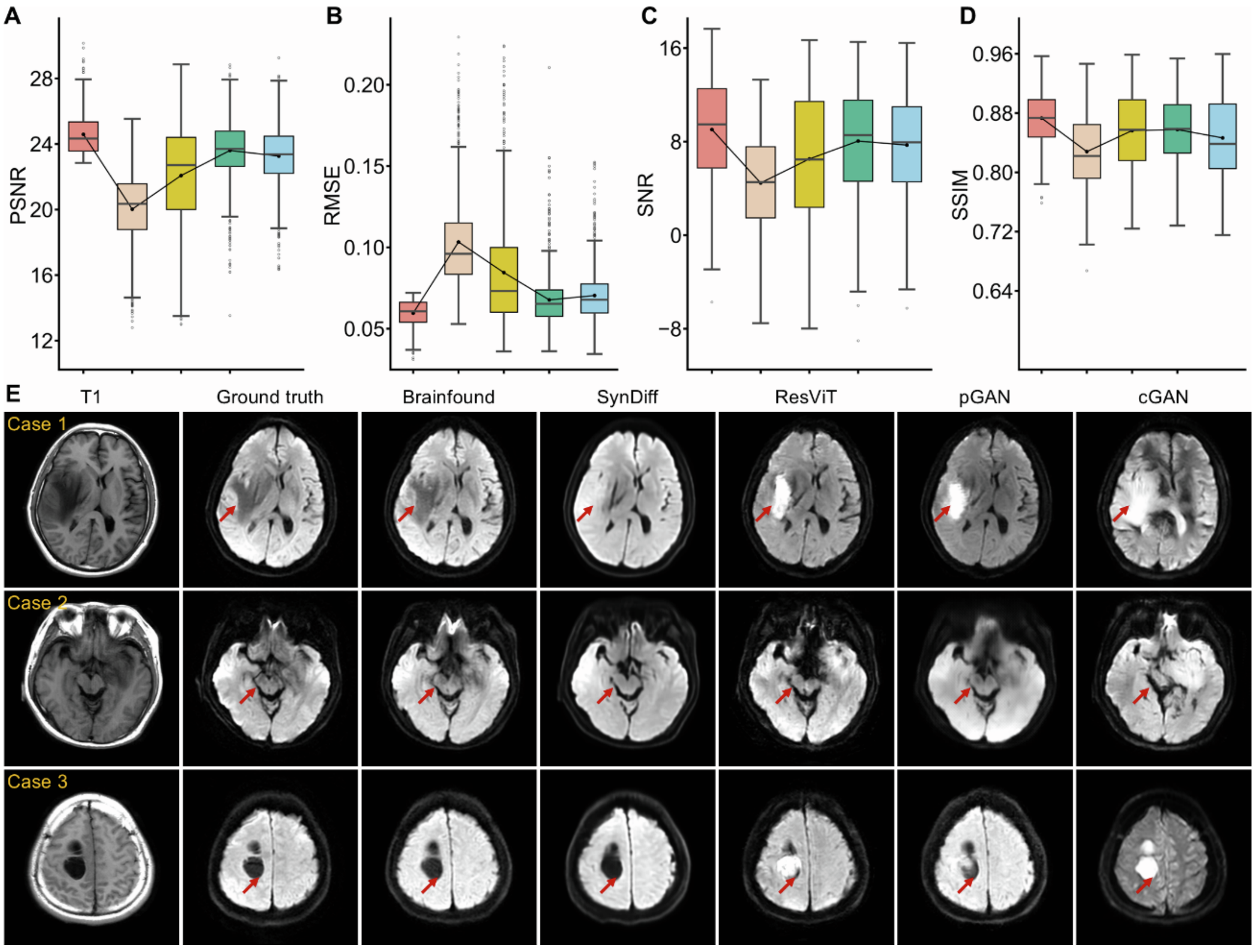

Fig. S23. Performance of Brainfound for MRI modality translation from T1WI to standard-b-value DWI

(A-D) Quantitative assessment of T1WI to standard-b-value DWI image translation results was carried out using five methods: Brainfound, SynDiff, ResViT, pGAN, and cGAN (n=1936). The metrics PSNR, RMSE, SNR, and SSIM were calculated, with Brainfound obtaining the best performance in all metrics.

(E) Three cases of T1WI to standard-b-value DWI Image translation via five methods. For case 1 and case 3, the peritumoral vasogenic edema and the cystic lesions typically do not show restricted diffusion (high signal) on DWI images, and Brainfound accurately identified the edema and cystic regions and output the images with corresponding hypointense lesions. In case 2, the images generated by Brainfound exhibit less distortion in the slices near the base of the skull, and the depiction of the brainstem, ambient cisterns, and medial temporal lobes is clearer and matches the ground truth better than the other models.

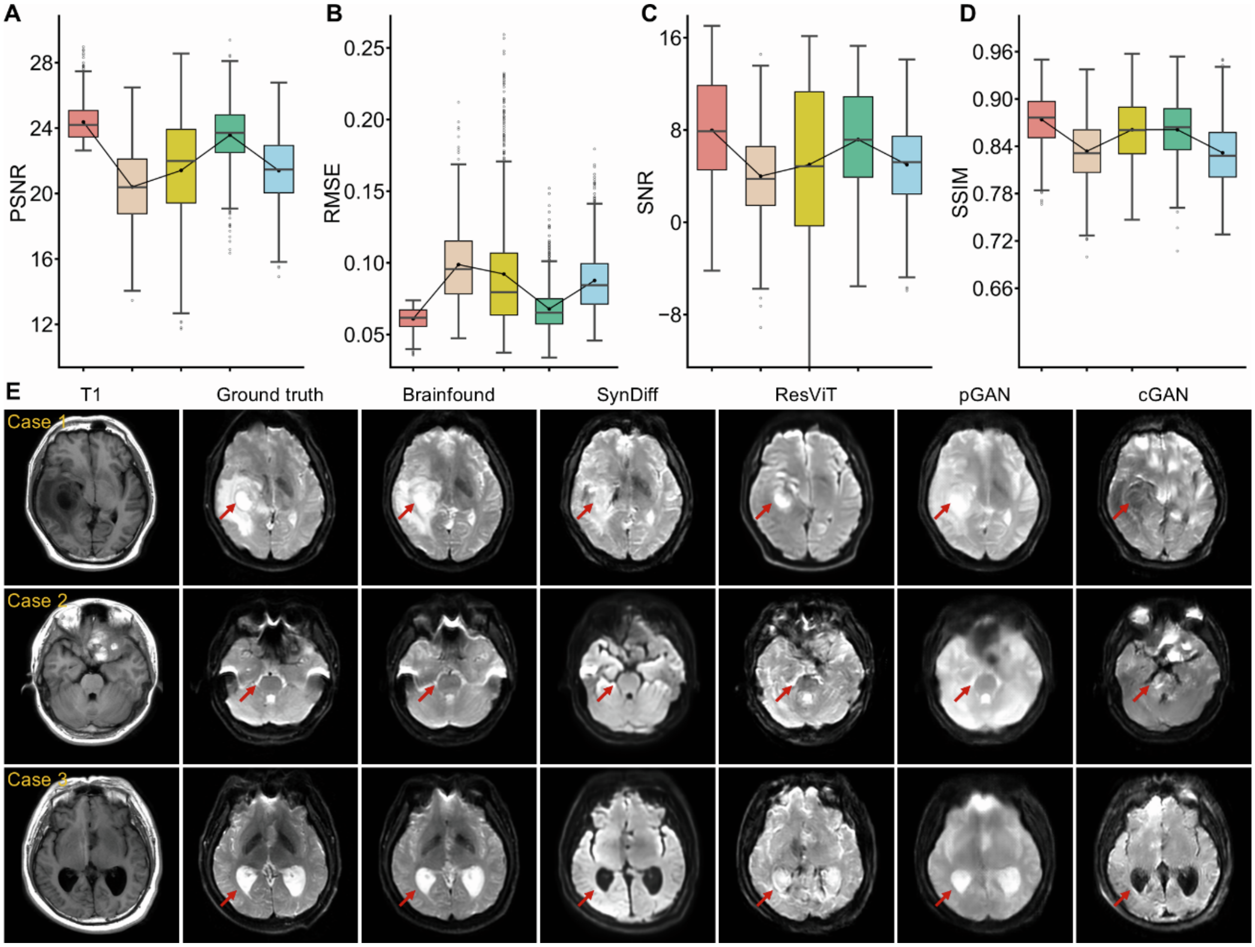

Fig. S24. Performance of Brainfound for MRI modality translation from T1WI to low-b-value DWI

(A-D) Quantitative comparison was made of T1WI to low-b-value DWI image translation results using five methods: Brainfound, SynDiff, ResViT, pGAN, and cGAN (n=1936). PSNR, RMSE, SNR, and SSIM were calculated individually. Brainfound achieved the highest scores in all metrics.

(E) Three cases of T1WI to low-b-value DWI Image translation via five models. As shown by the red arrows, the vasogenic edema and cystic lesions (case 1) and the CSF in the lateral ventricles (case 3) were accurately transformed into the high signal from the low signal on the original T1WI by Brainfound. In case 2, Brainfound provides a conversion that is closest to the ground truth.

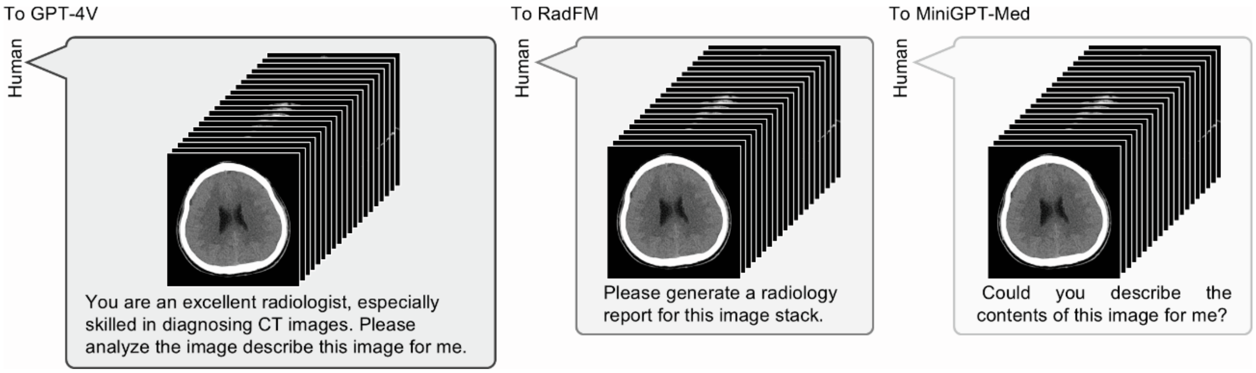

Fig. S25. Prompts used for radiology report generation

Columns from left to right show the prompts used for GPT 4V, RadFM, and MiniGPT-Med.

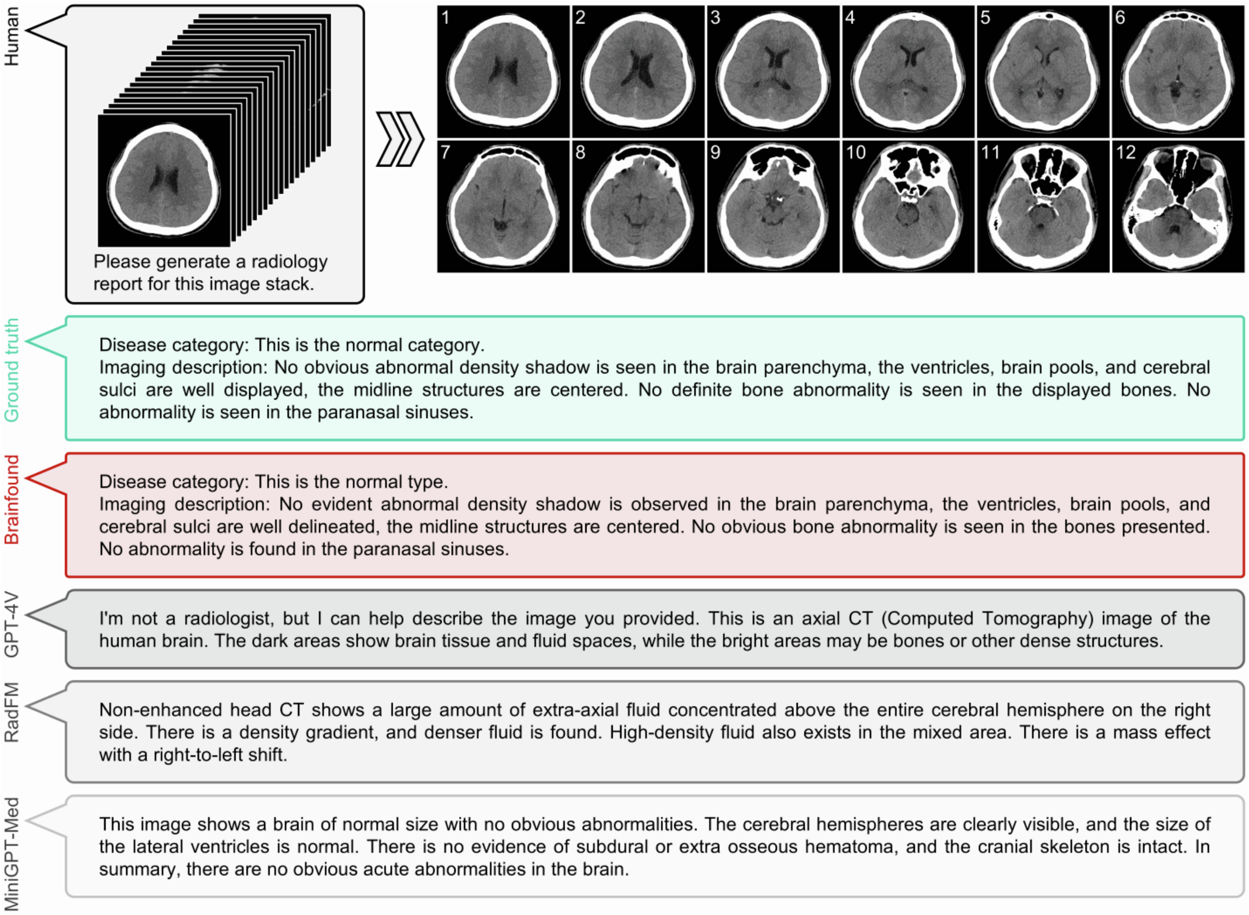

Fig. S26. Report generation example for a normal brain CT scan

The top panel shows the input CT scan and the prompt employed for report generation. A set of 12 representative slices from the same scan is shown on the right. The bottom panel presents the reference report written by an experienced doctor, together with reports generated by Brainfound, GPT4-V, RadFM, and MiniGPT-Med. Differences across generated reports are illustrated in terms of clinical formatting, terminology, and internal consistency.

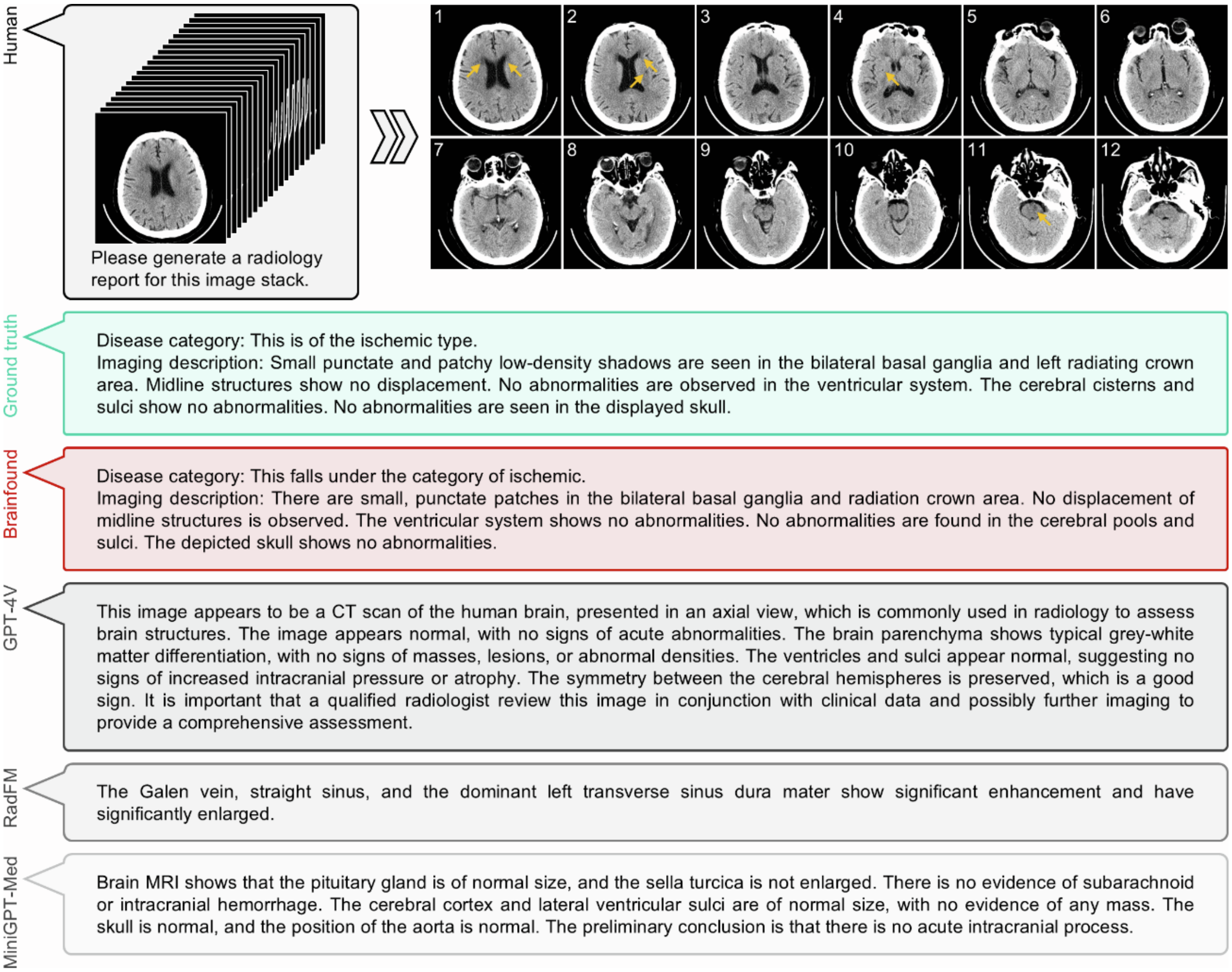

Fig. S27. Report generation example for an ischemic brain CT scan

The top panel shows the input CT scan and the prompt used in the examination. On the right, there are 12 typical brain CT images from the scan. Subsequently, reports crafted by an experienced doctor, along with those formulated by Brainfound, GPT4-V, RadFM, and MiniGPT-Med, are displayed. In this example, the RadFM output provides limited coverage of key imaging findings, and the MiniGPT Med output incorrectly refers to the input as an MRI sequence.

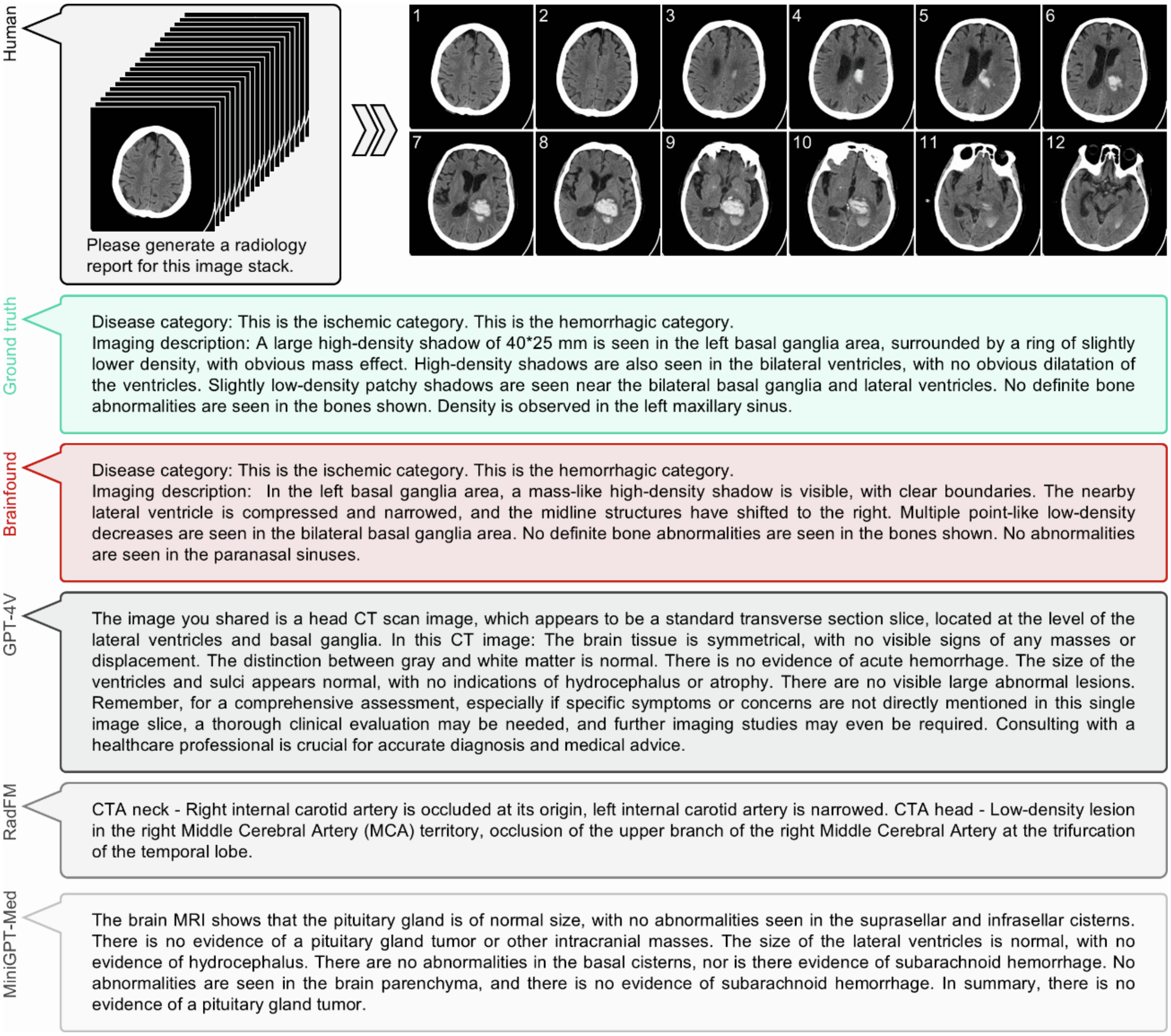

Fig. S28. Report generation example for a hemorrhagic brain CT scan

The upper portion of the display illustrates the input CT scan and the prompt used in the examination. On the right, there are 12 typical CT images from the scan. Subsequently, reports crafted by an experienced doctor, along with those formulated by Brainfound, GPT4-V, RadFM, and MiniGPT-Med, are showcased. In this example, the GPT 4V output does not explicitly list the diagnoses described in the reference report. The RadFM output refers to the input as a CTA study, and the MiniGPT Med output refers to the input as an MRI sequence.

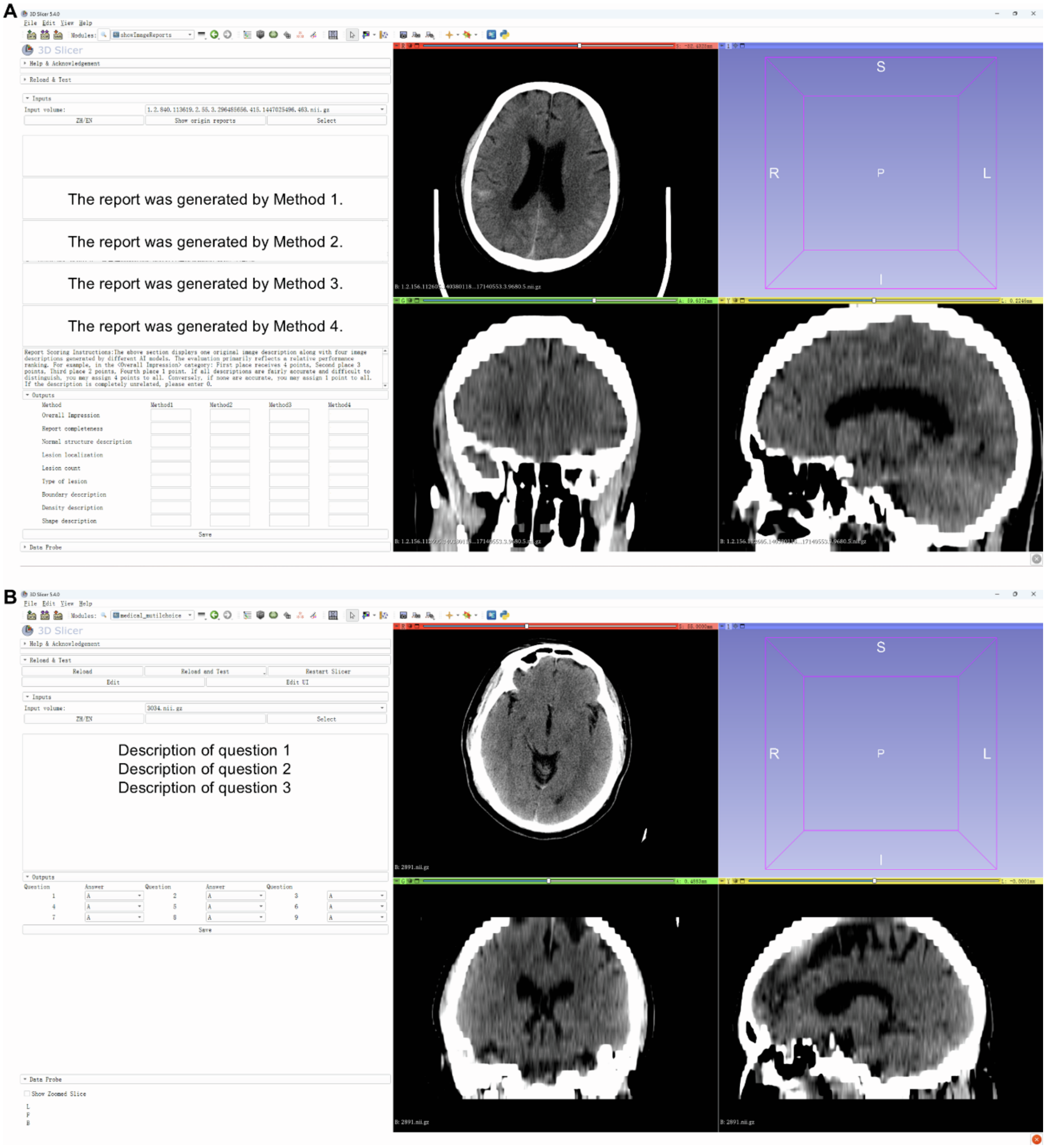

Fig. S29. The report evaluation and MCQ answering interfaces implemented in 3D Slicer

(A) Screenshot of the report evaluation interface. The CT scan associated with the current report is visualized in a three dimensional view, and reports generated by four methods are displayed for expert review together with a reference report written by an experienced radiologist. Report quality is rated across nine evaluation dimensions.

(B) Screenshot of the multiple choice question answering interface. This panel delineates three MCQs, each with multiple answer choices, where doctors are prompted to input their selections at the designated lower segment of the interface. For more details, refer to https://github.com/gingerbread000/SlicerMedicalReportGrading.

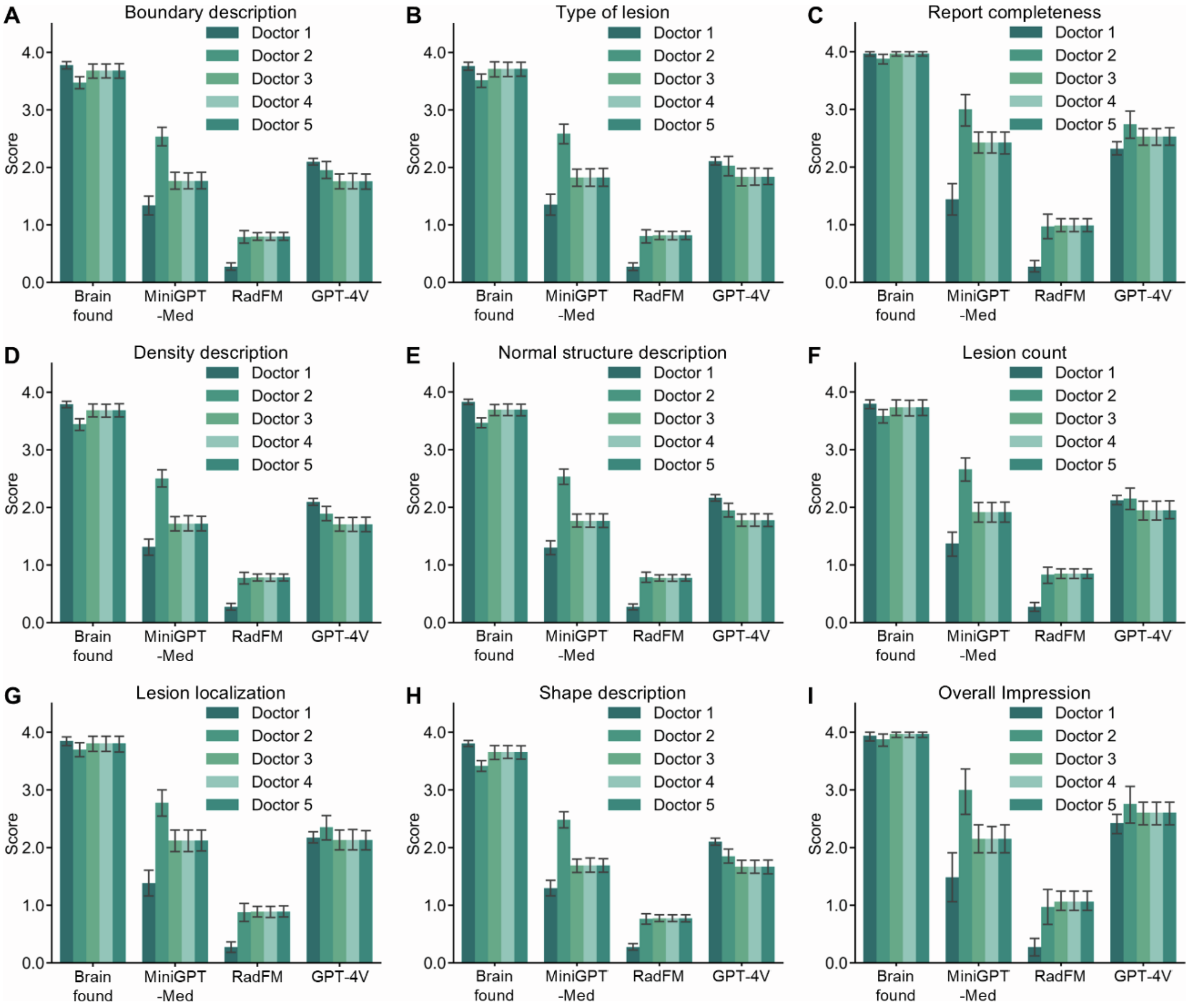

Fig. S30. Expert scoring of reports generated by four methods across nine evaluation dimensions

(A) Boundary description.
(B) Lesion type.
(C) Report completeness.
(D) Density description.
(E) Description of normal structures.
(F) Lesion count.
(G) Lesion localization.
(H) Shape description.
(I) Overall impression. Scores were provided by five experienced clinicians with a mean of 6.4 years of practice.

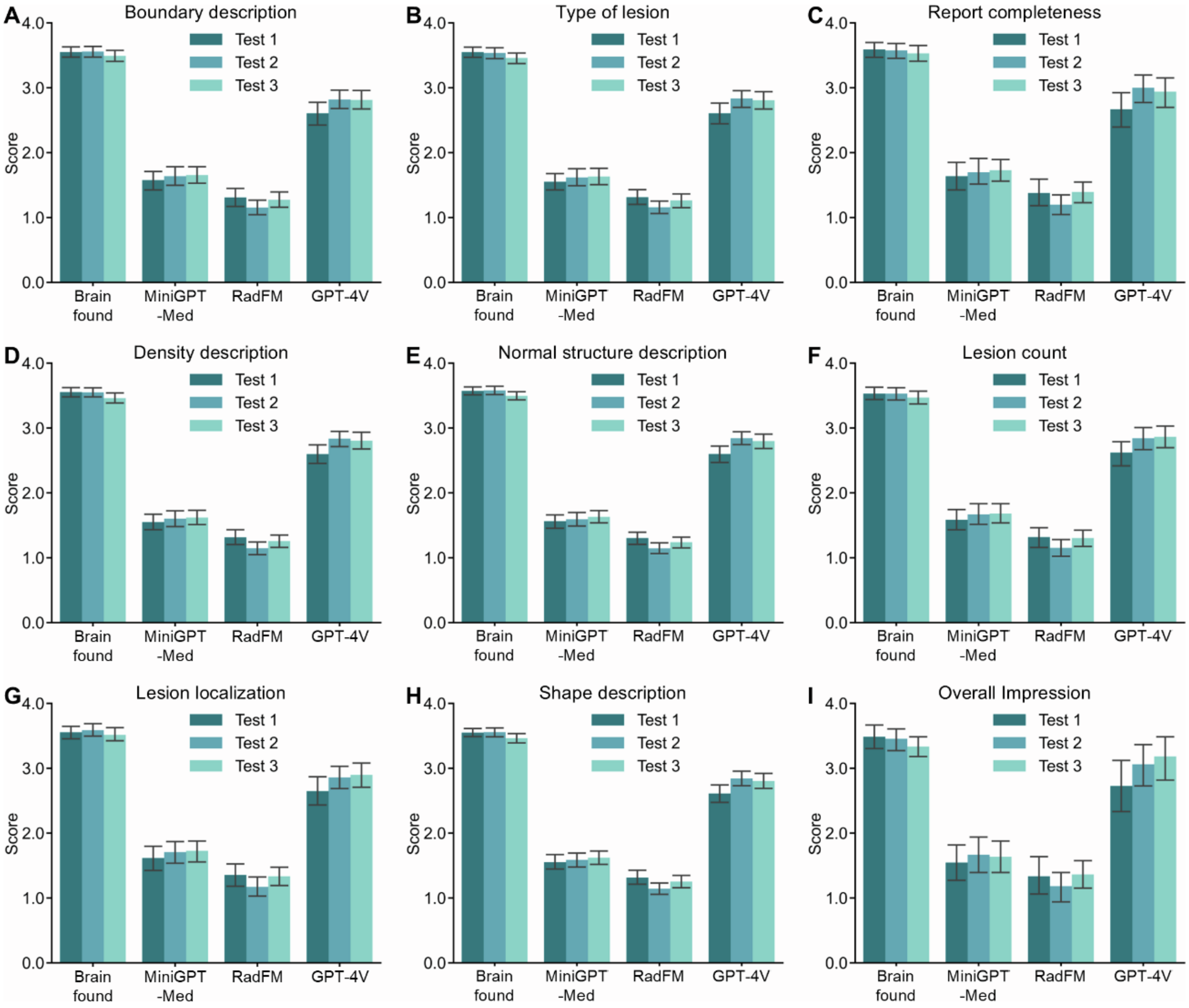

Fig. S31. GPT-4 based scoring of reports generated by four methods across nine evaluation dimensions

(A) Boundary description.

(B) Lesion type.

(C) Report completeness.

(D) Density description.

(E) Description of normal structures.

(F) Lesion count.

(G) Lesion localization.

(H) Shape description.

(I) Overall impression. The prompt for GPT-4 during the evaluation process is as follows: *You are an excellent radiologist, particularly skilled in determining whether a brain CT report is correct and compliant with standards. I will provide you with 5 reports, the first of which is written by a professional doctor after interpreting the CT. The other four reports are written by four different methods. I need you to score the other four reports based on the first report. Please score separately for the following aspects: overall*

impression, report completeness, normal structure description, lesion localization, lesion quantity, lesion type boundary description, density description, and shape description.

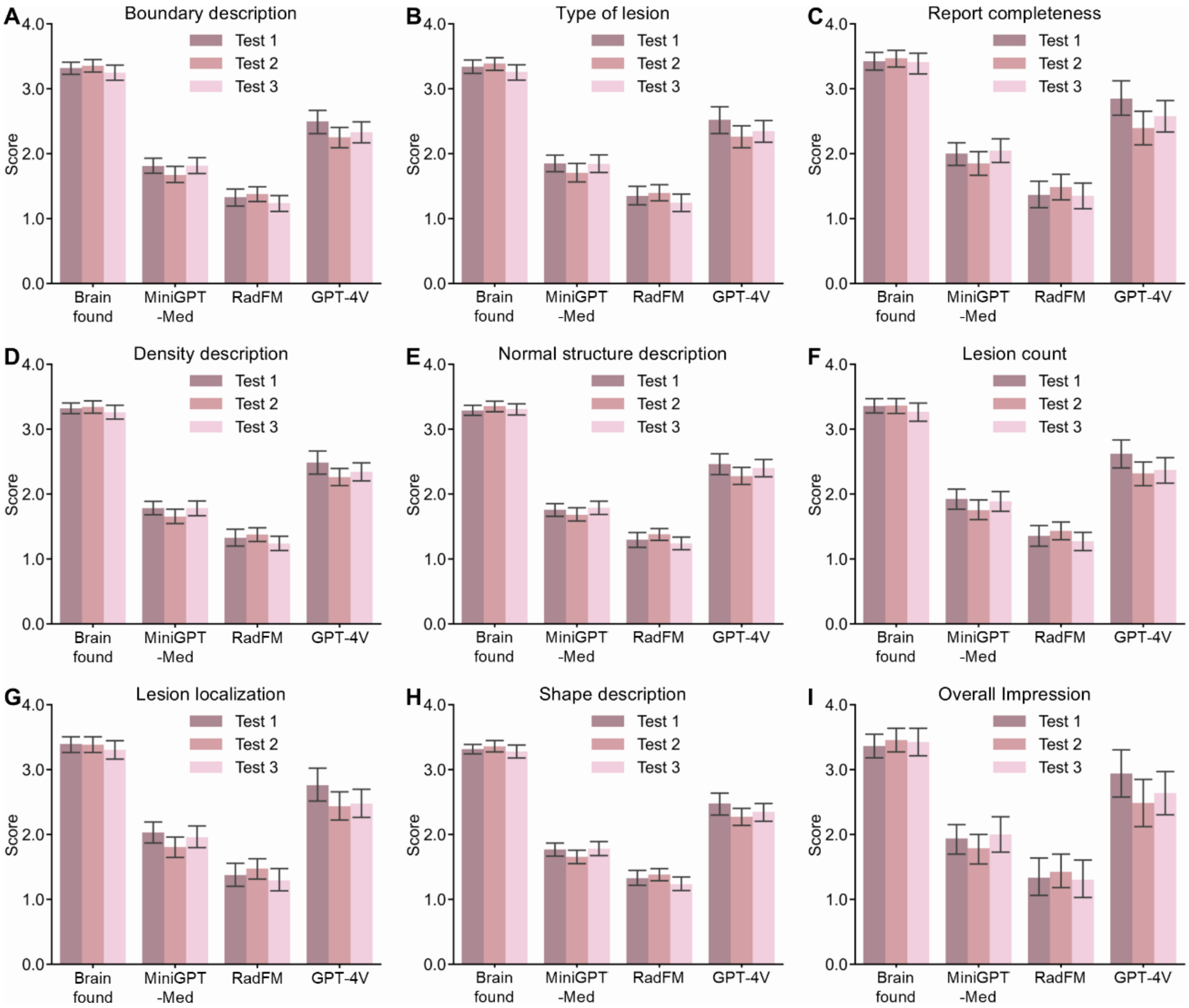

Fig. S32. GPT-4o based scoring of reports generated by four methods across nine evaluation dimensions

(A) Boundary description.

(B) Lesion type.

(C) Report completeness.

(D) Density description.

(E) Description of normal structures.

(F) Lesion count.

(G) Lesion localization.

(H) Shape description.

(I) Overall impression. The prompt for GPT-4o during the evaluation process is the same as GPT-4 in Supplementary Fig. 31.

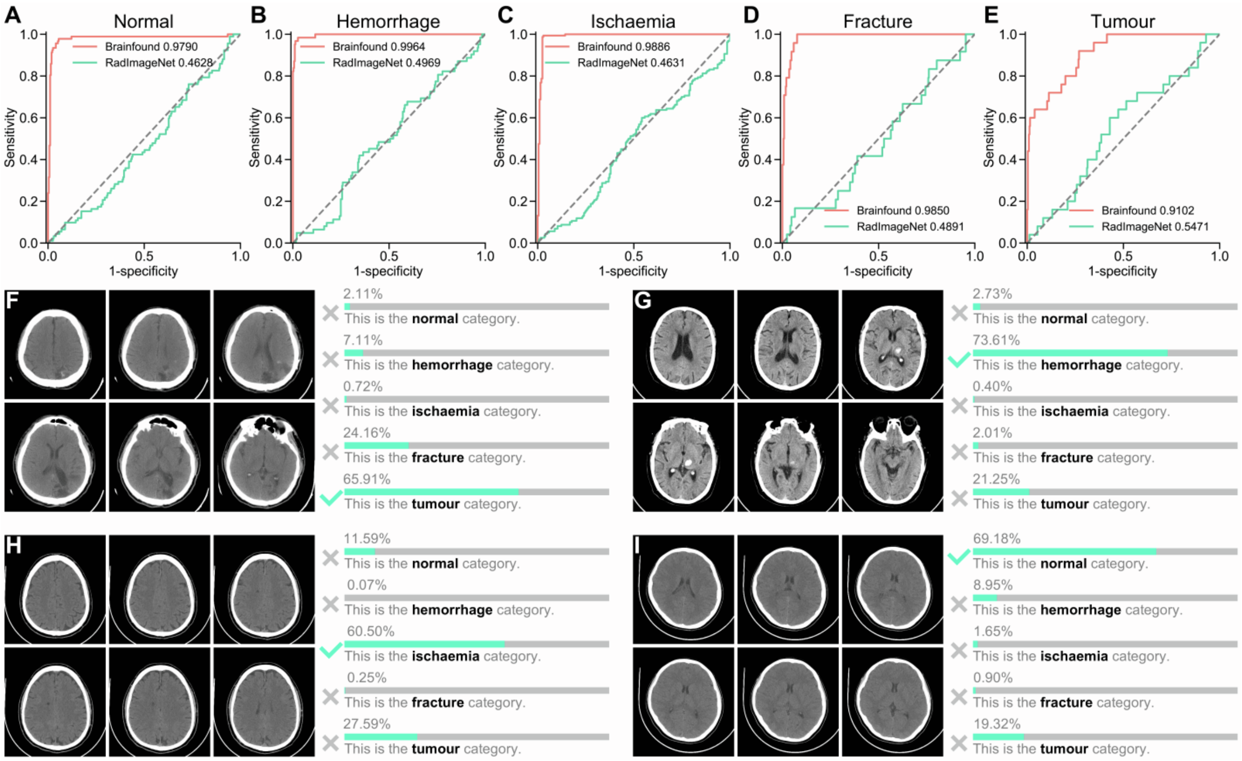

Fig. S33. Zero-shot classification performance on an external test set

(A-E) The zero-shot classification results of Brainfound, with RadImageNet serving as the comparison method on the external test set. The ROC curves, arranged from left to right, represent the categories of normal, hemorrhage, ischemia, fracture, and tumor.
(F) Predicted probability outputs for the tumor class.
(G) Predicted probability outputs for the hemorrhage class.
(H) Predicted probability outputs for the ischemia class.
(I) Predicted probability outputs for the normal class.

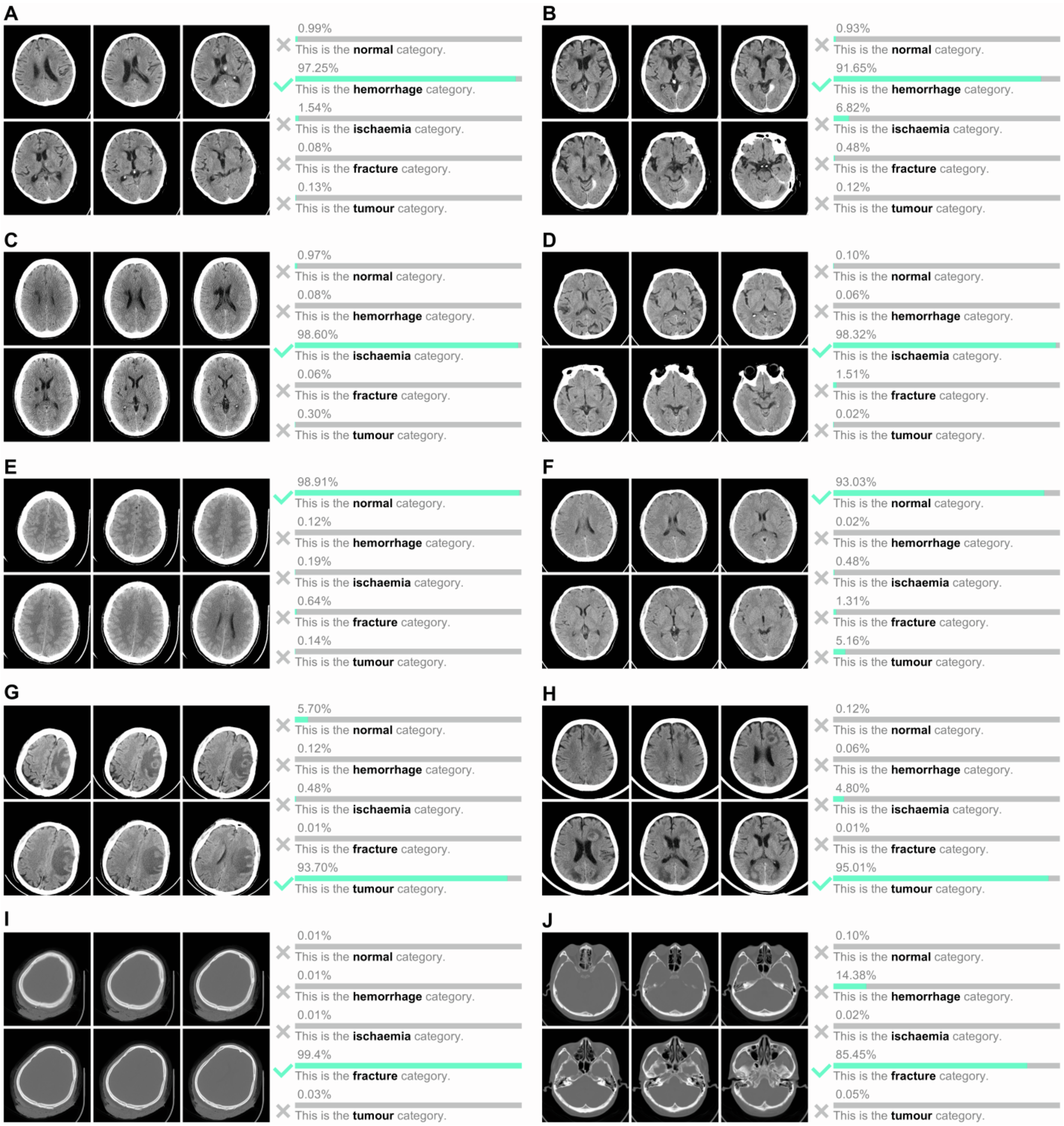

Fig. S34. Zero-shot classification examples using the aligned image encoder and text encoder in Brainfound

(A-B) Two cases for the classification of brain hemorrhage types. The output probabilities are 97.25% and 91.65%, respectively.
(B-D) Two cases for the classification of brain ischemia types. The output probabilities are 98.60% and 99.35%, respectively.
(E-F) Two cases for the classification of brain normal types. The output probabilities of 98.91% and 93.03%, respectively.
(F-H) Two cases for the classification of brain tumor types. The output probabilities are 93.70% and 95.01%, respectively.
(I-J) Two cases for the classification of brain fracture types. The output probabilities are 99.40% and 85.45%, respectively.

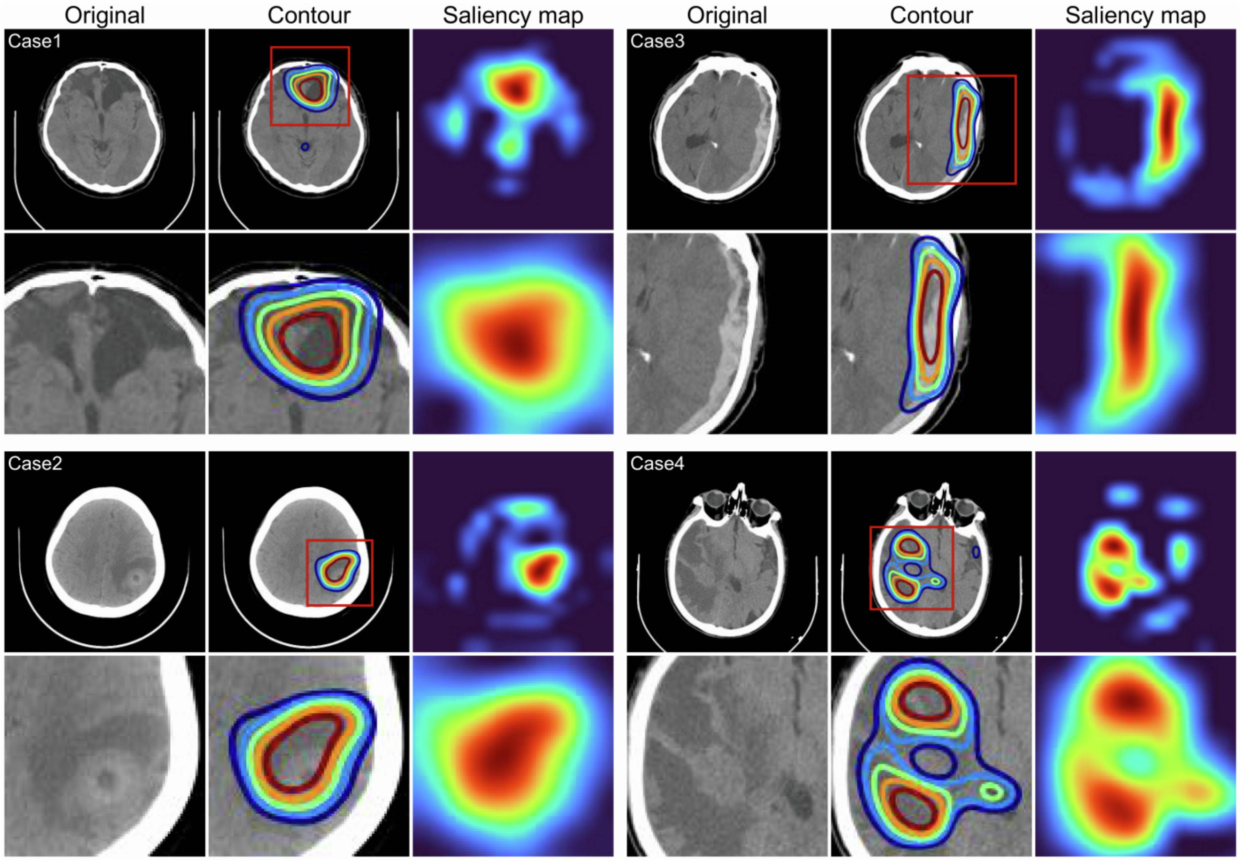

Fig. S35. Saliency maps generated by Brainfound for zero-shot classification

We showcased representative images of saliency maps for four brain CT images from Brainfound. The brain CT images are positioned in the first and fourth columns. The second and fifth columns show the saliency contours. The saliency maps developed by Brainfound are located in the third and sixth columns. The images in the second and fourth rows offer an enlarged perspective of the sections highlighted by red boxes in the first and third rows.

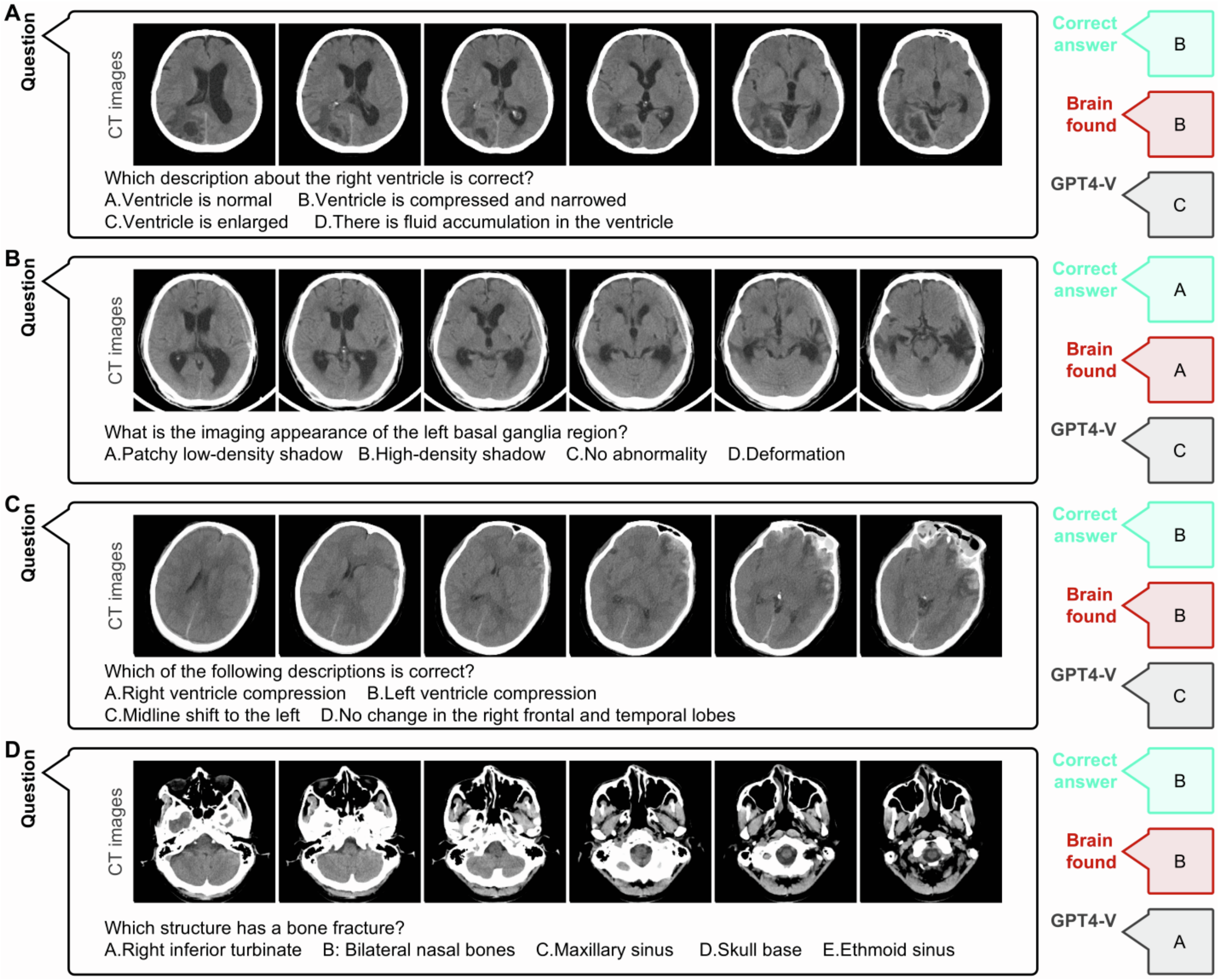

Fig. S36. The responses of Brainfound to multiple-choice questions on brain imaging, Part I

(A) With the CT images, Brainfound accurately identified the imaging characteristics of the left basal ganglia.
(B) Utilizing brain CT imaging, Brainfound determines which option is correct.
(C) Brainfound accurately selected the description of the right ventricle.
(D) Brainfound accurately identified the location of the fracture.

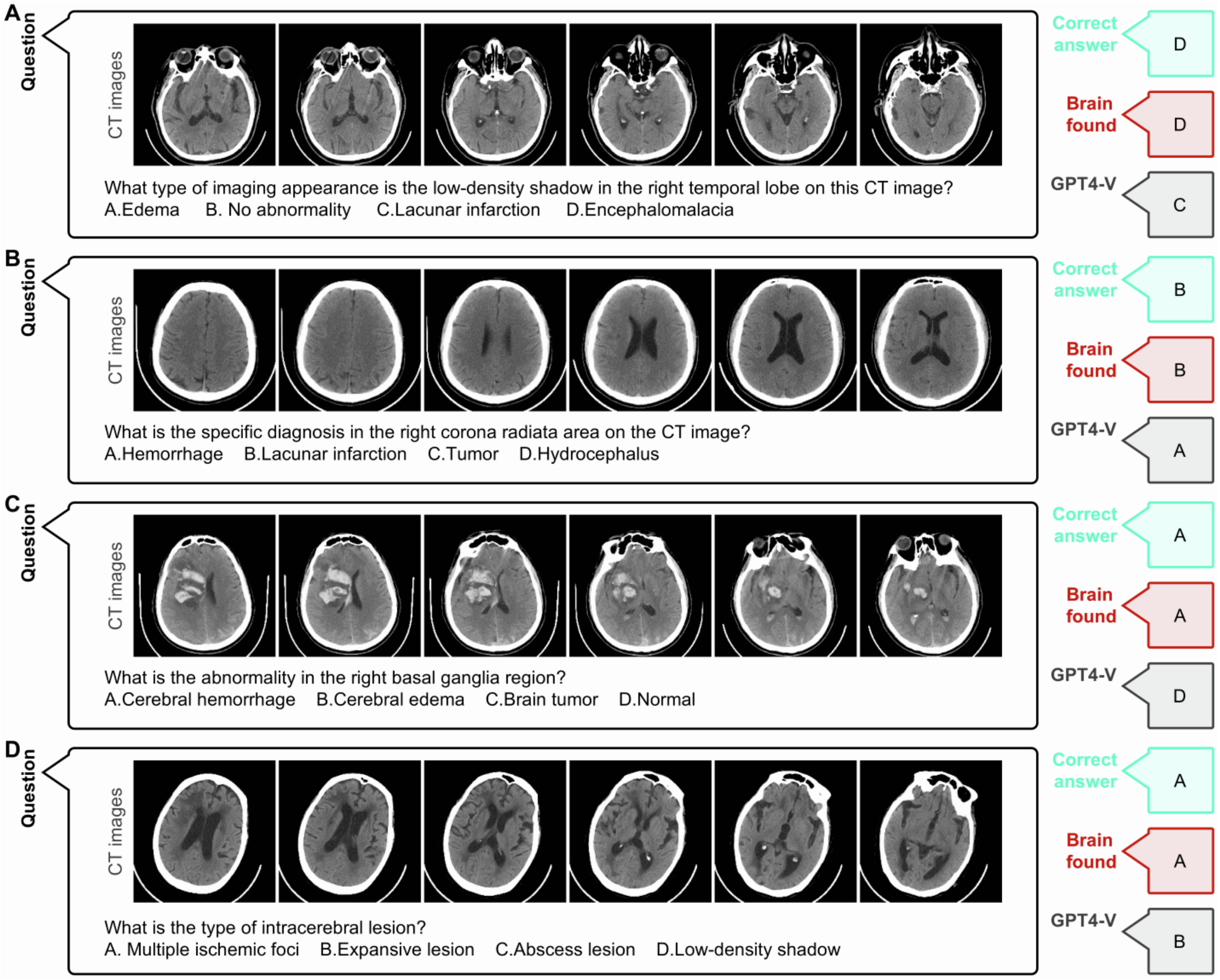

Fig. S37. The responses of Brainfound to multiple-choice questions on brain imaging, Part II

(A) Brainfound accurately determined the type of low-density shadow in the right temporal lobe depicted in the CT images.
(B) Brainfound accurately determined the specific diagnostic result for the right corona radiata area.
(C) Brainfound correctly identified the abnormality type in the right basal ganglia region.
(D) Brainfound accurately determined the type of brain lesion in the CT image.

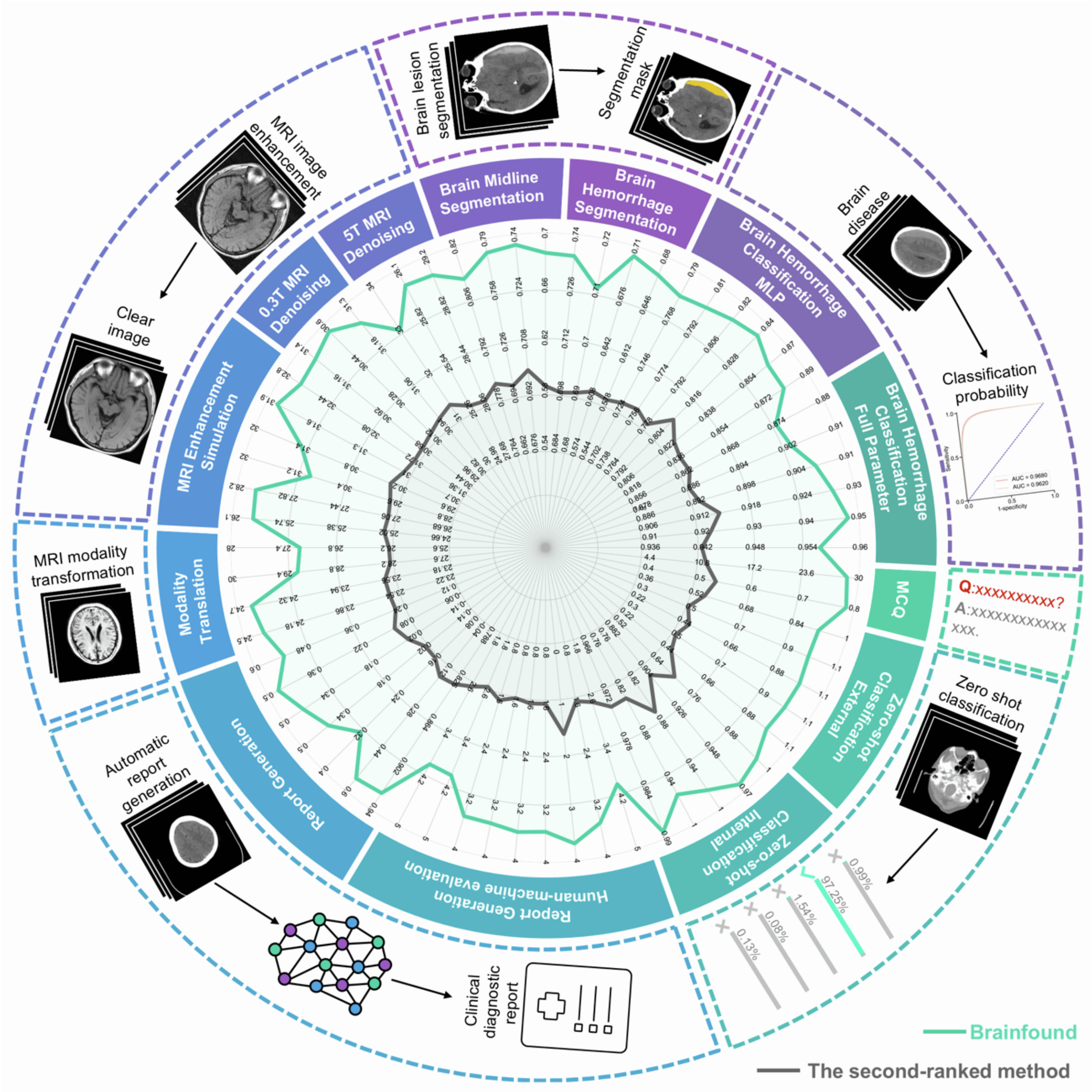

Fig. S38. Radar chart summarizing evaluation results across tasks

The radar chart displays all the experimental results. Two curves are plotted, one representing the results of Brainfound and the other representing the results of the second-ranked method in all comparisons. The names of the task types are labeled on the outside of the radar chart.

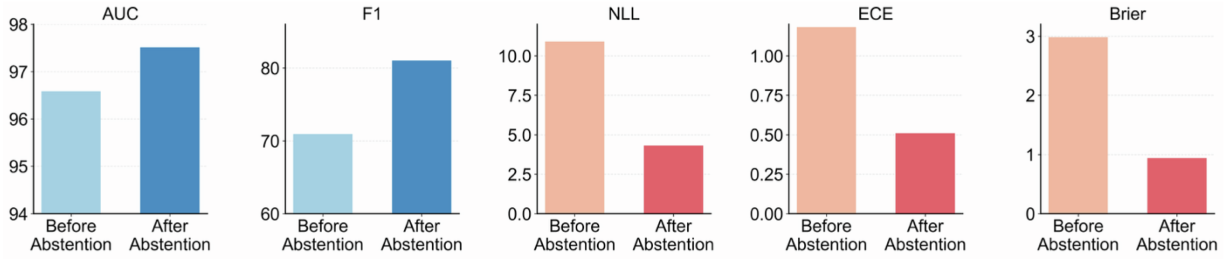

Fig. S39. Uncertainty-aware abstention improves both discrimination and calibration performance

The metrics in blue (AUC and F1) indicate better performance with higher values, whereas the metrics in red (NLL, ECE, and Brier score) indicate better performance with lower values.

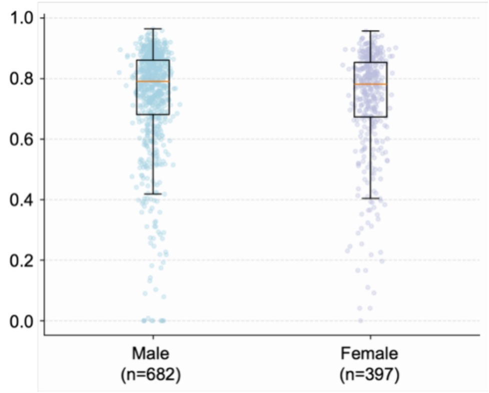

Fig. S40. Sex-based subgroup analysis of segmentation performance

Dice scores are highly comparable between male (n=682) and female (n=397) patients, with overlapping distributions and similar medians. No statistically significant difference was observed (two-sided Mann-Whitney U test, $p = 0.297$; negligible effect size).

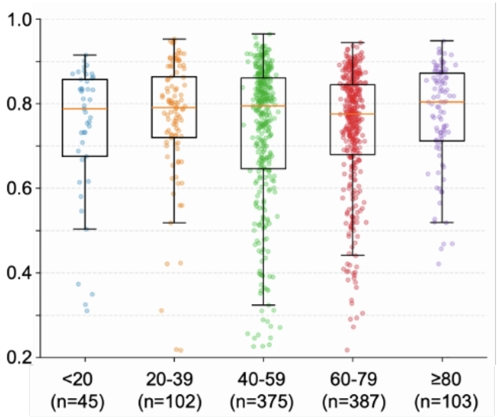

Fig. S41. Age-based subgroup analysis of segmentation performance.

Dice scores are stable across five age groups (<20 to ⩾80 years), with overlapping distributions and no monotonic age-related trend. No significant differences were observed (Kruskal–Wallis p = 0.323), and Dice showed no correlation with age, indicating no meaningful age-related performance bias.

Class	τ	Coverage (finetune set)	Coverage (non-abstained)
1	0.1873	0.9996	0.9995
2	0.0454	0.9448	0.9443
3	0.0201	0.9003	0.8994
4	0.1411	0.9957	0.9959
5	0.0322	0.7486	0.7471
6	0.0149	0.3415	0.3418

Table S1. The class-specific selection thresholds (τ) and the corresponding uncertainty coverage on the fine-tuning set and the non-abstained subset

Movie S1. The demo of Brainfound on free conversation around brain CT images

Two cases are presented: case 1 discusses what a cerebral infarction is and what a high-density linear signal shadow is. Case 2 is about cerebral hemorrhage, discussing what cerebral hemorrhage is and its effects on other areas
