# Supplementary material for: A multi-modal foundation model for brain disease diagnosis and medical imaging
Source: Patterns (N Y). 2026 Apr 14;7(6):101538. doi: 10.1016/j.patter.2026.101538 (PMC13280722; doi:10.1016/j.patter.2026.101538)
Supplement: Document S2. Article plus supplemental information [file mmc3.pdf]

Patterns

Article

A multi-modal foundation model for brain disease diagnosis and medical imaging

Highlights

- Brainfound is a multi-modal foundation model for brain CT and MRI analysis
- Brainfound integrates diffusion-based generation with image-text contrastive learning
- Brainfound spans tasks from low-level imaging to diagnosis, reports, and dialogue

Authors

Guoxun Zhang, Zebin Gao, Caohui Duan, ..., Yuchen Guo, Xin Lou, Qionghai Dai

Correspondence

feng-xu@tsinghua.edu.cn (F.X.),
yuchen.w.guo@gmail.com (Y.G.),
louxin@301hospital.com.cn (X.L.),
qhdai@tsinghua.edu.cn (Q.D.)

In brief

Accurate diagnosis of brain disorders requires combining medical images with clinical knowledge. Brainfound is a large multi-modal AI model trained on millions of brain CT and MRI images paired with reports. By integrating image understanding, text reasoning, and image generation, Brainfound supports diagnosis, report writing, and clinical dialogue, demonstrating how foundation models can advance human-in-the-loop brain health care.

Zhang et al., 2026, Patterns 7, 101538
June 12, 2026 © 2026 The Authors. Published by Elsevier Inc.
https://doi.org/10.1016/j.patter.2026.101538

CellPress

Patterns

CellPress
OPEN ACCESS

Article

A multi-modal foundation model for brain disease diagnosis and medical imaging

Guoxun Zhang,[1,2,12] Zebin Gao,[2,4,12] Caohui Duan,[5,12] Jiaxin Liu,[6] Yuerong Lizhu,[8,9] Yaou Liu,[8,9] Qian Chen,[7] Ling Wang,[7] Kailun Fei,[10] Tianyun Wang,[4] YuJia Chen,[6] Yanchen Guo,[6] Feng Xu,[3,*] Yuchen Guo,[2,13,*] Xin Lou,[5,*] and Qionghai Dai[1,2,11,*]
[1]Department of Automation, BNRist, Tsinghua University, Beijing 100084, China
[2]Beijing National Research Center for Information Science and Technology, Tsinghua University, Beijing 100084, China
[3]School of Software, BNRist, Tsinghua University, Beijing 100084, China
[4]School of Information Science and Technology, Fudan University, Shanghai 200438, China
[5]Department of Radiology, Chinese PLA General Hospital, Beijing 100039, China
[6]Tsinghua Shenzhen International Graduate School, Tsinghua University, Shenzhen 518071, China
[7]Beijing Friendship Hospital, Capital Medical University, Beijing 100050, China
[8]Department of Radiology, Beijing Tiantan Hospital, Capital Medical University, Beijing 100070, China
[9]Tiantan Image Research Center, China National Clinical Research Center for Neurological Diseases, Beijing 100070, China
[10]Cancer Hospital, Chinese Academy of Medical Sciences and Peking Union Medical College, Beijing 100730, China
[11]Senior author
[12]These authors contributed equally
[13]Lead contact
*Correspondence: feng-xu@tsinghua.edu.cn (F.X.), yuchen.w.guo@gmail.com (Y.G.), louxin@301hospital.com.cn (X.L.), qhdai@tsinghua.edu.cn (Q.D.)
https://doi.org/10.1016/j.patter.2026.101538

THE BIGGER PICTURE Brain disorders place a growing burden on global health-care systems, and neuroimaging plays a central role in their diagnosis and management. Despite rapid progress in AI, most imaging algorithms are developed for narrowly defined tasks and cannot easily generalize across modalities, diseases, or clinical questions, limiting their impact in real clinical settings. This study introduces Brainfound, a multi-modal foundation model designed to address this gap by learning shared representations across brain CT, MRI, and clinical language. Rather than optimizing for a single task, Brainfound is trained to support a broad spectrum of imaging functions, ranging from low-level image enhancement to high-level report generation and interactive clinical conversation, allowing the same model to adapt to diverse clinical scenarios, including limited-data and zero-shot settings.

By combining generative image modeling with language-based understanding, Brainfound illustrates how foundation models can move medical imaging beyond isolated benchmarks toward more integrated clinical support systems. Such systems have the potential to reduce repetitive workload, improve consistency in interpretation, and expand access to expert-level imaging analysis. As clinical data continue to grow in scale and complexity, multi-modal foundation models may become a key enabling technology for more scalable, interpretable, and human-centered brain health care.

SUMMARY

The precise and comprehensive diagnosis of complex brain disorders relies on non-invasive computed tomography (CT) and magnetic resonance imaging (MRI) in conjunction with multi-modal clinical information. Here, we present Brainfound, a multi-modal foundation model for brain medical imaging that integrates image-text contrastive learning with a diffusion-based generative framework. The model was pre-trained on more than 3 million brain CT slices and 7 million brain MRI slices paired with clinical reports. In multi-center evaluations, Brainfound demonstrates state-of-the-art performance across seven tasks, including brain disease diagnosis, lesion segmentation, MRI enhancement, cross-modality translation, automatic report generation, zero-shot disease classification, and human-AI dialogue. It substantially outperforms leading models in automated report generation and clinical question answering for brain imaging, and its performance approaches that of expert physicians. These findings highlight the

This is an open access article under the CC BY license (http://creativecommons.org/licenses/by/4.0/).

potential of Brainfound for accelerating diagnosis, support treatment decisions, and advance human-in-the-loop brain health care.

INTRODUCTION

Brain computed tomography (CT) and magnetic resonance imaging (MRI) are essential non-invasive diagnostic tools in clinical practice. These radiological studies are critical for the accurate diagnosis of a wide range of brain disorders, including tumors, strokes, and neurodegenerative diseases.[1–5] CT provides rapid assessment in acute settings, particularly for detecting hemorrhagic stroke, whereas MRI offers superior soft-tissue contrast that facilitates earlier detection of brain tumors and neurodegenerative changes. Together, these modalities play a central role in ensuring timely diagnosis and guiding patient management.[6–8] Artificial intelligence (AI) is increasingly being applied to brain CT and MRI, offering advances in both image analysis and clinical decision support.[5,9] AI technologies enable automated image analysis to rapidly identify abnormalities such as lesions, tumors, and hemorrhages, thereby significantly enhancing diagnostic efficiency.[10–13] AI models improve diagnostic accuracy by recognizing complex image features and, in some settings, achieve performance comparable to that of expert radiologists.[14,15] Beyond image interpretation, AI can integrate clinical and imaging data to support personalized treatment planning and risk prediction.[16–18] These applications highlight the growing role of AI in enhancing diagnostic accuracy and informing patient management in neuroimaging.[19]

Limited labeled data and the difficulty of obtaining multi-modal annotations remain key obstacles to developing robust AI models.[20] In medical imaging, the scarcity of high-quality labeled data limits effective training and constrains model performance.[21,22] Annotating multi-modal data, such as paired brain CT and MRI, requires specialized expertise and is labor intensive and resource intensive. This process increases the risk of annotation errors and constrains dataset scale and diversity, thereby reducing model generalizability to unseen data. Furthermore, data imbalance, in which common diseases overshadow rare diseases, exacerbates overfitting and weakens overall robustness. To address these challenges, innovative strategies such as semi-supervised learning and transfer learning have been explored to improve training efficiency and model performance under limited data limitations.[23,24] Such strategies are essential to ensure that AI applications in clinical practice remain effective and reliable.

Large AI models are increasingly being explored as a potential solution to these challenges. Building on advances in large language and vision models like ChatGPT,[18,25] CLIP,[26] SimCLR,[27] and DINO,[28] analogous medical foundation models have been developed to address data scarcity and annotation barriers. These models are rapidly advancing with applications in computational pathology,[29] ophthalmic disease diagnosis,[30] ultrasonography,[31] and cancer biomarker innovation.[2] These models improve diagnostic accuracy, support knowledge transfer, and contribute to medical education.[32] Through large-scale pre-training on diverse unlabeled datasets, they learn generalizable feature representations, enabling effective performance even with limited labeled data.[7,29] Moreover, their ability to integrate multi-modal information increases their potential utility in clinical applications. While some models have been developed for general-purpose medical applications, their utility in diverse clinical scenarios of brain disease diagnosis remains limited. A major reason is the absence of multi-modal generation capabilities (e.g., image synthesis), which restricts AI from supporting the full clinical workflow, from imaging acquisition and analysis to clinical consultation and decision support.

To address these challenges, we introduce Brainfound, a multi-modal AI foundation model for brain disease diagnosis, built on a diffusion-based generative framework and image-text contrastive learning (Figure 1). Brainfound was pre-trained on two large-scale institutional datasets, BrainCT-3M and BrainMRI-7M (Figures S1 and S2). BrainCT-3M contains over 3 million CT slices, corresponding to 107,754 brain CT scans with paired diagnostic reports. BrainMRI-7M contains over 7 million MRI slices, corresponding to 68,653 multi-sequence brain MRI scans with paired diagnostic reports. By leveraging these datasets, we have pre-trained image encoders and decoders based on the diffusion-based framework (Figure 1B), as well as text decoders based on the phrase-level masking strategy[33] (Figure 1B). We aligned the visual module and the language module of Brainfound by contrastive learning. These designs enable Brainfound to generalize across seven representative tasks, including diagnosis, lesion segmentation, MRI enhancement, cross-modality translation, report generation, zero-shot disease classification, and human-AI dialogue. In multi-center evaluations spanning public, private, and international cohorts, Brainfound consistently outperformed existing multi-modal models. Brainfound achieved higher accuracy in automatic report generation, with nearly a 50% gain over the leading baseline in human-machine evaluation and in multiple-choice brain imaging questions, where it improved accuracy by 48%, approaching expert physician performance.

RESULTS

Brainfound serves as a multi-modal AI foundation model for brain disease diagnosis

Large-scale datasets are essential for building robust AI models. We assembled a comprehensive national clinical brain imaging dataset containing BrainCT-3M and BrainMRI-7M collected from the Chinese PLA General Hospital. To construct the pre-training data for brain CT, we first collected 630,992 scans acquired between 2008 and 2022 (58% male, 42% female; Figure S1). We then filtered the dataset based on image quality, particularly signal-to-noise ratio (SNR), as well as the diagnostic richness of the accompanying reports (methods). After filtering, we retained 105,184 CT scans (from 59,935 males and 45,249 females) with paired diagnostic reports. This filtered dataset includes approximately 46,066 cases of normal individuals, 25,197 cases of ischemia, 20,798 cases of hemorrhage, 19,497 cases of fractures, and 3,282 cases of tumors. These

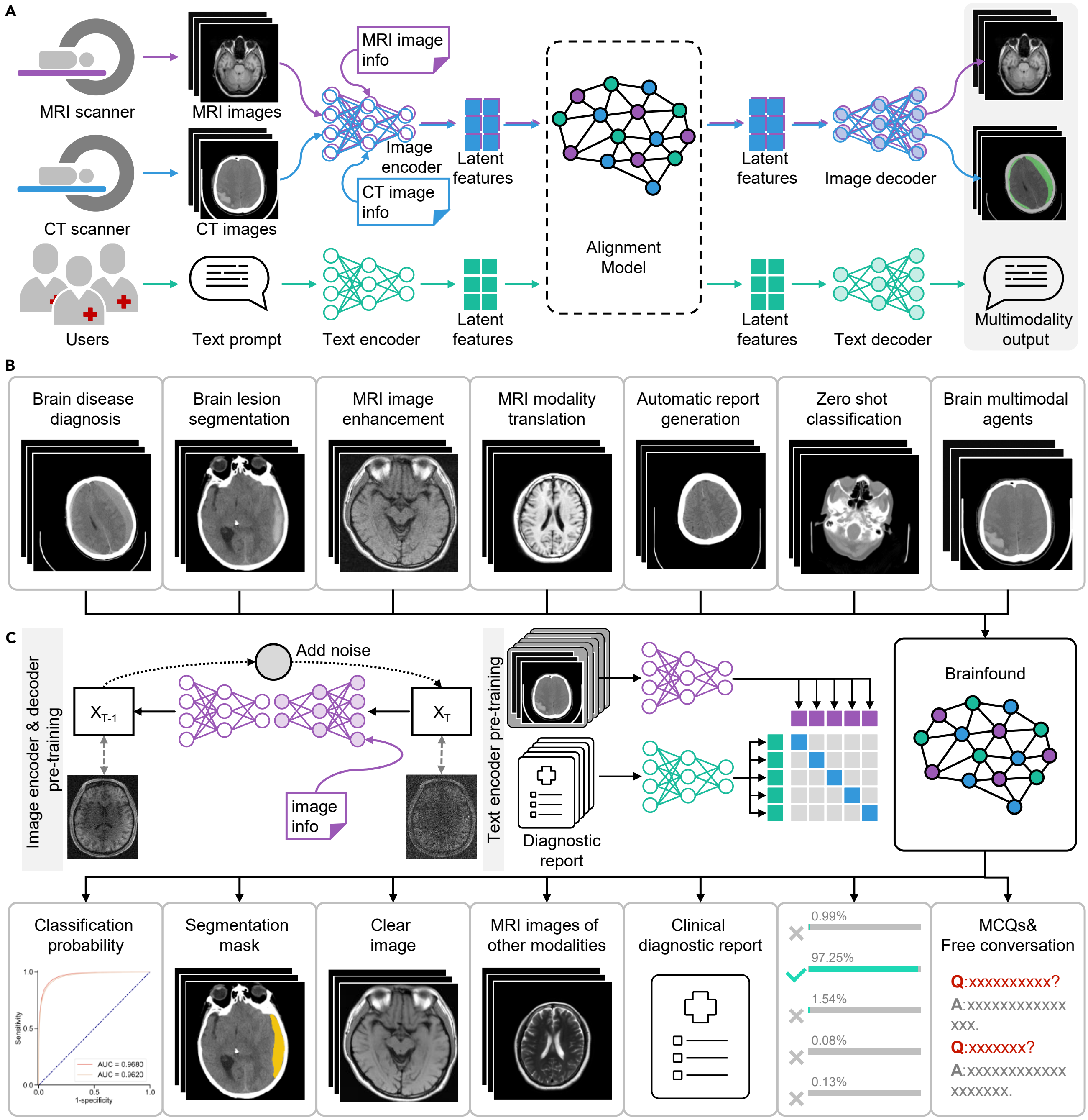

Figure 1. Overview of Brainfound

(A) Brainfound ingests CT or MRI scan sequences together with image metadata and free-text instructions. An image encoder maps images to a latent space, and a text encoder maps instructions to another latent space. An alignment module projects both into a shared representation space, after which two decoders generate multi-modal outputs, including enhanced or translated images, segmentation masks, and natural language reports or dialogue.

(B) Downstream task evaluation for Brainfound. As a foundation model, Brainfound demonstrates strong performance across a diverse set of downstream tasks, ranging from pixel-level image processing to multi-modal clinical reasoning. These include brain lesion segmentation, disease diagnosis and zero-shot classification, and MRI image enhancement and cross-modality translation as well as higher-level tasks such as automated radiology report generation and open-ended clinical dialogue. Together, these evaluations highlight the capacity of Brainfound to bridge low-level image analysis with high-level diagnostic interaction.

(C) Stepwise pre-training strategy for Brainfound. The image encoder and decoder of Brainfound are pre-trained within a diffusion framework using the large-scale BrainCT-3M and BrainMRI-7M datasets. In parallel, the text encoder is pre-trained via contrastive learning to align diagnostic reports with their corresponding image sequences in the latent space. This modular design enables Brainfound to integrate visual and textual representations effectively.

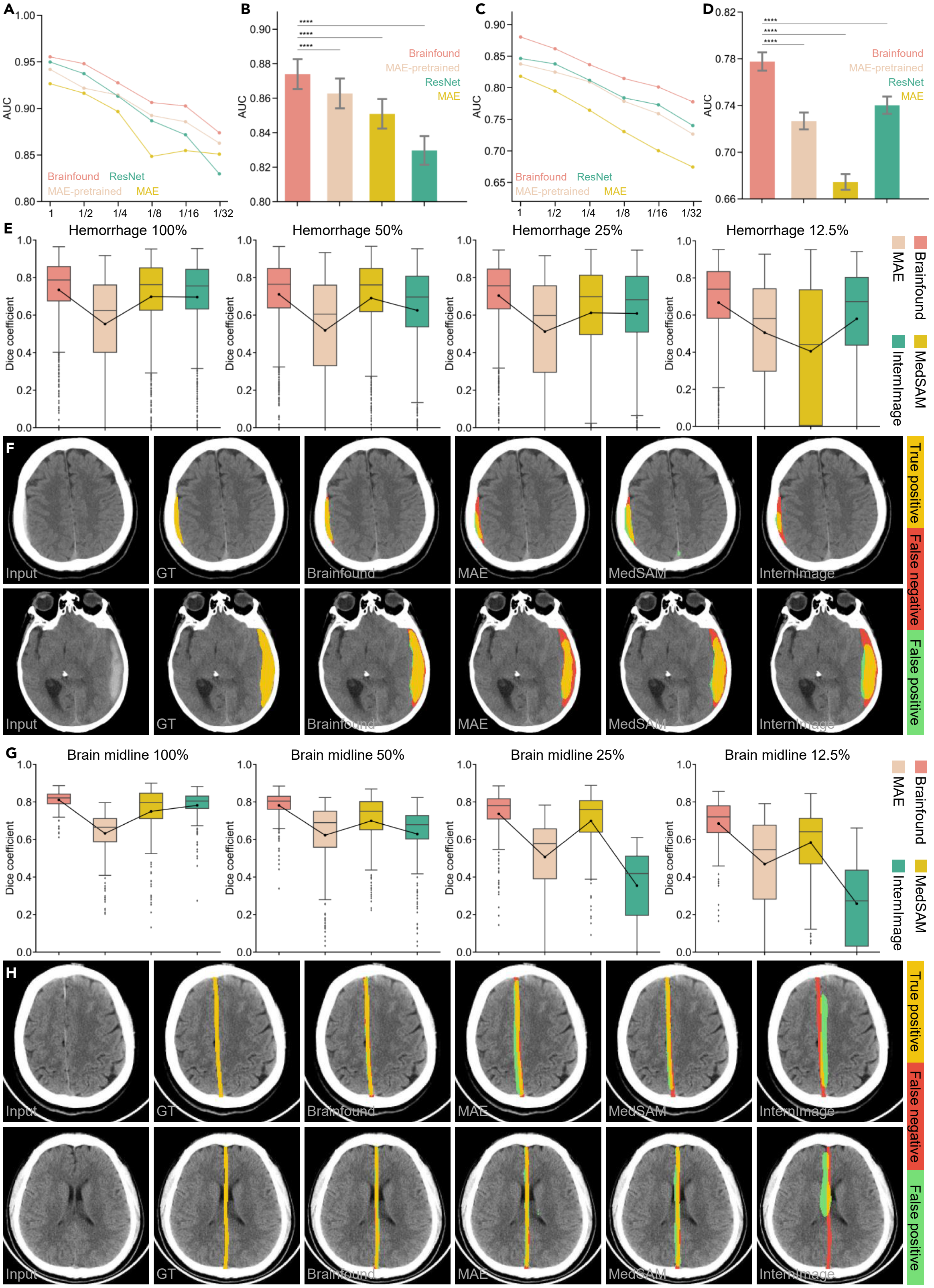

(legend on next page)

CellPress
OPEN ACCESS

CT scans comprise over 3 million 2D slices, forming the BrainCT-3M dataset. Additionally, we collected 68,653 brain MRI scans with paired diagnostic reports, including scans from 36,002 males and 32,651 females, with admission dates ranging from 2018 to 2023 and ages spanning 1 to 105 years (Figure S2). The dataset covers multiple major MRI sequences, including T1-weighted imaging (T1WI), T2-weighted imaging (T2WI), diffusion-weighted imaging (DWI; low b value and standard b value), and fluid-attenuated inversion recovery (FLAIR), among others. In total, these scans comprise over 7 million MRI slices, forming the BrainMRI-7M dataset. We employed the GPT-4 API (application programming interface) to automatically parse and analyze MRI reports, allowing us to count the scan types and quantities involved in BrainMRI-7M. Automated report analysis revealed that ischemia was the most frequently mentioned term, followed by softening focus, inflammation, and other vascular and degenerative conditions (Figure S2). Meanwhile, we curated datasets for downstream validation from both public benchmarks and datasets from our institution and collaborating centers. The publicly available datasets include the RSNA Intracranial Hemorrhage Classification dataset[34] for validating classification tasks and the BraTS MRI1 dataset for image quality enhancement. In-house clinical data include physician-annotated intracranial hemorrhage segmentation data, midline shift segmentation data, multiple-choice question (MCQ) data, report generation data, zero-shot classification data from both internal and external centers, and MRI data for modality conversion.

In the training phase of the Brainfound vision module (Brainfound-v), we utilized a U-Net architecture augmented by transformer blocks with cross-attention[35] mechanisms, comprising approximately 78 million trainable parameters (Figure S3; methods). Paired clinical metadata were encoded using a text encoder[36] and then integrated with corresponding brain images to serve as input for the primary network architecture of Brainfound-v. The self-supervised pre-training of Brainfound-v was conducted on the diffusion-based framework[37,38] (methods). A 2D Brain CT or MRI image, along with its basic information, was randomly selected from the BrainCT-3M and BrainMRI-7M datasets and subjected to data augmentation. In the course of forward propagation, Gaussian noise with a defined intensity was systematically introduced into the image. Upon reaching 1,000 iterations, this procedure culminated in the conversion of the image into pure noise (Figures 1B and S4A). Throughout backward propagation, the neural network was trained to denoise and reconstruct clean images (Figure S5), thereby enabling robust representation learning (Figure S6). In the report generation stage, we adopted ChineseBERT (102M trainable parameters) as the text processor to enable efficient text processing and better adaptation to Chinese radiology reports. The text encoder was further trained on our diagnostic reports (Figure S7; methods). For the task of open-ended conversation, we fine-tuned Brainfound with our instruction datasets automatically generated from BrainCT-3M and BrainMRI-7M by GPT (Figure S8; methods).

During inference, task-specific adapters were introduced to leverage the capabilities of Brainfound. For the cerebral hemorrhage classification task, a trainable multi-layer perceptron (MLP) was utilized to map the final output features of the Brainfound image encoder into diagnostic labels (Figure S4B; methods). In the segmentation tasks for cerebral hemorrhage and midline shift, multiple learnable MLP classifiers were applied to intermediate feature maps for pixel-wise classification (Figure S4C; methods). For the modality transfer task, the diffusion model was fine-tuned by conditioning the image encoder-decoder on modality-specific inputs. In the denoising task, we implemented a zero-shot learning denoising framework based on the pre-trained image encoder-decoder.

Diagnosis and localization of brain diseases with Brainfound

We first evaluated Brainfound on intracranial hemorrhage, a life-threatening condition that requires accurate and timely diagnosis. We compared full-parameter fine-tuning and MLP-head fine-tuning of Brainfound with other methods on the publicly available RSNA intracranial hemorrhage classification dataset. We randomly split 222,218 images into equal halves for training and testing from the RSNA intracranial hemorrhage dataset. The first experiment involved full-parameter fine-tuning of models with different amounts of training data to compare the accuracy of intracranial hemorrhage classification. We compared Brainfound, the original pre-trained MAE model (MAE),[39] MAE pre-trained on medical images (MAE pre-trained), and pre-trained RadImagenet (ResNet-based).[40] The models were fine-tuned on the full training set (about 110,000 images) as well as on progressively smaller subsets (1/2, 1/4, 1/8, 1/16, and 1/32 of the data). Brainfound consistently achieved the highest area under the curve (AUC) across six training data sizes (Figures 2A and S9A). The second experiment focused on fine-tuning only the tail MLP module with different training data volumes, with the pre-trained model serving as a feature extractor for brain CT images. Brainfound outperformed these three baselines across data sizes (Figure 2C). With only 1/32 of the training set (Figures 2D and S9B), Brainfound achieved an AUC of 0.7776 (95% CI, 0.7731–0.7817), outperforming ResNet, which obtained 0.7267 (95% CI, 0.7215–0.7317). These experiments demonstrate that Brainfound enables efficient knowledge transfer for learning brain imaging features and disease-related representations. To further assess the transferability and clinical applicability of Brainfound, we integrated it into an ensemble pipeline—the winning solution of the RSNA Brain Hemorrhage Classification challenge—which combines three

Figure 2. Evaluation of Brainfound on brain hemorrhage classification and segmentation

(A–D) AUC performance of Brainfound and three baselines (ResNet, MAE-pre-trained, MAE) on hemorrhage classification under full-parameter fine-tuning (A and B) and MLP-only fine-tuning (C and D). Training set sizes were varied from the entire dataset (110,000 images) down to 1/32. Bars show mean AUC ± s.e.m.; ****$p < 0.0001$ (two-sided paired t-test) in (B) and (D).

(E–H) Segmentation performance of Brainfound and three comparison models (MedSAM, MAE, and InternImage). (E and G) Accuracy across different training set sizes for hemorrhage (E; 220 CT scans) and midline (G; 439 images) segmentation under full-parameter fine-tuning. (F and H) Representative segmentation examples: original CT image, ground truth, and predictions from Brainfound, MedSAM, MAE, and InternImage. Box plots show median and interquartile range (IQR), with whiskers indicating 1.5 × IQR.

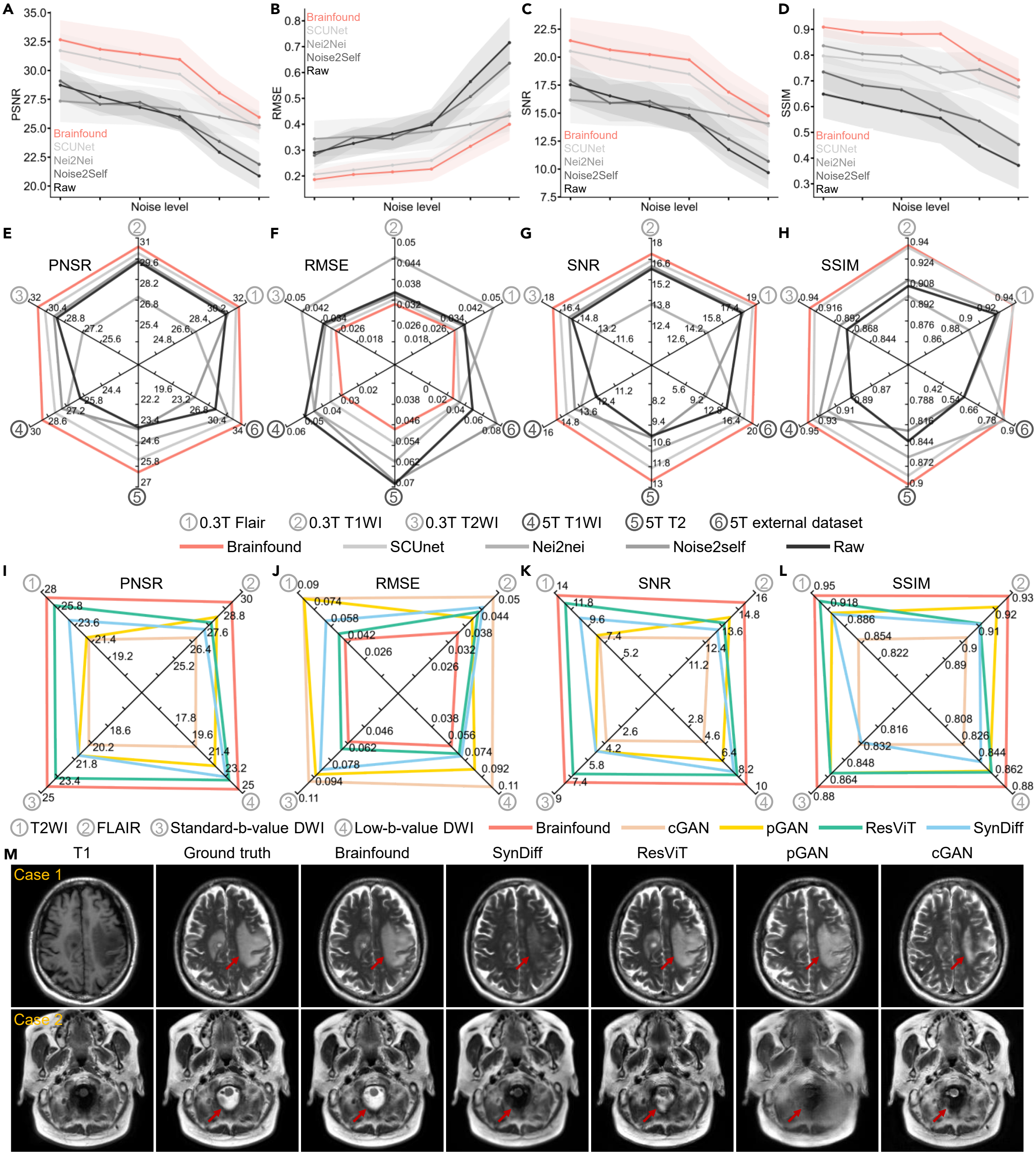

Figure 3. Evaluation of Brainfound in MRI denoising and cross-modality translation

(A–D) Zero-shot denoising on simulated data spanning six noise levels. We report PSNR, RMSE, SNR, and SSIM on the test set (n = 1,380); curves summarize Brainfound and baseline methods, with Brainfound showing consistently higher PSNR/SSIM and lower RMSE across noise conditions. Shaded regions indicate ± s.e.m.

(E–H) Zero-shot denoising on real data acquired on low-field (0.3 T) and ultra-high-field (5 T) systems. Test sets include 0.3 T FLAIR (n = 450), 0.3 T T1WI (n = 450), 0.3 T T2WI (n = 450), 5 T T1WI (n = 19), 5 T T2WI (n = 10), and an external 5 T set (n = 25). Radar charts summarize PSNR, RMSE, SNR, and SSIM per dataset; Brainfound attained top performance on most metrics.

(legend continued on next page)

heterogeneous backbones. Specifically, we replaced one backbone (DenseNet121[41]) with Brainfound, and this modification consistently achieved the highest AUC across experiments (Figures S9C and S9D). These results indicate that Brainfound can serve as an effective plug-and-play backbone within advanced ensemble strategies, providing complementary representations and further improving the performance of existing approaches.

The visual module of Brainfound was built upon a diffusion-based framework that captures essential priors for dense prediction. We evaluated its segmentation performance on intracerebral hemorrhage (ICH) and midline shift detection tasks using 2,060 CT scans from the Chinese PLA General Hospital, split into training (220 cases), validation (760 cases), and test cohorts (1,080 cases). Under this setting, the sizes of the validation and test sets were intentionally much larger than that of the training set in order to evaluate Brainfound's performance under limited training data. A larger test set better reflects real-world clinical scenarios by covering a broader spectrum of cases, including more challenging and rare examples, thereby providing a more reliable and clinically aligned assessment of the model's generalization capability. Brainfound and three comparative methods were fine-tuned with 12.5%, 25%, 50%, and 100% of the training data (Figure 2E). Across four data regimes, Brainfound consistently achieved the highest Dice scores (Figure 2E). For example, with only 12.5% of the training data, it reached 0.667 (95% CI, 0.654–0.681), substantially outperforming InternImage. Qualitatively, Brainfound also demonstrated a superior ability to detect small hemorrhages near the skull (Figure 2F). Midline shift is a clinically recognized marker of disease severity in several brain pathologies. We evaluated Brainfound on a dataset of 301 patients (methods) under few-shot learning settings using 12.5%, 25%, 50%, and 100% of the training data (Figure 2G). Compared with MedSAM,[42] MAE,[39] and InternImage,[43] Brainfound outperformed the other models, achieving the highest Dice coefficient (Figure 2G). Notably, even with only 12.5% of the training data, it achieved a Dice of 0.685 (95% CI, 0.668–0.701), substantially outperforming the second-best model (Figure 2G). Qualitatively, Brainfound generated more accurate midline segmentation masks (Figure 2H). Saliency map analysis further confirmed that its attention was concentrated on relevant hemorrhage and midline regions (Figures S10, S11, and S12).

Augmenting clinical brain imaging systems with Brainfound

MRI is a cornerstone of clinical neuroimaging, offering non-invasive, non-ionizing, and multi-parametric imaging with high soft-tissue contrast. Field strength remains a key determinant of image quality: low-field MRI is more accessible but yields lower resolution,[44–46] whereas high-field systems (e.g., 3 T and emerging 5 T) provide higher SNR and resolution but at higher cost and limited availability.[47] A persistent challenge is reducing scan time without compromising image quality. Recent AI methods address this by denoising low-SNR fast acquisitions and enabling virtual multi-modal imaging through modality transformation. Leveraging a diffusion-based framework, Brainfound demonstrated strong pixel-level performance, validated in zero-shot MRI denoising and few-shot modality translation.

We evaluated Brainfound on a zero-shot denoising task using 10 T1WI scans (1,380 images) from the BraTS 2023 dataset (3 T MRI). Such a zero-shot setting provided a stringent evaluation of the model's generalization ability, as it assesses whether the learned representations can transfer to unseen datasets and imaging conditions without any task-specific fine-tuning. Six noisy test sets were generated by adding Rician noise at different levels (methods), with average image SNRs of 9.68, 11.74, 14.79, 15.62, 16.53, and 17.54 dB. We constructed a zero-shot learning iterative denoising architecture using Brainfound (Figure S13; methods) and compared it with SCUNet,[48] Neighbor2neighbor[49] (Nei2Nei), and Noise2Self.[50] The quality of the enhanced images was assessed by peak SNR (PSNR), root-mean-square error (RMSE), SNR, and structural similarity index (SSIM) (methods). Brainfound achieved optimal scores on four metrics across six noise levels (Figures 3A–3D). For instance, at an average input SNR of $\sim$14.80 dB, Brainfound improved the output SNR to 19.76 dB, exceeding the second-best method (SCUNet, 18.5 dB) (Figure 3C). Qualitatively, Brainfound effectively removed noise while preserving fine structural details (Figure S14). We further assessed Brainfound on real MRI datasets spanning field strengths from 0.3 T to 5 T, covering multiple sequences (T1WI, T2WI, and FLAIR) (methods). The 0.3 T low-field MRI images were sourced from the M4Raw dataset, including T1WI (450 images), T2WI (450 images), and FLAIR (450 images). The 5 T ultra-high-field MRI images were collected at Beijing Friendship Hospital, including T2WI (10 images) and T1WI (19 images), and 25 images were collected using a 5 T MRI at Shanghai United Imaging as the external test set (methods). Across four metrics in the test datasets, Brainfound outperformed competing methods, with PSNR gains of up to 5% over the second-best model (Figure 3E). These results highlight Brainfound's robustness for MRI denoising in both synthetic and real-world settings (Figures S15, S16, S17, S18, S19, and S20).

We evaluated Brainfound on MRI modality transformation across five clinically common sequences (T1WI, T2WI, FLAIR, low-b-value DWI, and standard-b-value DWI) using 182 3 T brain MRI cases from the Chinese PLA General Hospital (methods). Modality absence is common in routine clinical workflows; thus, modality transformation offers a practical solution for handling incomplete MRI protocols. The task was to generate the other four modalities from T1WI, and Brainfound was compared with SynDiff,[51] ResViT,[52] pGAN,[53] and cGAN,[54] representing commonly used architectures for modality transfer. Performance was assessed using PSNR, RMSE, SNR, and SSIM. Brainfound consistently outperformed these four

(I–L) Cross-modality translation from T1WI to T2WI, FLAIR, low-b-value DWI, and standard-b-value DWI. Training used 94 head 3 T scans (2,205 images), and testing used 88 head 3 T scans (1,936 images). Radar charts report metric-wise performance across methods; Brainfound leads across metrics.

(M) Representative T1WI to T2WI translations. Case 1: the hemorrhagic lesion (red arrow) is preserved with greater fidelity in the output of Brainfound. Case 2: structures at the foramen magnum (red arrow) are more distinctly depicted, with hyperintense cerebrospinal fluid contrasted against the skull, medulla oblongata, and vertebral arteries.

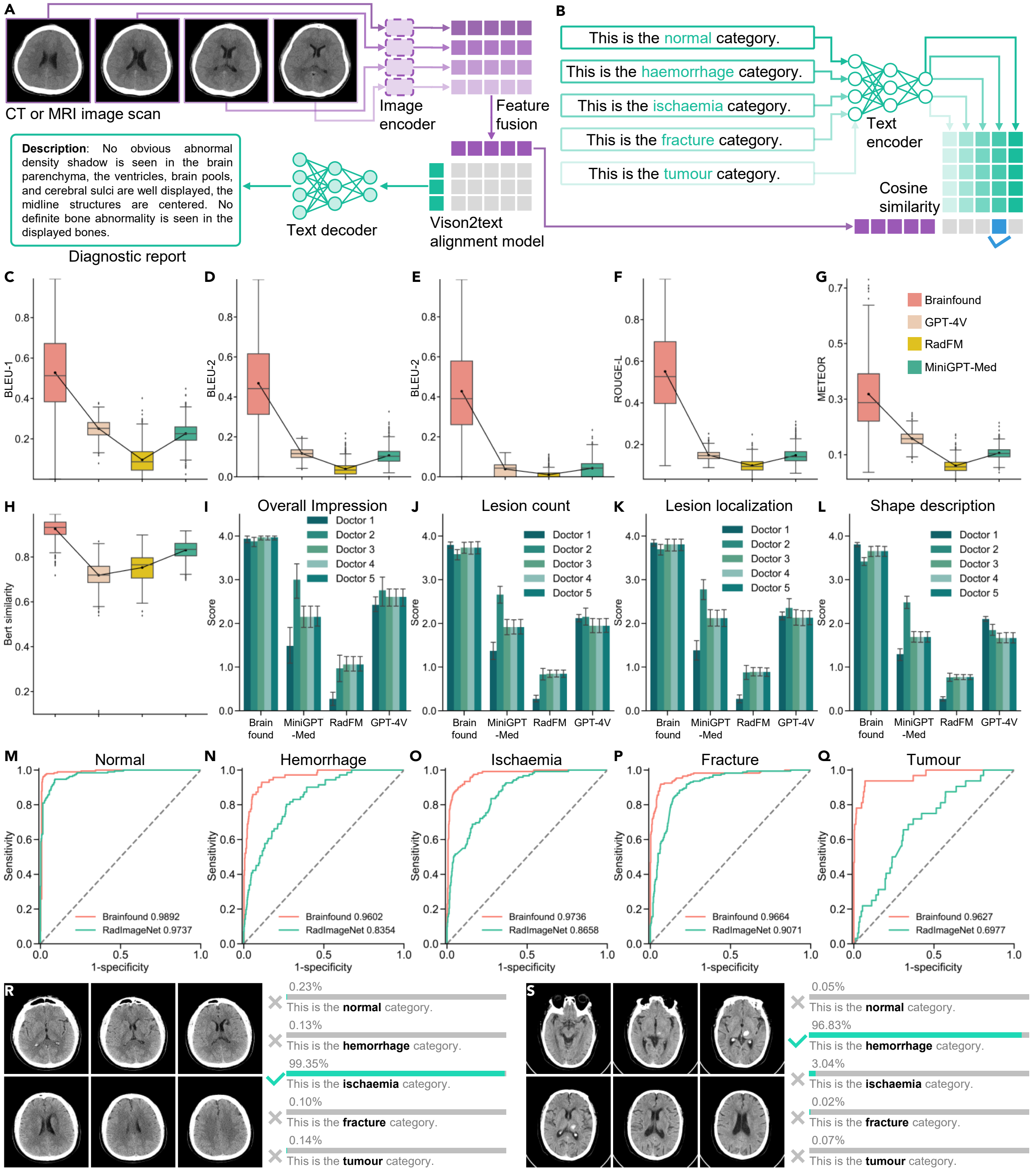

Figure 4. Evaluation of Brainfound on radiology report generation and zero-shot brain imaging classification

(A) Image sequences (CT or MRI) and paired diagnostic reports are mapped by the image and text encoders into a shared latent space. An alignment module learns cross-modal correspondence.

(B) Zero-shot classification with the alignment head. Natural-language class prompts are embedded by the text encoder and compared with image embeddings in the shared space. Class predictions are obtained by similarity-based scoring.

(C–H) Automatic report generation on the held-out test set (n = 990), comparing Brainfound with GPT-4V, RadFM, and MiniGPT-Med. Radar charts summarize BLEU-1, BLEU-2, BLEU-3, ROUGE-L, METEOR, and BERTScore (methods). Boxes indicate median and IQR, with whiskers extending to 1.5 $\times$ IQR.

(legend continued on next page)

baselines across four transformation tasks (Figures 3I–3L). In SNR comparisons (Figure 3K), Brainfound achieved notable gains over the second-best model across four modalities (e.g., ∼8% improvement for T1WI to T2WI and ∼12% for T1WI to DWI; Figure 3I). Qualitatively, Brainfound better delineated lesion regions and preserved critical anatomical structures (Figure 3J). Additional examples of modality conversion are available in Figures S21, S22, S23, and S24.

Automatic generation of clinical reports with Brainfound

Automatic report generation can improve patient care and radiologist efficiency. Using the BrainCT-3M and BrainMRI-7M datasets, we pre-trained the text encoder and decoder with a phrase-level masking strategy and aligned the image and text encoders through contrastive learning (Figure S7; methods). This framework enabled Brainfound to generate reports directly from full 3D brain CT or MRI volumes (Figure 4A; methods). Moreover, the joint alignment of image and text representations enabled zero-shot classification of scans based on text prompts.

We evaluated the capabilities of Brainfound, GPT-4V, RadFM,[55] and MiniGPT-Med[56] for the task of automatic report generation. A test set of 990 brain CT scans and corresponding radiology reports, curated by clinicians at the Chinese PLA General Hospital, was used exclusively for evaluation and was not included in pre-training. For GPT-4V, RadFM56, and MiniGPT-Med57, reports were generated using their default prompts (Figure S25). Report quality was assessed using widely adopted NLP (natural language processing) metrics, including BLEU-1, BLEU-2, BLEU-3, ROUGE-L, METEOR, and BERT similarity. Brainfound obtained the highest performance in these evaluations. For BLEU-1 (Figure 4C), Brainfound reached 0.53, more than double the score of GPT-4V, which scored 0.25. In METEOR (Figure 4F), Brainfound scored 0.32, compared with 0.16 for GPT-4V. In BERT similarity (Figure 4H), Brainfound achieved 0.93, exceeding MiniGPT-Med (0.83) by 11%. Similar improvements were observed across other metrics (Figures 4D–4H). Representative reports for normal, ischemic, and hemorrhagic CT scans are provided in Figures S26, S27, and S28. Automatic text generation metrics such as BLEU, ROUGE, METEOR, and BERT similarity cannot reliably capture the true semantic correctness of reports nor do they reflect clinical usability in real-world practice. To address this limitation, we further organized a human-in-the-loop evaluation involving experienced clinicians, who assessed the generated reports. This clinician-guided evaluation provides a more trustworthy assessment of Brainfound. We randomly selected 33 cases from the results to form a test set and established a human evaluation framework to assess the accuracy of reports generated by four methods (methods). Using 3D Slicer, we developed an expert scoring framework for report evaluation (Figure S29). Five radiologists from three hospitals, with an average practice duration of 6.4 years (5, 3, 2, 5, and 17 years, respectively), independently assessed the reports. Each report was evaluated on nine clinically relevant criteria derived from guidelines, including overall impression and completeness as well as lesion description (count, localization, morphology, boundary, density, and type) and normal structure. For the overall impression (Figure 4I), Brainfound scored 3.95 (95% CI, 3.91–3.98), compared with 2.60 for GPT-4V. For the lesion count (Figure 4J), Brainfound scored 3.48, surpassing 1.54 for GPT-4V. Similar trends were observed for lesion localization and shape description (Figures 4J–4L). Detailed scoring results are available in Figure S30. To further assess alignment with medical knowledge, we asked GPT-4 and GPT-4o to evaluate the generated reports against clinician-written references. Both models produced scores closely matching those of human experts, with stable results across three repeated evaluations (Figures S31 and S32).

After alignment of the image and text encoders through contrastive learning, zero-shot classification was performed using text tokens (Figure 4B). To evaluate performance, we curated internal and external test sets covering five diagnostic categories: normal brain CT, cerebral hemorrhage, cerebral ischemia, skull fracture, and brain tumor. The internal set consisted of 588 scans from the Chinese PLA General Hospital, and the external set included 363 scans from Brain Hospital of Hunan Province. Compared with RadImageNet, Brainfound consistently achieved higher AUC scores across five categories in both internal and external datasets (Figures 4M–4Q and S33). For example, Brainfound reached 0.9892 versus 0.9737 for normal CT, 0.9602 versus 0.8354 for hemorrhage, and 0.9627 versus 0.6977 for tumor, where the largest performance gap was observed. Probability distribution visualizations for ischemia and hemorrhage show that Brainfound assigned higher confidence to the correct labels (Figures 4M, 4N, and S34). On the external dataset, the advantage was even more pronounced, with Brainfound achieving nearly double the AUC of RadImageNet (Figure S33). Saliency map analyses further confirmed that the attention of Brainfound was concentrated on clinically relevant regions determining image category (Figure S35).

Brainfound supports medical MCQs and open-ended clinical dialogue

Finally, to evaluate Brainfound as an AI assistant for clinical brain disease diagnosis, we designed more diverse and cognitively demanding tasks. Using image sequences and diagnostic reports from BrainCT-3M and BrainMRI-7M, together with the text understanding capacity of GPT, we constructed an instruction dataset named BrainInstru-1M, comprising 1,003,732 cases (Figure 4A). BrainInstru-1M covers two task formats: (1) MCQs, where each entry contains a brain CT or MRI sequence, a question stem with options, and the correct answer, and (2) open-ended clinical dialogue, where each entry includes a scan paired

(I–L) Expert evaluation of generated reports under a pre-defined clinical rubric on 33 blinded cases (n = 33). Five experienced clinicians scored overall quality, lesion count, lesion location, and lesion morphology (higher scores are better). Additional results are provided in Figure S30. Bar plots show mean scores, with error bars indicating mean ± s.e.m. across clinicians.

(M–Q) Zero-shot classification AUC for five categories: normal, hemorrhage, ischemia, fracture, and tumor, comparing Brainfound with a RadImageNet pre-trained baseline.

(R and S) Distributions of per-scan posterior probabilities for ischemia (R) and hemorrhage (S).

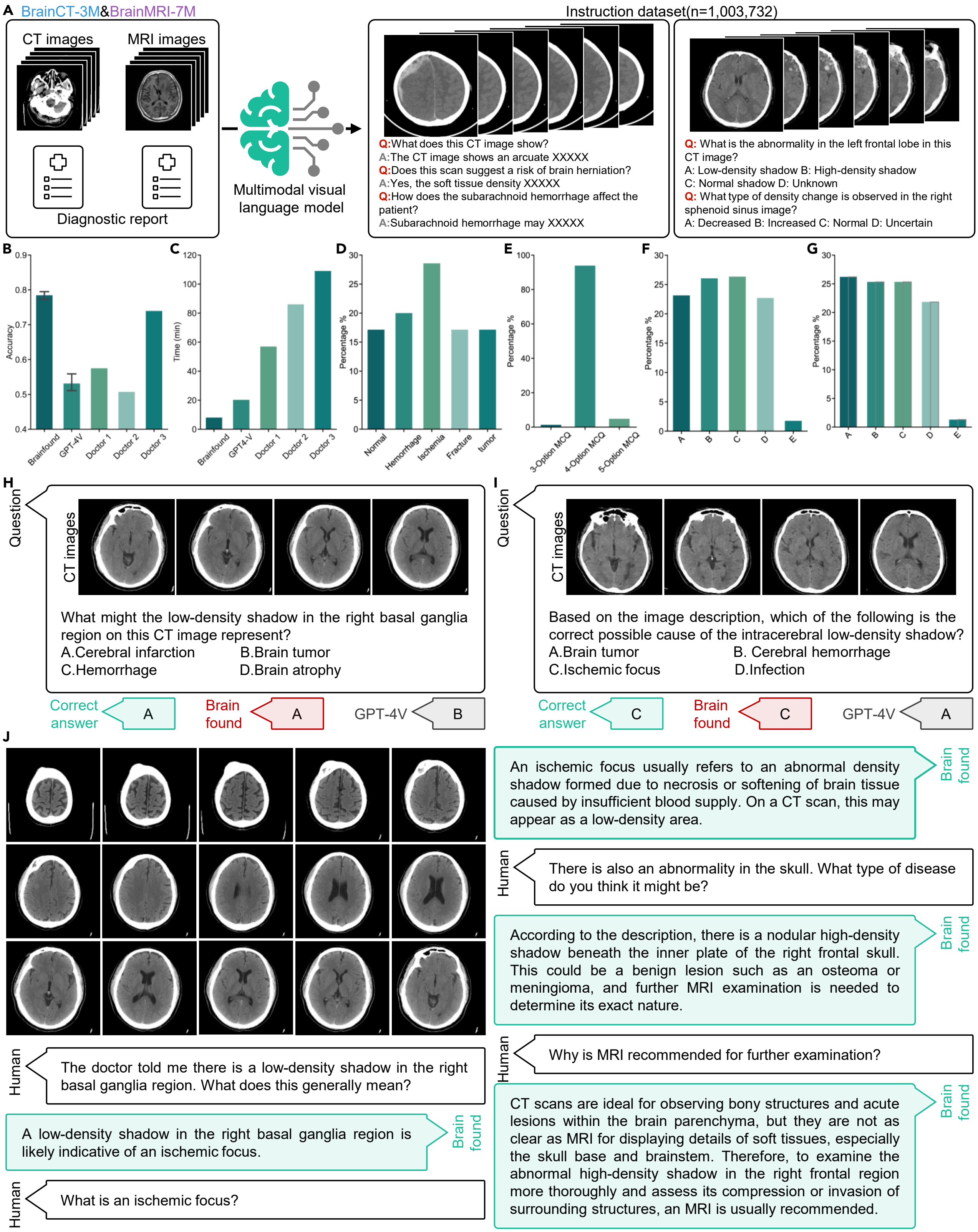

(legend on next page)

with three rounds of dialogue. To increase task diversity, we designed various prompts for GPT to generate data across different knowledge depths and perspectives.

To evaluate the performance of Brainfound on MCQs, we created a test set called BrainMCQ, comprising 70 brain CT scan samples (12 normal, 14 hemorrhage, 20 ischemia, 12 fracture, and 12 tumor; Figure 5D). Each CT scan was paired with three or four MCQs, yielding 229 questions (3 with three options, 215 with four, and 11 with five; Figure 5E). Answer keys were randomly distributed to avoid option bias (Figure 5F). Three radiologists (2–5 years of experience) completed the test alongside Brainfound and GPT-4V. Both AI models were evaluated over three independent runs, and mean accuracy was reported. Brainfound achieved 0.785 accuracy, comparable to the best-performing human radiologist (0.739) and substantially higher than GPT-4V (0.531; Figure 5A). In terms of efficiency, Brainfound and GPT-4V completed the test in <30 min, while radiologists required $\sim$60 min (Figure 5B). Representative cases are shown in Figures 5H, 5I, S36, and S37.

Beyond BrainMCQ, we further explored Brainfound as a potential AI assistant for brain imaging through open-ended clinical dialogues. In these dialogues, Brainfound demonstrated the ability to integrate brain CT images with medical knowledge, assisting in disease interpretation, responding to complex clinical queries, and suggesting additional examinations when diagnostic uncertainty remained (Figure 5J). To qualitatively demonstrate the model's dialogue capability, representative examples are provided in Video S1. In a conversation about cerebral infarction, Brainfound discussed potential etiologies and also highlighted a possible concurrent hemorrhage. In another case involving cerebral hemorrhage, the model described the potential effects on the lateral ventricles and subarachnoid space, helping to facilitate patient-oriented understanding.

DISCUSSION

In this study, we developed Brainfound, a domain-specific multimodal foundation model for brain medical imaging (Figure S38). We assembled two large-scale paired datasets, BrainCT-3M and BrainMRI-7M, encompassing common imaging modalities and disease types, and further generated an instruction dataset, BrainInstru-1M, containing over 1 million imaging-text pairs. Using a diffusion-based framework as the vision module, Brainfound was pre-trained by contrastive learning for image-text alignment. In downstream evaluations, Brainfound consistently outperformed existing baselines: it achieved higher AUCs in intracranial hemorrhage classification across varying data volumes, higher Dice scores in hemorrhage and midline shift segmentation, and improved performance in automatic report generation, as validated by five independent radiologists. Aligning image and text encoders also enabled zero-shot classification, where Brainfound surpassed RadImageNet on both internal and external test datasets. Beyond structured tasks, Brainfound demonstrated multi-modal understanding through MCQs and free-form dialogues, achieving accuracy comparable to that of human radiologists and showing the capacity to provide clinically relevant explanations and suggestions.

We further investigated calibrated uncertainty estimation and abstention behavior to assess the safety of our model in high-stakes clinical settings. In high-stakes clinical deployment, calibrated confidence estimation is essential for trustworthy human-AI collaboration. To better align our system with this requirement, we further validated an uncertainty-aware abstention strategy on ICH classification. Using Monte Carlo dropout and mutual information as the uncertainty ranking criteria, we showed that abstaining from high-uncertainty predictions consistently improves performance on the retained cases, including higher AUC/F1 and lower NLL/ECE/Brier (Figure S39; Table S1).

Beyond predictive reliability, fairness and subgroup robustness are also critical for clinical translation. We therefore conducted demographic subgroup analyses on the ICH segmentation task across sex- and age-based populations (Figures S40 and S41). Segmentation performance (Dice) was consistent across male and female groups and remained stable across age bins, with no statistically significant or practically meaningful disparities. Together, these results strengthen the trustworthiness and equity considerations of the proposed model for real-world neuroimaging workflows.

Clinical AI models for neurological disorders rely on multimodal integration across diverse data sources. In future iterations of Brainfound, we plan to extend beyond CT and MRI to include additional modalities such as electroencephalogram (EEG) and electronic medical records, positioning longitudinal patient records as central inputs to diagnostic prompts. By further incorporating structured medication data, Brainfound could evolve into a system capable of recommending pharmacological interventions. Training with large-scale multi-modal corpora, including clinical guidelines and biomedical literature, may enable the model to develop abilities that connect

Figure 5. Evaluation of Brainfound on multiple-choice question answering and free conversation

(A) A multi-modal brain imaging instruction dataset with 1,003,732 instruction-response pairs is synthesized using GPT-4. For each scan sequence and its paired report from BrainCT-3M and BrainMRI-7M, we generated multiple-choice questions and multi-turn dialogues covering image interpretation, lesion attributes, and diagnostic reasoning.

(B) Response accuracy on BrainMCQ for Brainfound, GPT-4V, and two experienced clinicians. Brainfound and GPT-4V are each evaluated in three runs. Error bars show the 95% confidence interval.

(C) Completion time on BrainMCQ for Brainfound, GPT-4V, and two experienced clinicians. Bars report the mean across three runs for Brainfound and GPT-4V.

(D) Distribution of question categories in BrainMCQ across normal, cerebral hemorrhage, cerebral ischemia, brain tumor, and fracture.

(E) Distribution of question formats in BrainMCQ with three, four, and five options.

(F) Distribution of correct options in BrainMCQ.

(G) Distribution of selected options for Brainfound and GPT-4V.

(H and I) Two representative BrainMCQ examples. CT sequences and questions are provided as input and model predictions are displayed.

(J) An example of free conversation with Brainfound. CT sequences provide the visual context and a human engages in multi-turn dialogue grounded in the image content.

fragmented imaging information in high-dimensional feature space. A key priority may be temporal causal modeling, allowing the system to integrate imaging changes across time and to anticipate disease trajectories, thereby supporting more precise prognostic assessment and treatment planning.

To ensure clinical applicability, Brainfound must be both computationally efficient and deployable across diverse health-care infrastructures. We envision combining model compression techniques, such as distillation, quantization, and pruning, with secure cloud-based platforms to achieve scalable real-time inference. Beyond technical efficiency, these strategies are essential for embedding AI support seamlessly into clinical workflows. Ultimately, Brainfound is intended to function as a trusted AI foundation model for clinicians, augmenting diagnostic decision-making, enabling personalized therapy recommendations, and broadening access to advanced neuroimaging expertise in global health-care settings.

METHODS

Ethical statement

This retrospective study was approved by the Ethics Committee of Chinese PLA General Hospital (approval no. S2025-007-0). The study was conducted in accordance with the ethical standards of the institutional research committee. The requirement for written informed consent was waived by the ethics committee due to the retrospective nature of the study, which involved the analysis of anonymized existing data.

Pre-training strategy of Brainfound

To establish Brainfound as an outstanding multi-modal AI assistant for brain imaging analysis, our model was designed with three main components: an image encoder, an image decoder, and a large language model (LLM). We further propose a three-stage training strategy to enhance its performance. In the first stage, we adopt a diffusion model-based training approach as the pre-training strategy for the image encoder and decoder. This stage enables the model to effectively capture low-level features from medical images, which is essential for tasks such as segmentation, denoising, and modality conversion. In the second stage, we implement contrastive learning based on the pre-trained image encoder and the ChineseBERT model. Since this stage aims to learn stable sentence-level semantic representations for large-scale contrastive optimization, we adopted ChineseBERT, which provides robust bidirectional sentence embeddings, enabling efficient inference and stable training for image-text alignment. Moreover, it is further trained on Chinese brain CT/MRI radiology reports to enhance the modeling of Chinese linguistic characteristics and medical terminology, thereby improving the reliability of radiology-oriented image-text semantic alignment. This stage establishes a shared embedding space that supports downstream tasks such as report retrieval and zero-shot classification. In the third stage, we fine-tune the image encoder from the second stage and the large language model, InternLM, which is a transformer model in the style of LLaMA (large language model Meta AI), using multi-modal dialogue datasets and MCQ datasets. This stage transfers the aligned visual representations into a generative framework, enabling the final model to function as a general-purpose AI assistant for medical question answering and interactive clinical support.

Self-supervised training for feature representation

To accommodate a broader range of low-level downstream tasks, we adopted the training methodology of diffusion models as our first-stage pre-training strategy. Diffusion models are widely recognized for their ability to generate realistic images from Gaussian noise. Recent research has shown that these models can effectively capture stable prior knowledge, leading to improved performance across a variety of downstream tasks. Therefore, we leveraged diffusion models as a self-supervised pre-training approach. Before training, the images were pre-processed by converting them into different window widths and window levels. The training process of diffusion models consists of two key phases: the forward diffusion process and the reverse diffusion process. During the forward diffusion phase, noise is gradually added to the data. The objective of DDPM (denoising diffusion probabilistic models) is to train a model capable of reconstructing the original data from these noisy observations. For our training, we adhered to the standard settings.[37] To better control the model's generated content and expand its range of applications, we adopted a cross-attention-based DDPM model. The window width, window level, and modality information of the image are used as conditional inputs to guide the learning process during generation. To improve robustness, the conditional information may be randomly dropped out during training.

Contrastive learning

Contrastive learning is a self-supervised learning technique that trains models on unlabeled data by learning meaningful representations through the similarities among data samples. This approach is particularly effective in scenarios with limited labeled data. By utilizing contrastive learning, the image encoder extracts features that align with semantically meaningful text in the feature space. This alignment facilitates applications such as image-text retrieval and medical image captioning. In this stage, we use the encoder from the pre-trained model in the first stage to extract image features from an image sequence. These features are concatenated and then passed through an aggregation module and a projection module to generate a feature vector. This feature vector is compared with the features extracted by a text encoder to calculate similarity, and the loss is computed accordingly. For the text encoder, we employed a BERT structure fine-tuned on Chinese-language corpora. Using a rule-based report analysis method, we extracted CT image categories, including normal, hemorrhage, cerebral infarction, fracture, and tumor. Since MRI scans encompass multiple distinct modality sequences, the report content was characterized by its comprehensive and summarized nature, and we leveraged ChatGPT to extract key disease-related terms from the reports to ensure an accurate and comprehensive representation of disease information. When constructing the text for contrastive learning, we concatenated the extracted disease categories with the original reports.

Multi-modal fine-tuning phase

To enable the multi-modal assistant to fully understand both images and text, we fine-tuned the model using the image encoder from the contrastive learning stage and a large language model based on the open-source InternLM architecture. When

constructing the multi-modal training dataset, we utilized ChatGPT to clean and organize the report data. By designing prompts, we transformed the reports into conversational text of various styles and created MCQs. Detailed prompts are provided in the supplemental information. Using this approach, we generated a total of ~10,000 rounds of dialogue text and ~500 sets of MCQ text. Given the significant number of parameters in large models, fully fine-tuning all parameters for downstream tasks requires substantial computational resources and is prone to overfitting. Moreover, full fine-tuning can lead to severe forgetting issues, causing the model to lose many of its original capabilities. To address these challenges, we adopted the PEFT (parameter-efficient fine-tuning) method based on LoRA[57] (low-rank adaptation) to fine-tune both the InternLM language model and the image encoder.

Network architecture

Our Brainfound framework consists of an image encoder, an image decoder, and a foundational large language model. Brainfound leverages diffusion models for pre-training to obtain robust and meaningful feature representations. During the self-supervised diffusion phase, the image encoder and image decoder are connected in a UNet-like architecture. To better capture feature representations across multiple levels, we chose a pixel-space diffusion model instead of the latent diffusion model (LDM).[58] Specifically, the image encoder in Brainfound consists of five downsampling modules and one deep feature extraction module, while the image decoder comprises five upsampling modules. Each of these modules incorporates residual structures to ensure effective gradient optimization during training.[59] Moreover, certain downsampling and upsampling blocks are enhanced with a cross-attention transformer module. This mechanism enables the encoded textual information to directly influence the image generation process. Such textual information includes, but is not limited to, parameters like the image's window width and window level as well as disease category information extracted from reports.

In the contrastive learning phase, we additionally designed an aggregation module to fuse the features of multiple images within an image sequence. The aggregation module adopts a transformer architecture consisting of two layers of transformer encoders with layer normalization. Positional encoding parameters are also included to enhance the model's ability to process sequential information.

During the fine-tuning phase of the multi-modal assistant, we selected InternLM as the foundation for the large language model. InternLM is a highly optimized large-scale language model based on the transformer architecture, designed for performance and efficiency. Its core structure incorporates multi-head self-attention (MHSA) to capture long-range dependencies efficiently, enabling parallel processing of contextual relationships and maintaining semantic and syntactic consistency during generation. To enhance contextual understanding, InternLM employs rotary position embedding (RoPE), which provides strong generalization capabilities for modeling long sequences. In this work, we utilized the 7B-parameter version of the model to balance between performance requirements and computational resources. We adopted an instruction learning approach to fine-tune the image encoder and the InternLM model using multi-modal dialogue data and multi-modal MCQ datasets. Specifically, for a given dialogue, we concatenated the image-encoded features with the text-extracted tokens and input them into the InternLM model to generate outputs. A detailed process flowchart can be found in Figure S1.

We report detailed inference efficiency metrics measured on an NVIDIA GeForce RTX 3090 GPU across representative medical imaging workloads. For diffusion-based image generation with 1,000 denoising steps, inference requires an average latency of approximately 65 s per image, corresponding to a computational cost of 101.7 GFLOPs. Peak GPU memory consumption is 617 MB at a batch size of 1, and the resulting model size is 78.1 MB. For image classification, inference on a single image incurs 28.0 GFLOPs with an average latency of approximately 203 ms. Peak GPU memory usage reaches 1.27 GB at a batch size of 16, while the model remains compact at 19.7 MB. For image segmentation, inference requires 101.7 GFLOPs and approximately 548 ms latency per image. Owing to the additional segmentation head, peak memory consumption increases to 3.14 GB at a batch size of 16, with a corresponding model size of 78.6 MB.

Training datasets for each task

Training data for intracranial hemorrhage classification

We used the publicly available RSNA intracranial hemorrhage classification dataset comprising 222,218 axial brain CT images with slice-level labels. No additional private data were used. In the calibrated confidence estimation experiment, we randomly split the validation set into two disjoint subsets of equal size (50%/50%). On the first subset, we selected per-class abstention thresholds (τ) by targeting pre-defined coverage levels on the basis of MI-ranked uncertainty scores. The selected thresholds were then fixed and applied to the remaining validation subset, which was used exclusively for performance evaluation.

Training data for brain hemorrhage segmentation

We used 2,060 brain CT scans from the Chinese PLA General Hospital. The dataset was split into 220 training cases (1,397 images), 760 validation cases (5,440 images), and 1,080 test cases (7,917 images). For data-efficiency experiments, the training set was subsampled at 12.5%, 25%, 50%, and 100% of its images. For the subgroup analyses across age and sex, the validation cohort was stratified by sex as male (n = 682), female (n = 397), and unknown/other (n = 1). Alternatively, cases were stratified into five fixed age groups: <20, 20–39, 40–59, 60–79, and $\geq$80 years.

Training data for brain midline localization and segmentation

We used brain CT data from 301 patients. The dataset was split into a training set of 12 cases (439 images), a validation set of 50 cases (1,629 images), and a test set of 239 cases (7,743 images). For data efficiency experiments, the training set was subsampled at 12.5% (1 case, 72 images), 25% (3 cases, 138 images), 50% (6 cases, 236 images), and 100% (12 cases, 439 images).

Training data for zero-shot MRI denoising

We selected 10 T1-weighted 3 T MRI scans from BraTS 2023, totaling 1,380 slices. To create test inputs at different noise levels, we added synthetic Rician noise to the clean images and generated six noisy versions per scan with average image SNRs of 9.6808, 11.7425, 14.7979, 15.6190, 16.5326, and 17.5389 dB.

Training data for paired MRI denoising across field strengths

We assembled paired high- and low-SNR MRI data spanning 0.3 T to 5 T. The 0.3 T low field data came from M4Raw and included 25 T1WI scans (450 images), 25 T2WI scans (450 images), and 25 FLAIR scans (450 images). The 5 T ultra-high-field portion comprised an internal clinical set from Beijing Friendship Hospital with 1 T2WI scan (10 images) and 1 T1WI scan (19 images), and an external 5 T test set provided by Shanghai United Imaging with 25 images.

Training data for MRI modality transformation

We used 182 brain 3 T MRI cases from the Chinese PLA General Hospital spanning five sequences (T1WI, T2WI, FLAIR, low-b-value DWI, and standard-b-value DWI). For modality transformation, T1WI served as the source and the other four sequences as targets. The data were split into training 94 scans (2,205 images) and test 88 scans (1,936 images).

Training data for automatic brain CT report generation

Evaluation employed 990 brain CT scans with paired clinician-written reports from the Chinese PLA General Hospital, which were reserved exclusively as a test set and were not included in any pre-training. For clinician scoring, a separate 33-case subset was randomly sampled from the same cohort for human evaluation; this subset was likewise used only for evaluation, not for training.

Training data for zero-shot brain CT classification

The internal set comprised 588 scans from the Chinese PLA General Hospital with five diagnostic categories: normal (190), cerebral hemorrhage (71), cerebral ischemia (122), skull fracture (173), and brain tumor (32). The external set comprised 363 scans from Brain Hospital of Hunan Province: normal (92), cerebral hemorrhage (62), cerebral ischemia (160), skull fracture (24), and brain tumor (25). None of these data were included in pre-training.

Training data for BrainMCQ

For evaluation, we created BrainMCQ, which includes 70 brain CT scans with diagnostic categories: normal (12), cerebral hemorrhage (14), cerebral ischemia (20), skull fracture (12), and brain tumor (12). Each scan contributes 3 or 4 MCQs, yielding 229 questions in total (3 with three options, 215 with four options, and 11 with five options). The position of the correct option was randomized to avoid option bias.

Fine-tuning Brainfound to downstream tasks

To fully unlock the potential of Brainfound across diverse tasks, we incorporated multiple state-of-the-art deep learning techniques and designed experiments tailored to various downstream applications.

RSNA intracranial hemorrhage classification task

Intracranial hemorrhage classification is essential for identifying the underlying cause of bleeding, guiding treatment decisions, and optimizing management strategies. It provides a foundation for prognosis evaluation, personalized treatment planning, and advancing medical research. In this task, we utilized an image encoder to extract image features and perform classification through an additional linear layer. Specifically, the image at $t = 0$ is input into the image encoder to extract features, which are then passed through a dropout layer and an activation function before being fed into the linear layer for prediction. As this is a multi-label classification problem, binary cross-entropy (BCE) is employed to calculate the loss. We evaluated three experimental setups on this dataset: full-parameter fine-tuning, in which both the image encoder and the linear layer parameters are updated during training; linear-layer fine-tuning, in which the image encoder is frozen and only the linear layer weights are fine-tuned; and ensemble integration, in which our image encoder was incorporated into the winning ensemble strategy of the RSNA competition for further evaluation.

ICH and midline structure segmentation task

Brainfound provides rich prior knowledge of brain medical images. To fully exploit this prior knowledge for segmentation, which is a dense prediction task, we adopted the approach[60] that utilized MLP to classify each pixel's label. In summary, we used the image encoder and image decoder of Brainfound to extract image features and trained an MLP classifier to classify the features extracted from each spatial location. For each image, we obtained four multi-scale feature maps from Brainfound, which were then upsampled to match the input resolution and concatenated. The feature vector corresponding to each spatial location was then fed into the MLP classifier to predict the class of that pixel, and the loss was computed with the segmentation labels to update the network. During training, we used single-center data and split it into training, validation, and testing sets. The best model was selected based on the validation set, and results were reported on the test set. The AdamW optimizer was used with a weight decay of $1e{-}3$ and an initial learning rate of $1e{-}3$. The training lasted for 20 epochs.

MRI modality translation task

For the task of MRI modality conversion, we conducted four experiments, converting T1WI into T2WI, FLAIR, and DWI, with the last further divided into two classes: $b < 500$ and $b > 500$. For this task, we employed a straightforward conditional diffusion model to perform the modality conversion. Specifically, the input to the diffusion model consisted of both the noise channel and an additional T1WI as the condition. We acknowledge that more advanced diffusion-based mechanisms might achieve better results in this task. For our experiments, we curated a dataset of 200 cases containing these modalities. Among them, 100 cases were used for training, and the remaining 100 were used for validation. The model was trained for 200 epochs, and the final model was evaluated on the validation set.

Low-quality medical image enhancement task

The visual module of Brainfound employs the DDPM strategy for pre-training, which provides strong representation learning capabilities for pixel-level semantic information. This capability is leveraged to develop a zero-shot denoising framework. For the detailed algorithmic process, see Figure S13.

Zero-shot classification task

To validate the effectiveness of contrastive learning, we collected two classification datasets. The first dataset, consisting of 588 cases, was sourced from an internal center and was entirely separate from the training data used for contrastive learning. The second dataset, obtained from an external center, contains 363 cases. Both datasets include five categories: normal, hemorrhage, ischemia, fracture, and tumor. For zero-shot classification, we constructed textual features for the five

categories using short descriptive phrases. The cosine similarity between the textual features and the image features was then computed. After the similarity scores were normalized, softmax probabilities were calculated to predict the final category.

Medical image report generation task

During the training of contrastive learning, we additionally designed a text decoder module. This module is based on a pretrained Chinese BERT architecture with six hidden layers and a vocabulary size of 21,128. The module takes image features as input and predicts the probability distribution of the corresponding text. The predicted results are further refined using a beam search algorithm to generate the final version of the medical image report. In this task, we compared our approach with several baseline models (RadFM, MiniGPT-Med, and GPT-4V), all of which can generate reports based on images. For RadFM and MiniGPT-Med, we utilized the prompts provided in the authors' examples to generate report outputs from medical images. For GPT-4V, we designed custom prompts as input. Detailed prompts can be found in the supplemental information. For evaluation, we adopted standard quantitative metrics such as BLEU, ROUGE-L, and METEOR. Additionally, we invited five physicians to rank the reports generated by the four methods. The evaluation criteria were designed based on physicians' suggestions and included 10 subcategories. The ranking method is similar to GPA (grade point average) calculation, where the best items are assigned 4 points, the worst are given 1 point, and certain unacceptable cases are assigned 0 points.

AI assistant assessment task

To validate the functionality of the multi-modal assistant, we designed two experiments: MCQ evaluation and free-form question answering. For the MCQs, we compared our model, Brainfound, with GPT-4o. Two physicians were invited to complete the questions as well. Both Brainfound and GPT-4o were generally able to provide consistent and well-formatted answers. However, GPT-4o occasionally failed to answer certain questions. For these cases, we repeatedly queried the API until stable responses were obtained. We compared the performances of Brainfound, GPT-4o, and the two physicians in terms of answer accuracy and response time. The detailed results are presented in Figure 5.

Evaluation metrics for pixel-level tasks

Several metrics are commonly used to evaluate the performance of image enhancement tasks. Among them, the PSNR is widely recognized as a standard for assessing image quality. A higher PSNR value indicates better image fidelity. If the ground truth image is y, and the raw image is x, then the definition of PSNR is as follows:

$$\mathrm{PSNR} = 20 \times \log 10(\mathrm{MAX} / \mathrm{MSE}) \quad \mathrm{MSE} = \|x\text{-}y\|^2 \big/ (m \times n).$$

Here MAX is the maximum pixel value. For normalized images, MAX = 1. m and n are the two dimensions of the image. We also compute the SNR to evaluate the performance of various methods and, the formula is as follows:

$$\mathrm{SNR} = 10 \times \log 10\left(y^2 / \mathrm{MSE}\right).$$

RMSE directly quantifies the variance between two images. An RMSE value approaching 0 indicates better preservation of visual information between the reconstructed image and the ground truth. RMSE is defined as follows:

$$\mathrm{RMSE} = \mathrm{MSE}^{1/2}.$$

SSIM is a widely used metric for quantifying the similarity between two images. SSIM evaluates similarity by independently comparing three key components: luminance, contrast, and structural information. These components are then weighted and combined into a single score to represent the overall similarity. The calculation of SSIM is performed using a sliding window applied across the image. In this process, a window with dimensions $a \times a$ is selected from the image for each calculation, and the SSIM is computed for that specific window. The overall SSIM for the image is then obtained by averaging the SSIM values from all such windows after the entire image has been scanned. A higher SSIM score indicates superior image quality.

Evaluation metrics for report generation

BLEU is a widely used metric for evaluating the quality of machine-generated text, particularly in tasks such as machine translation and text summarization. The BLEU score is calculated based on n-gram precision, which measures the overlap between n-grams in the generated text and those in the reference text. BLEU-1 to BLEU-4 represent the scores computed using unigrams, bigrams, trigrams, and 4-grams, respectively, capturing different levels of linguistic context. The formula is as follows:

$$\mathrm{BLEU} = \mathrm{BP} \times \exp(\mathrm{W} \times \log \mathrm{P}).$$

BP is the brevity penalty, which addresses the issue of overly short translations. P is the precision for grams. W is the weight assigned to each gram precision.

ROUGE-L (recall-oriented understudy for gisting evaluation-longest common subsequence) is a widely used metric for evaluating the quality of machine-generated text, particularly in summarization tasks. Unlike n-gram-based metrics, ROUGE-L measures the overlap between the candidate text and the reference text based on their longest common subsequence (LCS). This approach takes into account both the order and the presence of words, making it well suited to capturing fluency and relevance in text generation.

METEOR (metric for evaluation of translation with explicit ordering) is a popular evaluation metric for machine-generated text, particularly in machine translation and text generation tasks. Unlike n-gram-based metrics such as BLEU, METEOR focuses on aligning words in the candidate and reference texts using advanced matching techniques, making it more robust and sensitive to variations in word order and synonymy.

Visualization of saliency maps

The Grad-CAM[61] technique is harnessed to craft the saliency map for the input image model. Initially, the activation feature maps of the convolutional layers are derived via forward propagation, and subsequently, the gradients of these feature maps concerning the target class are computed through backpropagation. Following this, global average pooling is applied to these gradients to acquire the channel weights. These weights are then utilized to modulate the activation feature maps of the convolutional layers, culminating in a 2D heatmap of weighted

summation, elucidating the significance of distinct regions within the input image for the target category. Following this, the heatmap undergoes upscaling to match the input image's dimensions using bilinear interpolation. Last, the heatmap is rendered visually through color mapping to exhibit the areas of interest identified by the model. The contour map delineates lines of uniform value within a saliency map.

RESOURCE AVAILABILITY

Lead contact

Requests for further information and resources should be directed to and will be fulfilled by the lead contact, Yuchen Guo (yuchen.w.guo@gmail.com).

Materials availability

This study did not generate new unique reagents.

Data and code availability

The source code and pre-trained model weights of the Brainfound foundation model have been deposited in Zenodo[62] and are publicly available under a persistent DOI: https://doi.org/10.5281/zenodo.18976379. The training datasets used in this study contain sensitive clinical information and cannot be publicly shared due to ethical and privacy restrictions. Access to the data could be considered upon reasonable request and subject to approval by the corresponding institutions and ethics committee. The BraTS 2023 dataset is available from the Brain Tumor Segmentation (BraTs) challenge (https://www.synapse.org/#!Synapse:syn51156910), and the RSNA Intracranial Hemorrhage dataset is available from the RSNA Intracranial Hemorrhage Detection Challenge on Kaggle (https://www.kaggle.com/competitions/rsna-intracranial-hemorrhage-detection). Access to these dataset may require registration and agreement to the respective data usage terms.

ACKNOWLEDGMENTS

This work was supported by the National Natural Science Foundation of China (NSFC) (nos. 82441013, 82441014, 62088102, 82327803, T2541076, T2541074, and 82572167).

AUTHOR CONTRIBUTIONS

Q.D., X.L., F.X., and Yuchen Guo conceived the Brainfound project and revised the manuscript. G.Z. and Z.G. implemented the Brainfound framework, trained the multi-modal model, completed the fine-tuning of downstream tasks, organized the experimental results, and composed the manuscript. C.D. collected data and established the BrainCT-3M and BrainMRI-7M datasets. J.L. completed the saliency visualization of Brainfound attention. T.W., Yanchen Guo, and Y.C. established the BrainInstru-1M instruction dataset. L.W. collected 5 T brain MRI data. Y. Lizhu, Y. Liu, Q.C., K.F., and L.W. completed the human-machine evaluation of automatic report generation. Y. Lizhu, Y. Liu, Q.C., K.F., and L.W. completed the BrainMCQ evaluation. T.W., Y.C., and Yanchen Guo contributed to the preparation of the supplemental figures.

DECLARATION OF INTERESTS

Q.D. is on the advisory board of *Patterns*.

DECLARATION OF GENERATIVE AI AND AI-ASSISTED TECHNOLOGIES IN THE WRITING PROCESS

No generative AI or AI-assisted technologies were used in the preparation of this work.

SUPPLEMENTAL INFORMATION

Supplemental information can be found online at https://doi.org/10.1016/j.patter.2026.101538.

Received: September 14, 2025
Revised: January 19, 2026
Accepted: March 17, 2026
Published: April 14, 2026

REFERENCES

1. Wang, J., Wang, K., Yu, Y., Lu, Y., Xiao, W., Sun, Z., Liu, F., Zou, Z., Gao, Y., Yang, L., et al. (2025). Self-improving generative foundation model for synthetic medical image generation and clinical applications. Nat. Med. *31*, 609–617. https://doi.org/10.1038/s41591-024-03359-y.

2. Pai, S., Bontempi, D., Hadzic, I., Prudente, V., Sokač, M., Chaunzwa, T.L., Bernatz, S., Hosny, A., Mak, R.H., Birkbak, N.J., and Aerts, H.J.W.L. (2024). Foundation model for cancer imaging biomarkers. Nat. Mach. Intell. *6*, 354–367. https://doi.org/10.1038/s42256-024-00807-9.

3. Hamed, A.A., Hua, K., Trinh, Q.M., Simons, B.D., Marioni, J.C., Stein, L.D., and Dirks, P.B. (2025). Gliomagenesis mimics an injury response orchestrated by neural crest-like cells. Nature *638*, 499–509. https://doi.org/10.1038/s41586-024-08356-2.

4. Sun, Y., Wang, L., Li, G., Lin, W., and Wang, L. (2025). A foundation model for enhancing magnetic resonance images and downstream segmentation, registration and diagnostic tasks. Nat. Biomed. Eng. *9*, 521–538. https://doi.org/10.1038/s41551-024-01283-7.

5. Busch, E.L., Huang, J., Benz, A., Wallenstein, T., Lajoie, G., Wolf, G., Krishnaswamy, S., and Turk-Browne, N.B. (2023). Multi-view manifold learning of human brain-state trajectories. Nat. Comput. Sci. *3*, 240–253. https://doi.org/10.1038/s43588-023-00419-0.

6. Lu, M.Y., Chen, B., Williamson, D.F.K., Chen, R.J., Zhao, M., Chow, A.K., Ikemura, K., Kim, A., Pouli, D., Patel, A., et al. (2024). A multimodal generative AI copilot for human pathology. Nature *634*, 466–473. https://doi.org/10.1038/s41586-024-07618-3.

7. Huang, Z., Bianchi, F., Yuksekgonul, M., Montine, T.J., and Zou, J. (2023). A visual–language foundation model for pathology image analysis using medical Twitter. Nat. Med. *29*, 2307–2316. https://doi.org/10.1038/s41591-023-02504-3.

8. Li, C., Wong, C., Zhang, S., Usuyama, N., Liu, H., Yang, J., Naumann, T., Poon, H., and Gao, J. (2023). LLaVA-Med: Training a Large Language-and-Vision Assistant for Biomedicine in One Day. Adv. Neural Inform. Process. Syst. https://doi.org/10.5555/3666122.3667362.

9. Baid, U., Ghodasara, S., Mohan, S., Bilello, M., Calabrese, E., Colak, E., Farahani, K., Kalpathy-Cramer, J., Kitamura, F.C., Pati, S., et al. (2021). The RSNA-ASNR-MICCAI BraTS 2021 Benchmark on Brain Tumor Segmentation and Radiogenomic Classification. Preprint at arXiv. https://doi.org/10.48550/arXiv.2107.02314.

10. Iqbal, A., Khan, R., and Karayannis, T. (2019). Developing a brain atlas through deep learning. Nat. Mach. Intell. *1*, 277–287. https://doi.org/10.1038/s42256-019-0058-8.

11. Handwerker, J., Pérez-Rodas, M., Beyerlein, M., Vincent, F., Beck, A., Freytag, N., Yu, X., Pohmann, R., Anders, J., and Scheffler, K. (2019). A CMOS NMR needle for probing brain physiology with high spatial and temporal resolution. Nat. Methods *17*, 64–67. https://doi.org/10.1038/s41592-019-0640-3.

12. Cheng, J., Liu, Z., Guan, H., Wu, Z., Zhu, H., Jiang, J., Wen, W., Tao, D., and Liu, T. (2021). Brain Age Estimation From MRI Using Cascade Networks With Ranking Loss. IEEE Trans. Med. Imaging *40*, 3400–3412. https://doi.org/10.1109/TMI.2021.3085948.

13. Lyu, Q., and Wang, G. (2022). Conversion Between CT and MRI Images Using Diffusion and Score-Matching Models. Preprint at arXiv. https://doi.org/10.48550/arXiv.2209.12104.

14. Bercea, C.I., Wiestler, B., Rueckert, D., and Albarqouni, S. (2022). Federated disentangled representation learning for unsupervised brain anomaly detection. Nat. Mach. Intell. *4*, 685–695. https://doi.org/10.1038/s42256-022-00515-2.

CellPress
OPEN ACCESS

15. He, S., Grant, P.E., and Ou, Y. (2022). Global-Local Transformer for Brain Age Estimation. IEEE Trans. Med. Imaging *41*, 213–224. https://doi.org/10.1109/TMI.2021.3108910.

16. Hollon, T.C., Pandian, B., Adapa, A.R., Urias, E., Save, A.V., Khalsa, S.S.S., Eichberg, D.G., D'Amico, R.S., Farooq, Z.U., Lewis, S., et al. (2020). Near real-time intraoperative brain tumor diagnosis using stimulated Raman histology and deep neural networks. Nat. Med. *26*, 52–58. https://doi.org/10.1038/s41591-019-0715-9.

17. Menze, B.H., Jakab, A., Bauer, S., Kalpathy-Cramer, J., Farahani, K., Kirby, J., Burren, Y., Porz, N., Slotboom, J., Wiest, R., et al. (2015). The Multimodal Brain Tumor Image Segmentation Benchmark (BRATS). IEEE Trans. Med. Imaging *34*, 1993–2024. https://doi.org/10.1109/TMI.2014.2377694.

18. Chen, E., Prakash, S., Janapa Reddi, V., Kim, D., and Rajpurkar, P. (2025). A framework for integrating artificial intelligence for clinical care with continuous therapeutic monitoring. Nat. Biomed. Eng. *9*, 445–454. https://doi.org/10.1038/s41551-023-01115-0.

19. Hoopes, A., Mora, J.S., Dalca, A.V., Fischl, B., and Hoffmann, M. (2022). SynthStrip: skull-stripping for any brain image. Neuroimage *260*, 119474. https://doi.org/10.1016/j.neuroimage.2022.119474.

20. Ji, M., Lewis, S., Camelo-Piragua, S., Ramkissoon, S.H., Snuderl, M., Venneti, S., Fisher-Hubbard, A., Garrard, M., Fu, D., Wang, A.C., et al. (2015). Detection of human brain tumor infiltration with quantitative stimulated Raman scattering microscopy. Sci. Transl. Med. *7*, 309ra163. https://doi.org/10.1126/scitranslmed.aab0195.

21. Guo, Y., He, Y., Lyu, J., Zhou, Z., Yang, D., Ma, L., Tan, H.T., Chen, C., Zhang, W., Hu, J., et al. (2022). Deep learning with weak annotation from diagnosis reports for detection of multiple head disorders: a prospective, multicentre study. Lancet Digit. Health *4*, e584–e593. https://doi.org/10.1016/S2589-7500(22)00090-5.

22. Moguilner, S., Baez, S., Hernandez, H., Migeot, J., Legaz, A., Gonzalez-Gomez, R., Farina, F.R., Prado, P., Cuadros, J., Tagliazucchi, E., et al. (2024). Brain clocks capture diversity and disparities in aging and dementia across geographically diverse populations. Nat. Med. *30*, 3646–3657. https://doi.org/10.1038/s41591-024-03209-x.

23. He, Z., Zhu, Y.-N., Chen, Y., Chen, Y., He, Y., Sun, Y., Wang, T., Zhang, C., Sun, B., Yan, F., et al. (2023). A deep unrolled neural network for real-time MRI-guided brain intervention. Nat. Commun. *14*, 8257. https://doi.org/10.1038/s41467-023-43966-w.

24. Khalighi, S., Reddy, K., Midya, A., Pandav, K.B., Madabhushi, A., and Abedalthagafi, M. (2024). Artificial intelligence in neuro-oncology: advances and challenges in brain tumor diagnosis, prognosis, and precision treatment. npj Precis. Onc. *8*, 80. https://doi.org/10.1038/s41698-024-00575-0.

25. Radford, A., Narasimhan, K., Salimans, T., and Sutskever, I. (2023). Improving Language Understanding by Generative Pre-Training. OpenAI Technical Report.

26. Ramesh, A., Dhariwal, P., Nichol, A., Chu, C., and Chen, M. (2022). Hierarchical Text-Conditional Image Generation with CLIP Latents. Preprint at arXiv. https://doi.org/10.48550/arXiv.2204.06125.

27. Chen, T., Kornblith, S., Norouzi, M., and Hinton, G. (2020). A Simple Framework for Contrastive Learning of Visual Representations. International Conference on Machine Learning. https://doi.org/10.5555/3524938.3525087.

28. Zhang, H., Li, F., Liu, S., Zhang, L., Su, H., Zhu, J., Ni, L.M., and Shum, H.-Y. (2022). DINO: DETR with Improved DeNoising Anchor Boxes for End-to-End Object Detection. International Conference on Learning Representations. https://doi.org/10.48550/arXiv.2203.03605.

29. Chen, R.J., Ding, T., Lu, M.Y., Williamson, D.F.K., Jaume, G., Song, A.H., Chen, B., Zhang, A., Shao, D., Shaban, M., et al. (2024). Towards a general-purpose foundation model for computational pathology. Nat. Med. *30*, 850–862. https://doi.org/10.1038/s41591-024-02857-3.

30. Qiu, J., Wu, J., Wei, H., Shi, P., Zhang, M., Sun, Y., Li, L., Liu, H., Liu, H., Hou, S., et al. (2024). Development and Validation of a Multimodal Multitask Vision Foundation Model for Generalist Ophthalmic Artificial Intelligence. NEJM AI *1*, eAIoa2300221. https://doi.org/10.1056/AIoa2300221.

31. Vermeulen, C., Pagès-Gallego, M., Kester, L., Kranendonk, M.E.G., Wesseling, P., Verburg, N., De Witt Hamer, P., Kooi, E.J., Dankmeijer, L., Van Der Lugt, J., et al. (2023). Ultra-fast deep-learned CNS tumour classification during surgery. Nature *622*, 842–849. https://doi.org/10.1038/s41586-023-06615-2.

32. Xiong, Z., Wang, X., and Wong, T. (2025). How Generalizable Are Foundation Models When Applied to Different Demographic Groups and Settings? NEJM *AI 2*, AIcs2400497. https://doi.org/10.1056/AIcs2400497.

33. Grattafiori, A., Dubey, A., Jauhri, A., Pandey, A., Kadian, A., Al-Dahle, A., Letman, A., Mathur, A., Schelten, A., Vaughan, A., et al. (2024). The Llama 3 Herd of Models. Preprint at arXiv. https://doi.org/10.48550/arXiv.2407.21783.

34. Flanders, A.E., Prevedello, L.M., Shih, G., Halabi, S.S., Kalpathy-Cramer, J., Ball, R., Mongan, J.T., Stein, A., Kitamura, F.C., Lungren, M.P., et al. (2020). Construction of a Machine Learning Dataset through Collaboration: The RSNA 2019 Brain CT Hemorrhage Challenge. Radiol. Artif. Intell. *2*, e190211. https://doi.org/10.1148/ryai.2020190211.

35. Vaswani, A., Shazeer, N., Parmar, N., Uszkoreit, J., Jones, L., Gomez, A.N., Kaiser, L., and Polosukhin, I. (2017). Attention Is All You Need. Adv. Neural Inform. Process. Syst. https://doi.org/10.48550/arXiv.1706.03762.

36. Bao, H., Dong, L., Piao, S., and Wei, F. (2022). BEiT: BERT Pre-Training of Image Transformers. International Conference on Learning Representations. https://doi.org/10.48550/arXiv:2106.08254.

37. Ho, J., Jain, A., and Abbeel, P. (2020). Denoising Diffusion Probabilistic Models. Adv. Neural Inform. Process. Syst. *33*, 6840–6851. https://doi.org/10.48550/arXiv.2006.11239.

38. Chen, S., Sun, P., Song, Y., and Luo, P. (2023). DiffusionDet: Diffusion Model for Object Detectio. IEEE/CVF International Conference on Computer Vision (ICCV). IEEE;, 19733–19786. https://doi.org/10.1109/ICCV51070.2023.01816.

39. Bai, Y., Wang, Z., Xiao, J., Wei, C., Wang, H., Yuille, A.L., Zhou, Y., and Xie, C. (2023). Masked Autoencoders Enable Efficient Knowledge Distillers. IEEE/CVF Conference on Computer Vision and Pattern Recognition (CVPR). IEEE;, 24256–24265. https://doi.org/10.1109/CVPR52729.2023.02323.

40. Mei, X., Liu, Z., Robson, P.M., Marinelli, B., Huang, M., Doshi, A., Jacobi, A., Cao, C., Link, K.E., Yang, T., et al. (2022). RadImageNet: An Open Radiologic Deep Learning Research Dataset for Effective Transfer Learning. Radiol. Artif. Intell. *4*, e210315. https://doi.org/10.1148/ryai.210315.

41. Huang, G., Liu, Z., Maaten, L. van der, and Weinberger, K.Q. (2017). Densely Connected Convolutional Networks. Proc. IEEE Conf. Comput. Vis. Pattern Recognit. (CVPR), 4700–4708. https://doi.org/10.1109/CVPR.2017.243.

42. Ma, J., He, Y., Li, F., Han, L., You, C., and Wang, B. (2024). Segment anything in medical images. Nat. Commun. *15*, 654. https://doi.org/10.1038/s41467-024-44824-z.

43. Wang, W., Dai, J., Chen, Z., Huang, Z., Li, Z., Zhu, X., Hu, X., Lu, T., Lu, L., Li, H., et al. (2023). InternImage: Exploring Large-Scale Vision Foundation Models with Deformable Convolutions. IEEE/CVF Conference on Computer Vision and Pattern Recognition (CVPR) (IEEE), pp. 14408–14419. https://doi.org/10.1109/CVPR52729.2023.01385.

44. Zhao, Y., Ding, Y., Lau, V., Man, C., Su, S., Xiao, L., Leong, A.T.L., and Wu, E.X. (2024). Whole-body magnetic resonance imaging at 0.05 Tesla. Science *384*, eadm7168. https://doi.org/10.1126/science.adm7168.

45. Liu, Y., Leong, A.T.L., Zhao, Y., Xiao, L., Mak, H.K.F., Tsang, A.C.O., Lau, G.K.K., Leung, G.K.K., and Wu, E.X. (2021). A low-cost and shielding-free ultra-low-field brain MRI scanner. Nat. Commun. *12*, 7238. https://doi.org/10.1038/s41467-021-27317-1.

46. Kimberly, W.T., Sorby-Adams, A.J., Webb, A.G., Wu, E.X., Beekman, R., Bowry, R., Schiff, S.J., de Havenon, A., Shen, F.X., Sze, G., et al. (2023).

Brain imaging with portable low-field MRI. Nat. Rev. Bioeng. *1*, 617–630. https://doi.org/10.1038/s44222-023-00086-w.

47. Wei, Z., Chen, Q., Han, S., Zhang, S., Zhang, N., Zhang, L., Wang, H., He, Q., Cao, P., Zhang, X., et al. (2023). 5T magnetic resonance imaging: radio frequency hardware and initial brain imaging. Quant. Imaging Med. Surg. *13*, 3222–3240. https://doi.org/10.21037/qims-22-945.
48. Zhang, K., Li, Y., Liang, J., Cao, J., Zhang, Y., Tang, H., Fan, D.-P., Timofte, R., and Gool, L.V. (2023). Practical Blind Image Denoising via Swin-Conv-UNet and Data Synthesis. Mach. Intell. Res. *20*, 822–836. https://doi.org/10.1007/s11633-023-1466-0.
49. Huang, T., Li, S., Jia, X., Lu, H., and Liu, J. (2021). Neighbor2Neighbor: Self-Supervised Denoising from Single Noisy Images. IEEE/CVF Conference on Computer Vision and Pattern Recognition (CVPR) (IEEE), pp. 14776–14785. https://doi.org/10.1109/CVPR46437.2021.01454.
50. Batson, J., and Royer, L. (2019). Noise2Self: Blind Denoising by Self-Supervision. International Conference onf Machine Learning, 524–533. https://doi.org/10.48550/arXiv.1901.11365.
51. Goel, H., Narasimhan, S.S., Akcin, O., and Chinchali, S. (2024). SynDiff-AD: Improving Semantic Segmentation and End-to-End Autonomous Driving with Synthetic Data from Latent Diffusion Models. Preprint at arXiv. https://doi.org/10.48550/arXiv.2411.16776.
52. Dalmaz, O., Yurt, M., and Çukur, T. (2022). ResViT: Residual vision transformers for multi-modal medical image synthesis. IEEE Trans. Med. Imaging *41*, 2598–2614. https://doi.org/10.1109/TMI.2022.3167808.
53. Zhang, Y., Jin, Y., Chen, J., Kan, S., Cen, Y., and Cao, Q. (2020). PGAN: Part-Based Nondirect Coupling Embedded GAN for Person Reidentification. IEEE MultiMedia *27*, 23–33. https://doi.org/10.1109/MMUL.2020.2999445.
54. Deng, J., Pang, G., Zhang, Z., Pang, Z., Yang, H., and Yang, G. (2019). cGAN Based Facial Expression Recognition for Human-Robot Interaction. IEEE Access *7*, 9848–9859. https://doi.org/10.1109/ACCESS.2019.2891668.
55. Wu, C., Zhang, X., Zhang, Y., Hui, H., Wang, Y., and Xie, W. (2025). Towards generalist foundation model for radiology by leveraging web-scale 2D&3D medical data. Nat. Commun. *16*, 7866. https://doi.org/10.1038/s41467-025-62385-7.
56. Alkhaldi, A., Alnajim, R., Alabdullatef, L., Alyahya, R., Chen, J., Zhu, D., Alsinan, A., and Elhoseiny, M. (2024). MiniGPT-Med: Large Language Model as a General Interface for Radiology Diagnosis. Preprint at arXiv. https://doi.org/10.48550/arXiv.2407.04106.
57. Hu, E.J., Shen, Y., Wallis, P., Allen-Zhu, Z., Li, Y., Wang, S., Wang, L., and Chen, W. (2021). LoRA: Low-Rank Adaptation of Large Language Models. International Conference on Learning Representations. https://doi.org/10.48550/arXiv.2106.09685.
58. Rombach, R., Blattmann, A., Lorenz, D., Esser, P., and Ommer, B. (2022). High-Resolution Image Synthesis with Latent Diffusion Models. IEEE/CVF Conference on Computer Vision and Pattern Recognition (CVPR) (IEEE), pp. 10674–10685. https://doi.org/10.1109/CVPR52688.2022.01042.
59. He, K., Zhang, X., Ren, S., and Sun, J. (2016). Deep Residual Learning for Image Recognition. Conference on Computer Vision and Pattern Recognition (CVPR) (IEEE), pp. 770–778. https://doi.org/10.1109/CVPR.2016.90.
60. Baranchuk, D., Rubachev, I., Voynov, A., Khrulkov, V., and Babenko, A. (2022). Label-Efficient Semantic Segmentation with Diffusion Models. International Conference on Learning Representations. https://doi.org/10.48550/arXiv.2112.03126.
61. Selvaraju, R.R., Cogswell, M., Das, A., Vedantam, R., Parikh, D., and Batra, D. (2020). Grad-CAM: Visual Explanations from Deep Networks via Gradient-based Localization. Int. J. Comput. Vis. *128*, 336–359. https://doi.org/10.1007/s11263-019-01228-7.
62. Guoxun, Z., Zebin, G., Caohui, D., Jiaxin, L., Yuerong, L., Yaou, L., Qian, C., Ling, W., Kailun, F., Tianyun, W., et al. (2026). Code, datasets, and results for the paper "A multi-modal foundation model for brain disease diagnosis and medical imaging" (Zenodo). https://doi.org/10.5281/zenodo.7549620.

Patterns, Volume *7*

Supplemental information

A multi-modal foundation model

for brain disease diagnosis and medical imaging

Guoxun Zhang, Zebin Gao, Caohui Duan, Jiaxin Liu, Yuerong Lizhu, Yaou Liu, Qian Chen, Ling Wang, Kailun Fei, Tianyun Wang, YuJia Chen, Yanchen Guo, Feng Xu, Yuchen Guo, Xin Lou, and Qionghai Dai

A multi-modal foundation model for brain disease diagnosis and medical imaging

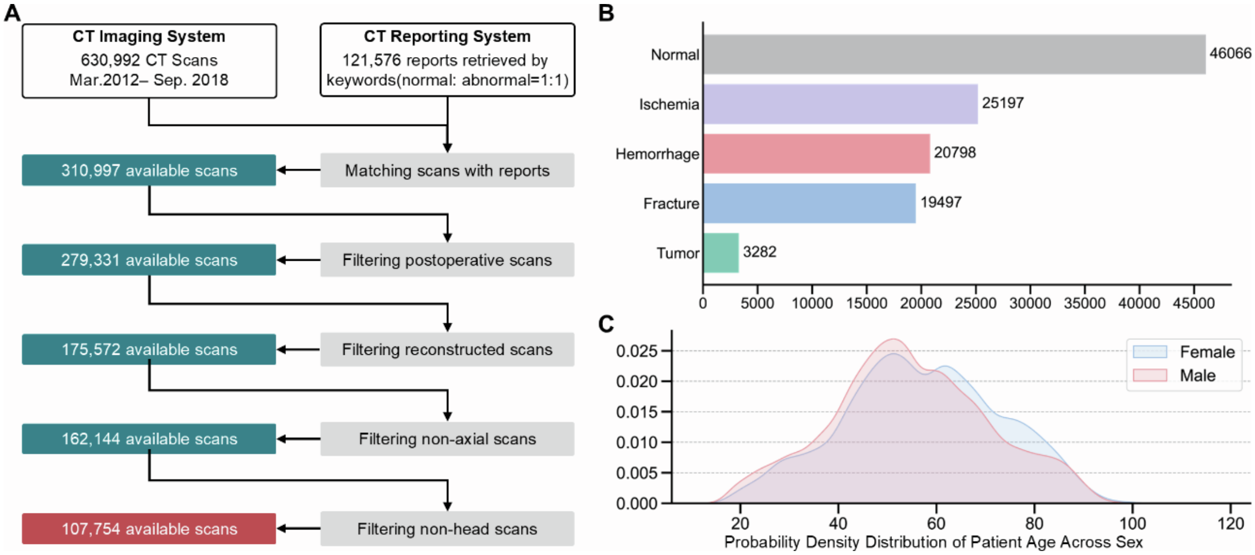

Fig. S1. Dataset overview of BrainCT-3M, used for pre-training Brainfound

(A) Data collection and preprocessing. A total of 630,992 brain CT scans and 121,576 associated diagnostic reports were collected as the source database. The data were subsequently screened using image quality criteria and a natural language processing (NLP) based report filtering procedure (Methods), culminating in the curated pre-training dataset BrainCT-3M. BrainCT-3M comprises 107,754 brain CT scans with matched reports, totaling over 3 million images.

(B) Distribution of CT studies across examination types in BrainCT-3M.

(C) Patient age distribution in BrainCT-3M.

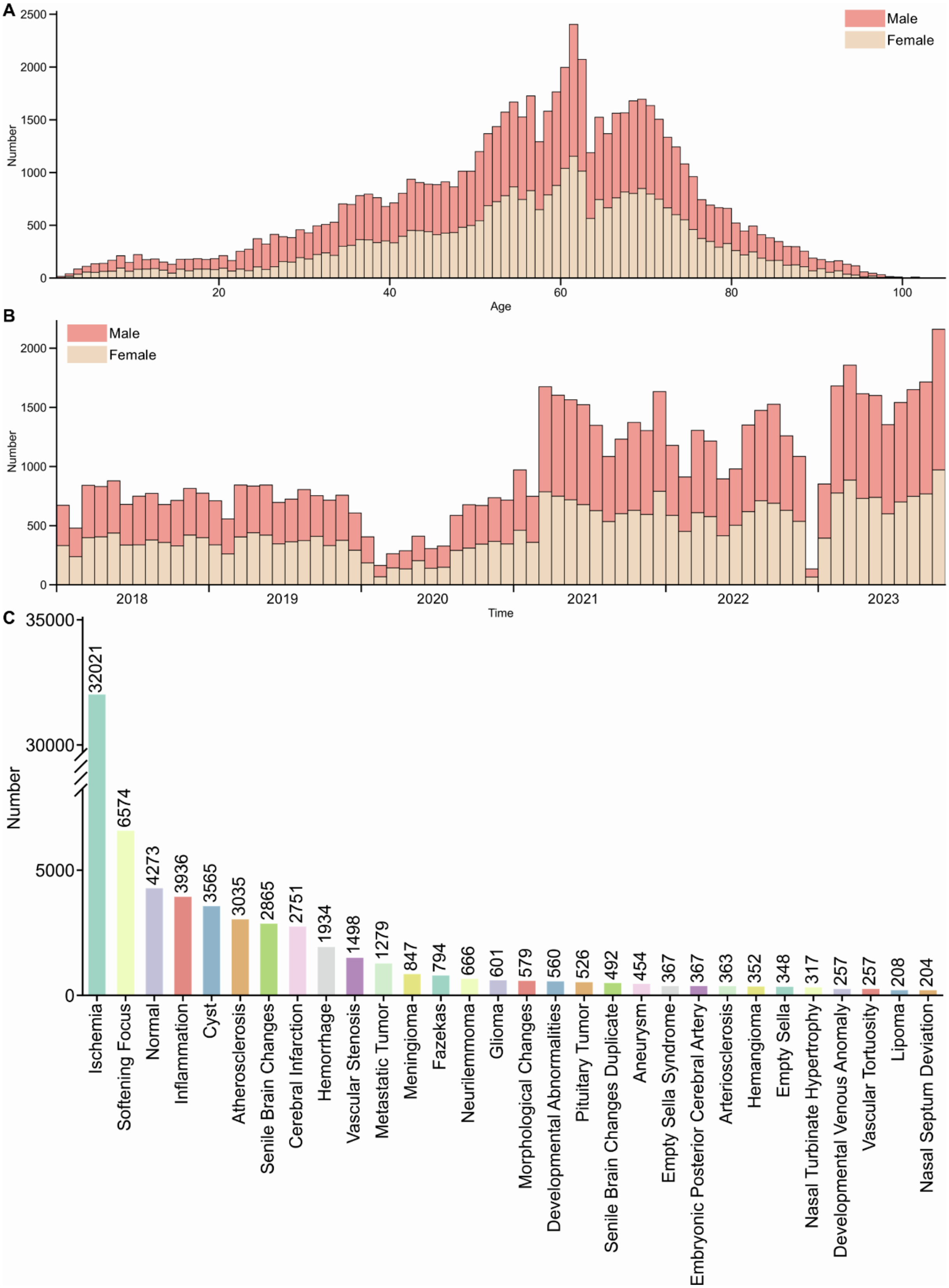

13 **Fig. S2. Dataset overview of BrainMRI-7M, used for pre-training Brainfound**

(A) Patient age distribution in BrainMRI-7M. BrainMRI-7M consists of brain MRI studies from 68,653 patients, totaling approximately 7 million images.

(B) Distribution of examination dates in BrainMRI-7M. The dataset spans a five-year period, from 2018 to 2023.

(C) Token and term-frequency statistics for the associated BrainMRI-7M reports. Report text was tokenized using an automated NLP pipeline. Among 68,653 MRI reports, the top 30 most frequent clinical terms are shown.

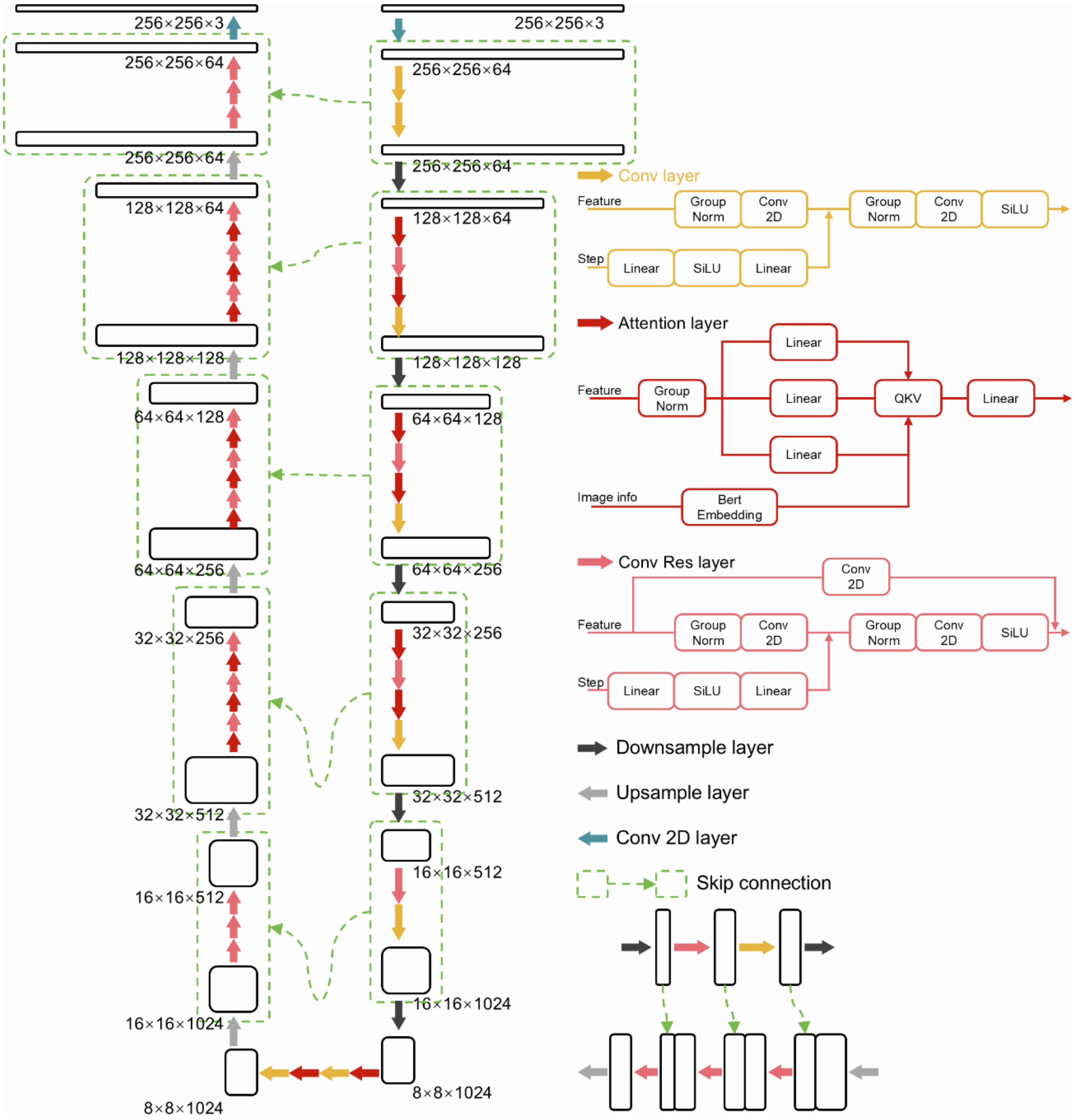

Fig. S3. Architecture of the visual module in Brainfound

The visual module adopts a U-shaped encoder–decoder architecture. Input images are progressively downsampled across encoder stages to aggregate multi-scale and global contextual features, and are subsequently upsampled in the decoder for image reconstruction. Textual conditioning is provided by a BERT-based text encoder, and image–text fusion is implemented via cross-attention layers that modulate the reconstruction process. The left panel shows the overall network architecture, and the right panel provides a legend explaining the icons used in the diagram.

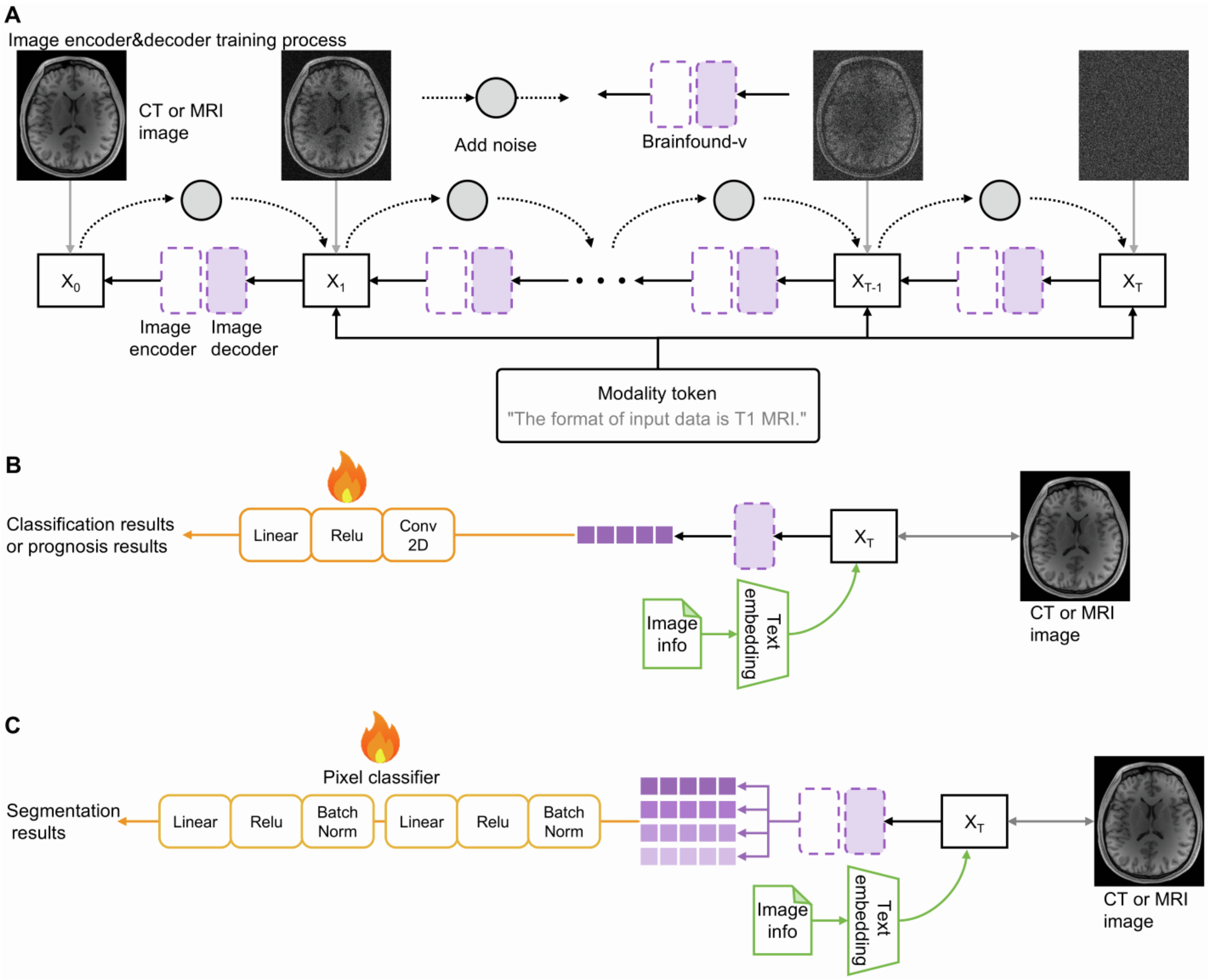

Fig. S4. Pre-training and task-specific fine-tuning of the Brainfound image encoder-decoder

(A) Pretraining of the image encoder–decoder following the DDPM framework. Clean brain CT or MRI images are progressively corrupted by additive noise to obtain noisy samples. The model is trained to denoise and reconstruct the original image from noise. A modality token (CT or MRI) is provided as a conditioning input to the visual network to guide reconstruction.

(B) Classification fine-tuning. Feature representations extracted by the pretrained Brainfound image encoder are fed into an MLP classifier to predict diagnostic labels. During downstream adaptation, the classifier head is fine-tuned using a limited amount of labeled data.

(C) Segmentation fine-tuning. Multi-scale features from the pretrained encoder－decoder are aggregated and passed to a pixel-wise classifier to produce lesion localization maps or segmentation masks. The pixel-wise classifier is fine-tuned with a limited amount of labeled data.

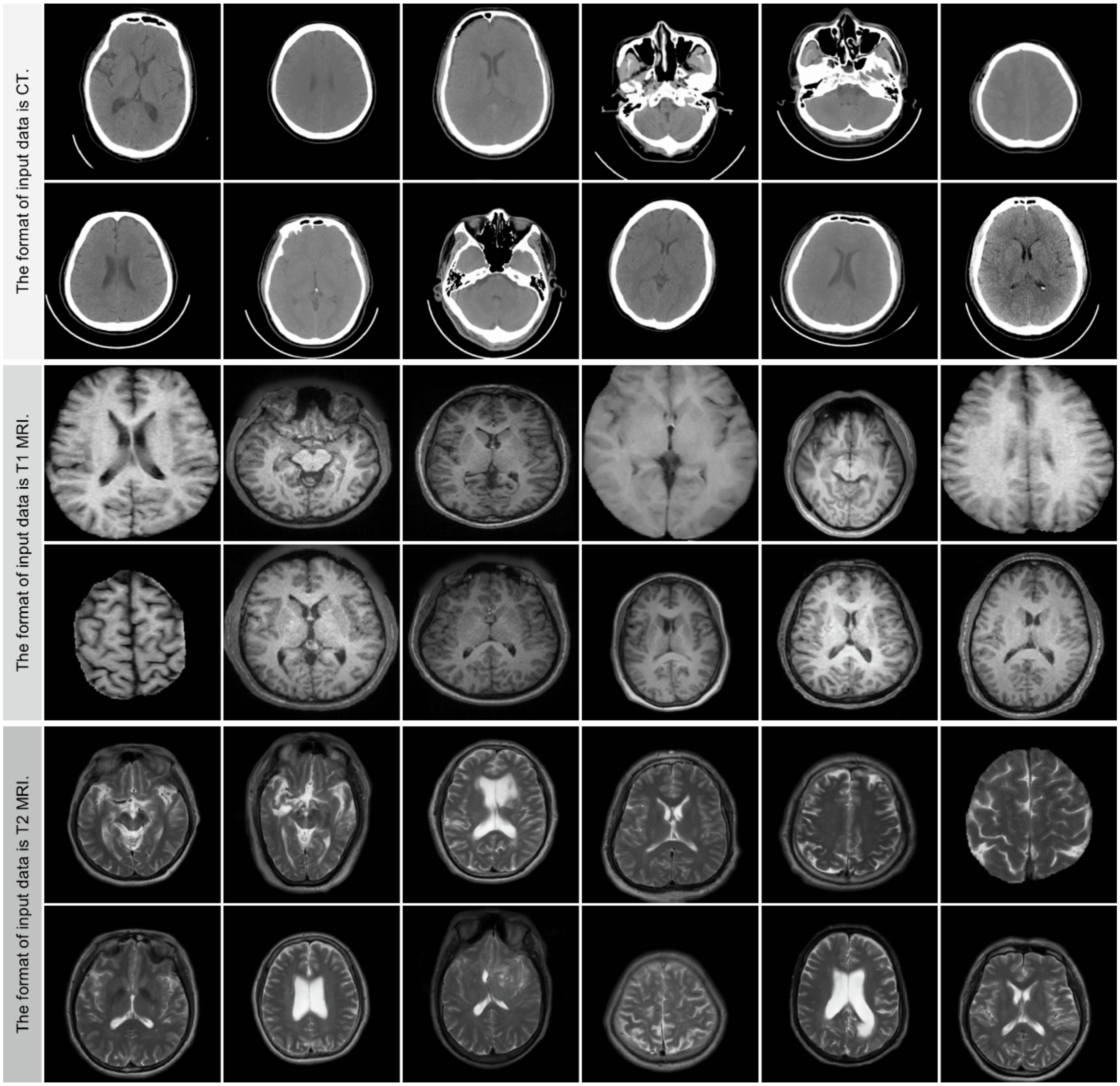

Fig. S5. Modality-conditioned image samples generated by the Brainfound vision module during diffusion pretraining

Representative generated samples are shown under modality conditioning. Brain CT, brain MRI T1WI, and brain T2WI are displayed in separate panels. Each modality panel contains 12 generated images.

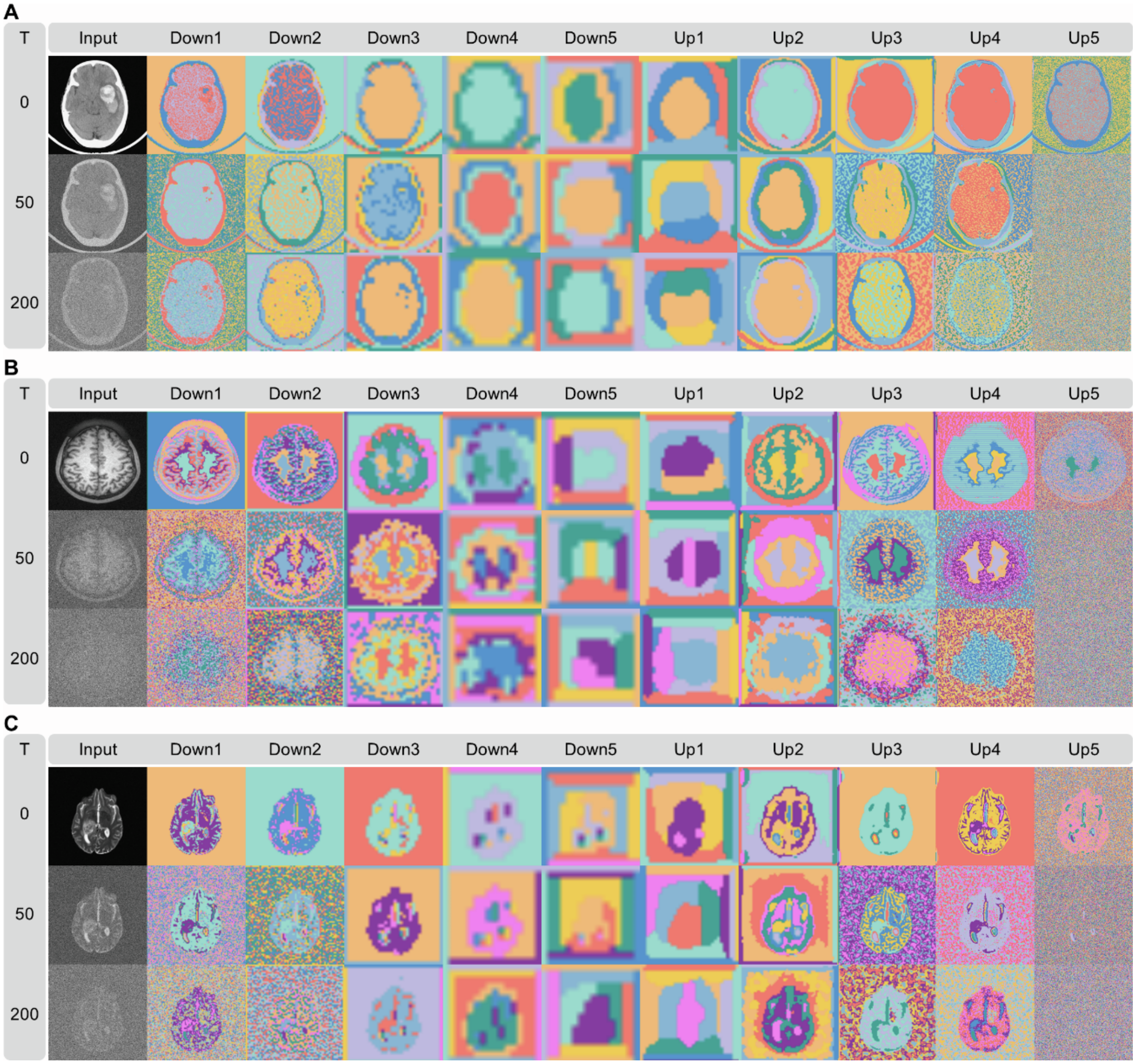

Fig. S6. Visualization of intermediate feature representations extracted by Brainfound

Feature maps are visualized from ten intermediate convolutional blocks of the diffusion U-shaped architecture, including five encoder stages (Down1 – Down5) and five decoder stages (Up1 – Up5), at three diffusion timesteps (T = 0, 50, 200). The first column shows the input images at the corresponding noise levels. Subsequent columns show k-means cluster assignment maps computed from the layer features (k = 10); each color denotes one cluster.

(A) using brain CT images as the input.

(B) using brain T1WI as the input.

(C) using brain T2WI as the input.

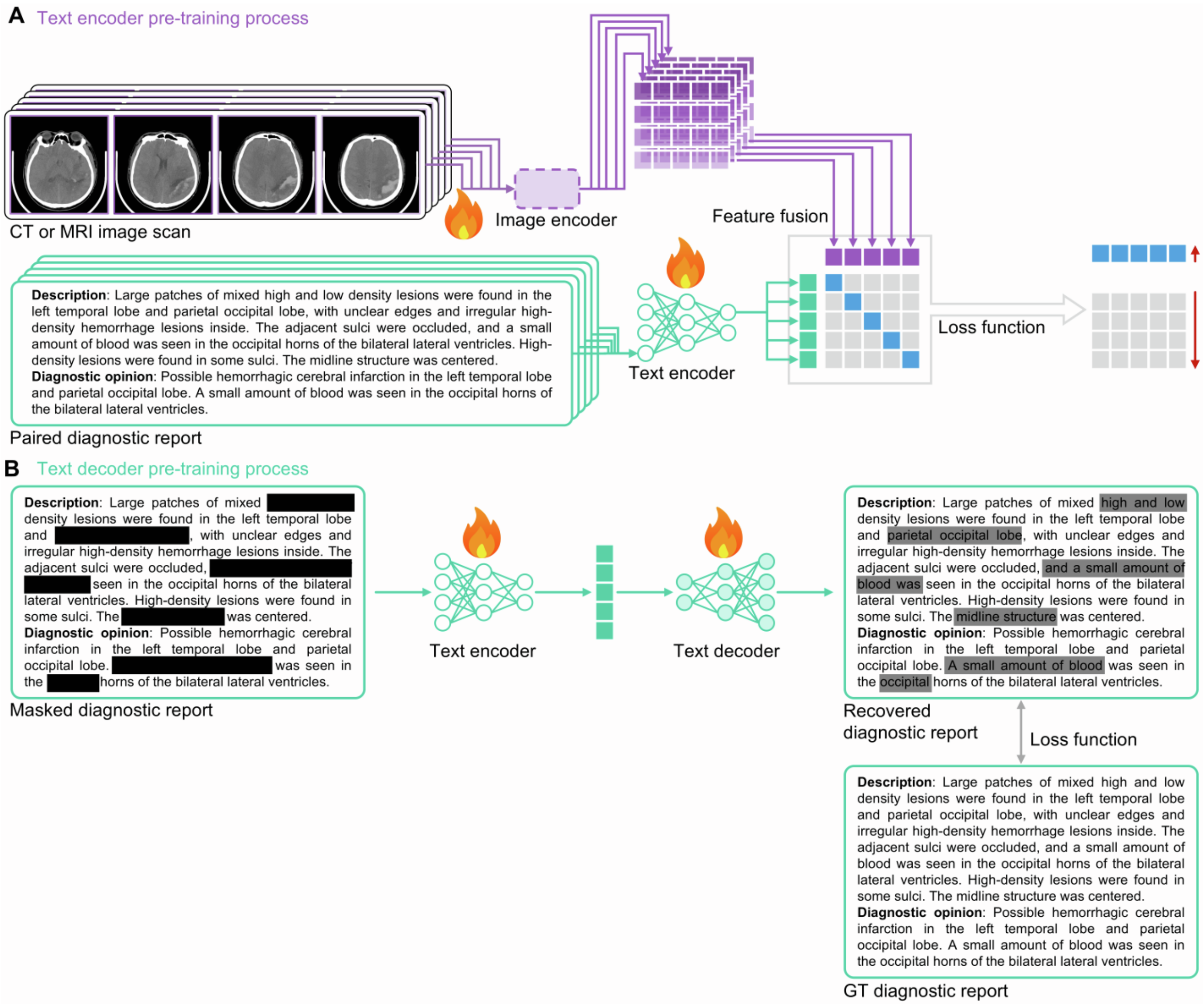

Fig. S7. Image-text alignment of Brainfound

(A) CLIP-style image–text alignment during pretraining. Brain imaging studies are encoded by the image encoder to obtain image embeddings, and the corresponding clinical reports are encoded by the text encoder to obtain text embeddings. The two latent space features calculate cosine similarity for contrastive learning as a loss function. The contrastive loss function is applied to increase the similarity of matched image–report pairs and decrease the similarity of mismatched pairs within a batch.

(B) Pre-training of the text decoder. Clinical reports are corrupted by phrase- or sentence-level masking and passed through the text encoder. The text decoder reconstructs the complete report, and the reconstruction loss is computed against the original report text. During this stage, the text encoder is kept fixed, and only the text decoder parameters are updated.

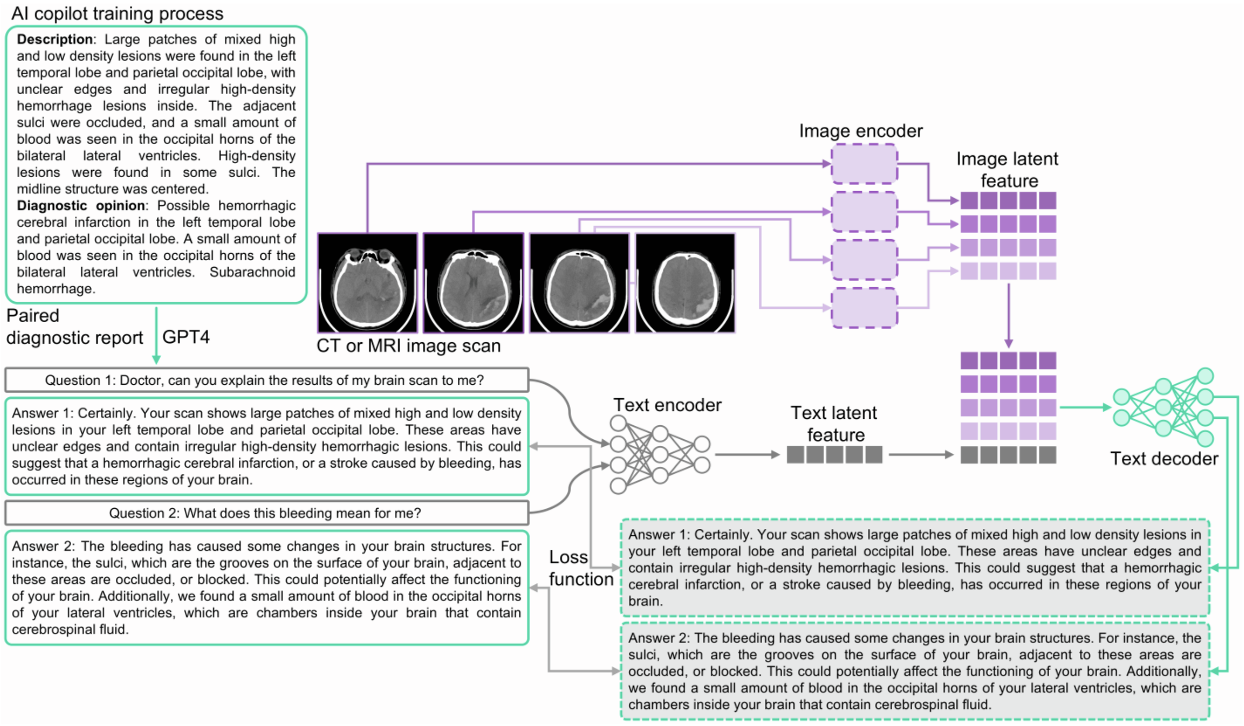

Fig. S8. Training pipeline for human-AI conversation in Brainfound

Given a diagnostic report, multi-turn open-ended dialogues are automatically generated using a large language model (GPT-4) to construct instruction–response pairs under diverse prompting templates. During training, user questions are encoded by the text encoder to produce text embeddings, and the corresponding imaging studies are encoded by the image encoder to produce image embeddings. The fused multimodal representation is provided to the text decoder, which predicts the response. The output of the text decoder and the answers during the conversation are used to compute the loss function, which subsequently guides the optimization of both the text encoder and decoder.

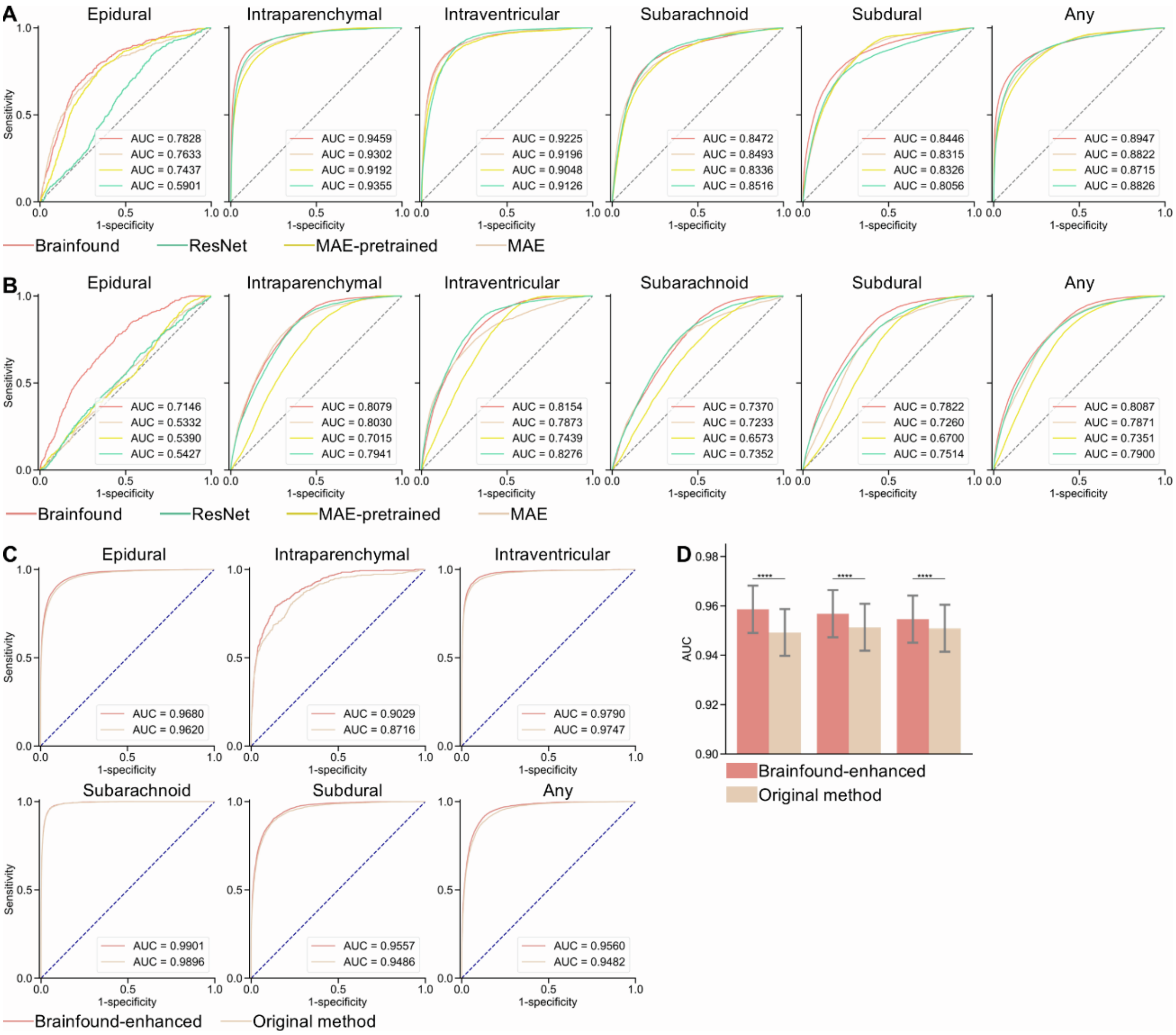

Fig. S9. Brain hemorrhage classification performance on the RSNA dataset

(A) ROC curves for six hemorrhage categories: Epidural, Intraparenchymal, Intraventricular, Subarachnoid, Subdural, and Any, after full-parameter fine-tuning of Brainfound and baseline models (ResNet, MAE-pretrained, and MAE). MAE-pretrained denotes an MAE model pretrained on BrainCT-3M, whereas MAE denotes an MAE model pretrained on a large-scale natural-image dataset.

(B) ROC curves for the same categories under a frozen-backbone setting, where the feature extractor is kept fixed, and only the classification head is fine-tuned for Brainfound and baselines. Other settings are identical to panel (A).

(C) ROC curve obtained by replacing the backbone in a high-performing RSNA competition pipeline with the pretrained Brainfound backbone.

(D) Summary results corresponding to panel (C). Each experiment was repeated three times with different random seeds, and performance is reported across runs.

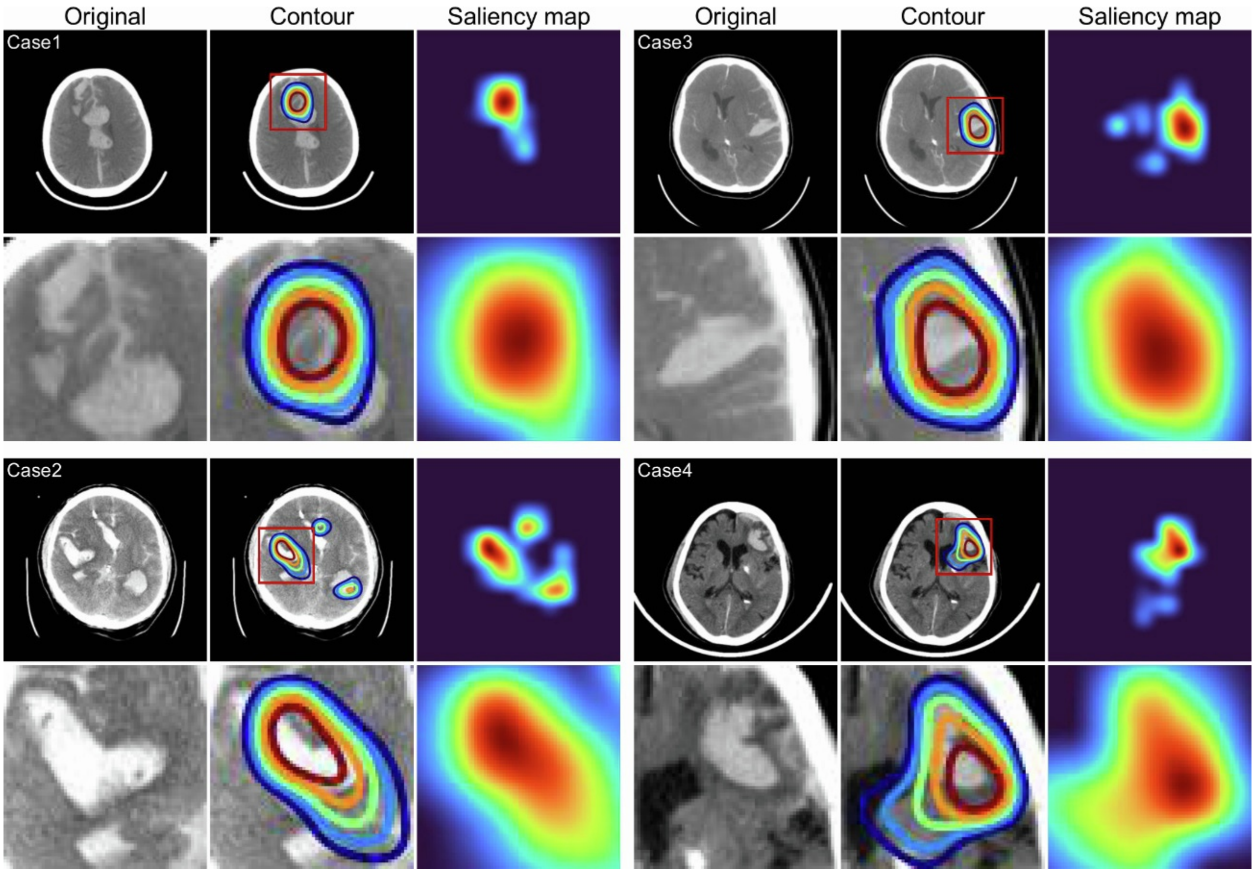

Fig. S10. Saliency maps produced by Brainfound for cerebral hemorrhage classification on the RSNA dataset

Representative saliency visualizations are shown for four brain CT images. For each example, the original CT image is shown in columns 1 and 4, the corresponding saliency contours are shown in columns 2 and 5, and the saliency heatmaps produced by Brainfound are shown in columns 3 and 6. Rows 2 and 4 provide enlarged views of the regions indicated by red boxes in rows 1 and 3, respectively.

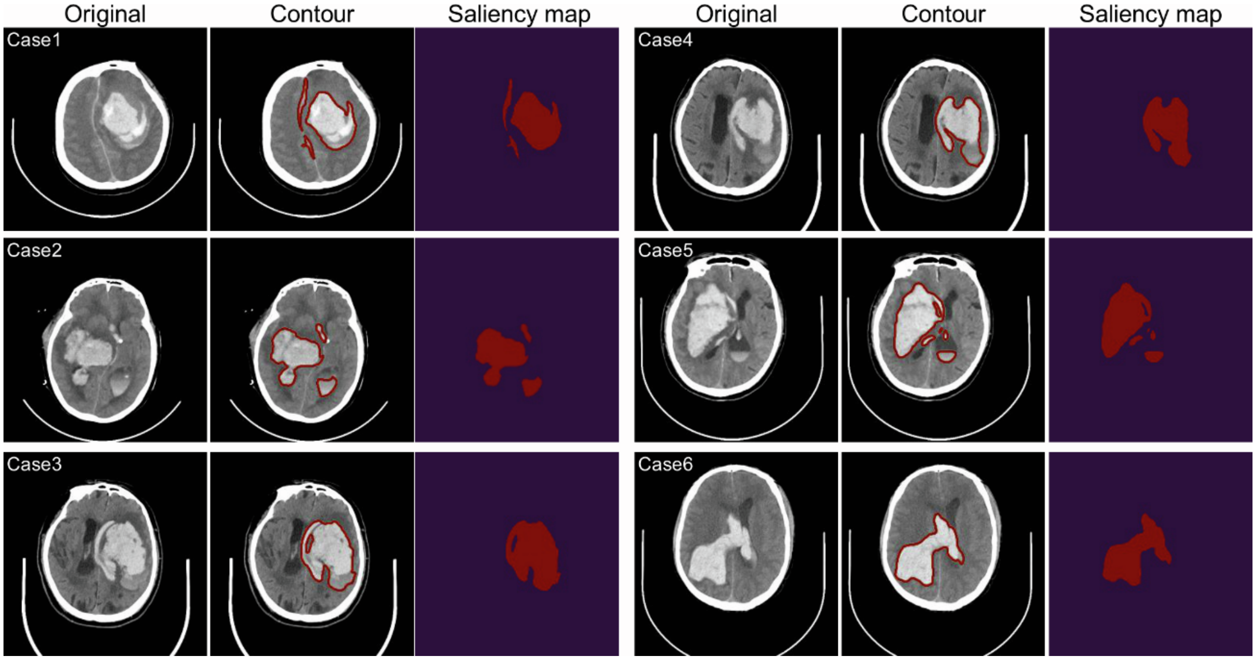

Fig. S11. Saliency maps generated by Brainfound for cerebral hemorrhage segmentation on the RSNA dataset

Representative saliency visualizations are shown for six brain CT images. For each example, the original CT image is shown in columns 1 and 4, the corresponding saliency outlines are shown in columns 2 and 5, and the saliency heatmaps generated by Brainfound are shown in columns 3 and 6.

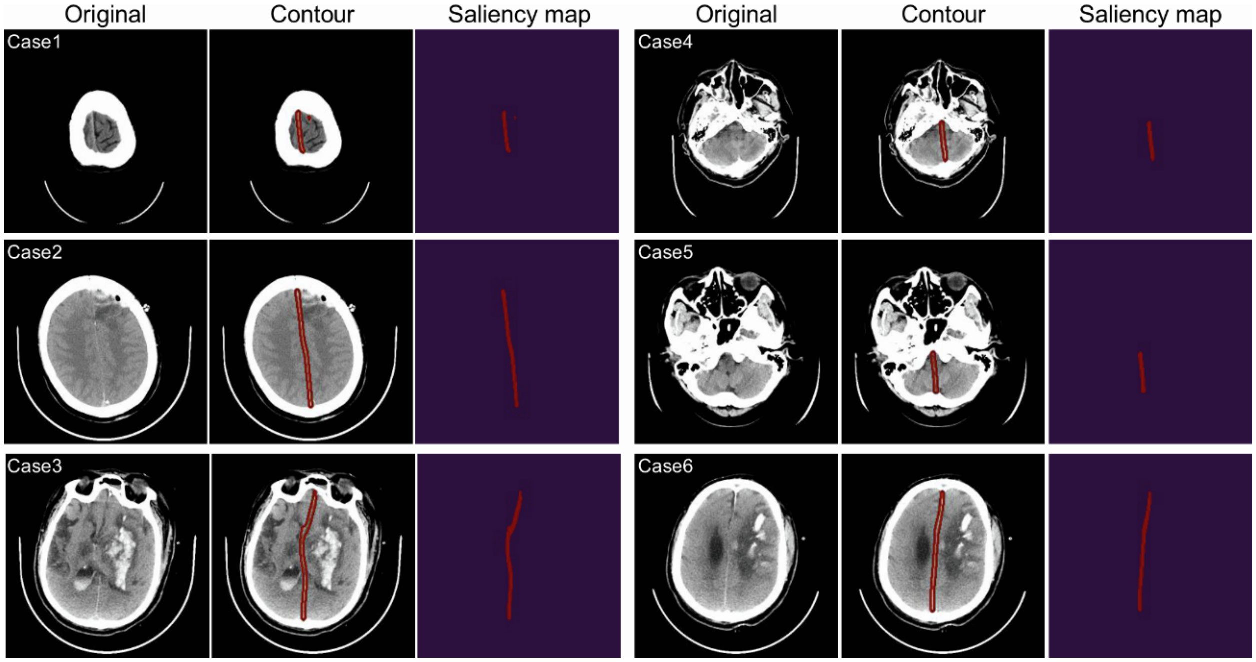

Fig. S12. Saliency maps generated by Brainfound for midline segmentation

Representative saliency visualizations are shown for six brain CT examples. For each example, the original CT image is shown in columns 1 and 4, the corresponding saliency outlines are shown in columns 2 and 5, and the saliency heatmaps generated by Brainfound are shown in columns 3 and 6.

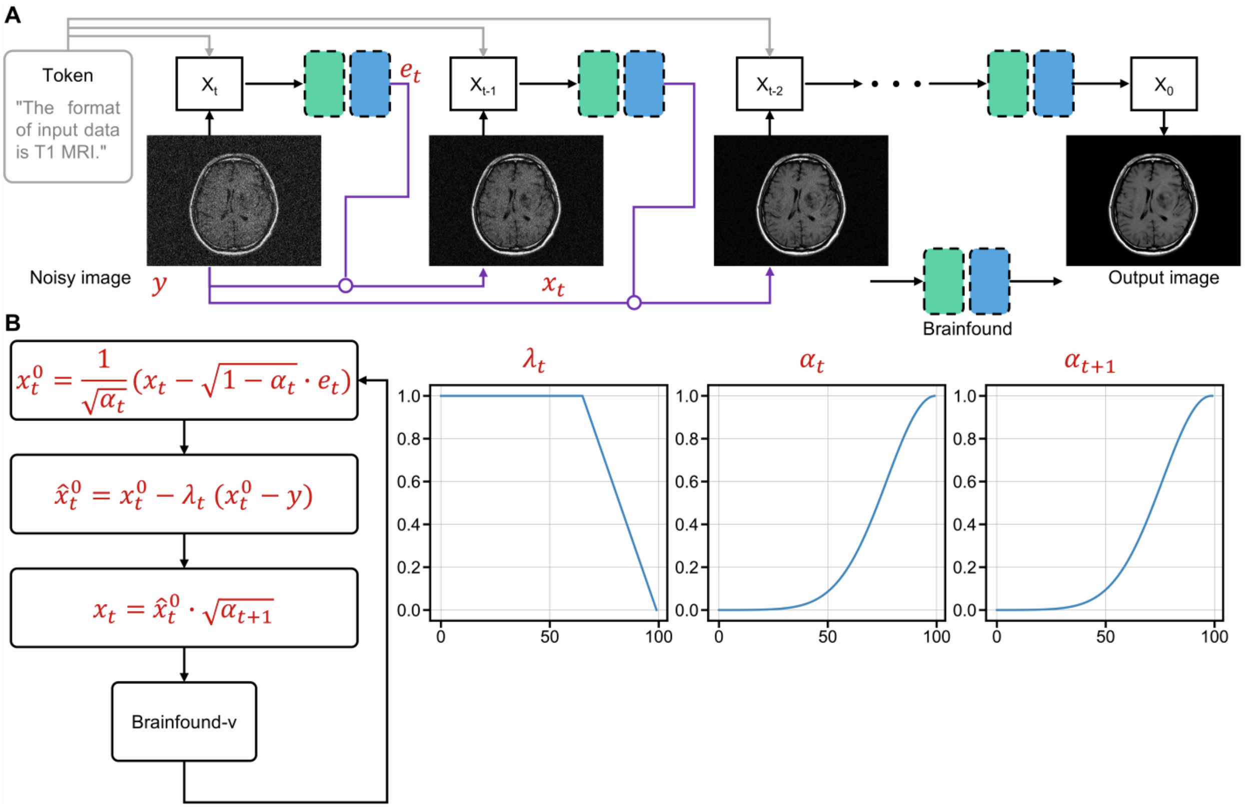

98 **Fig. S13. Zero-shot denoising procedure of Brainfound**

(A) Schematic illustration of the iterative denoising process. A noisy input image is incorporated into the DDPM based restoration procedure through repeated refinement steps, yielding a denoised output image. (B) Computation details for the iterative updates. The three curves in the right panel show the values of selected hyperparameters across iterations.

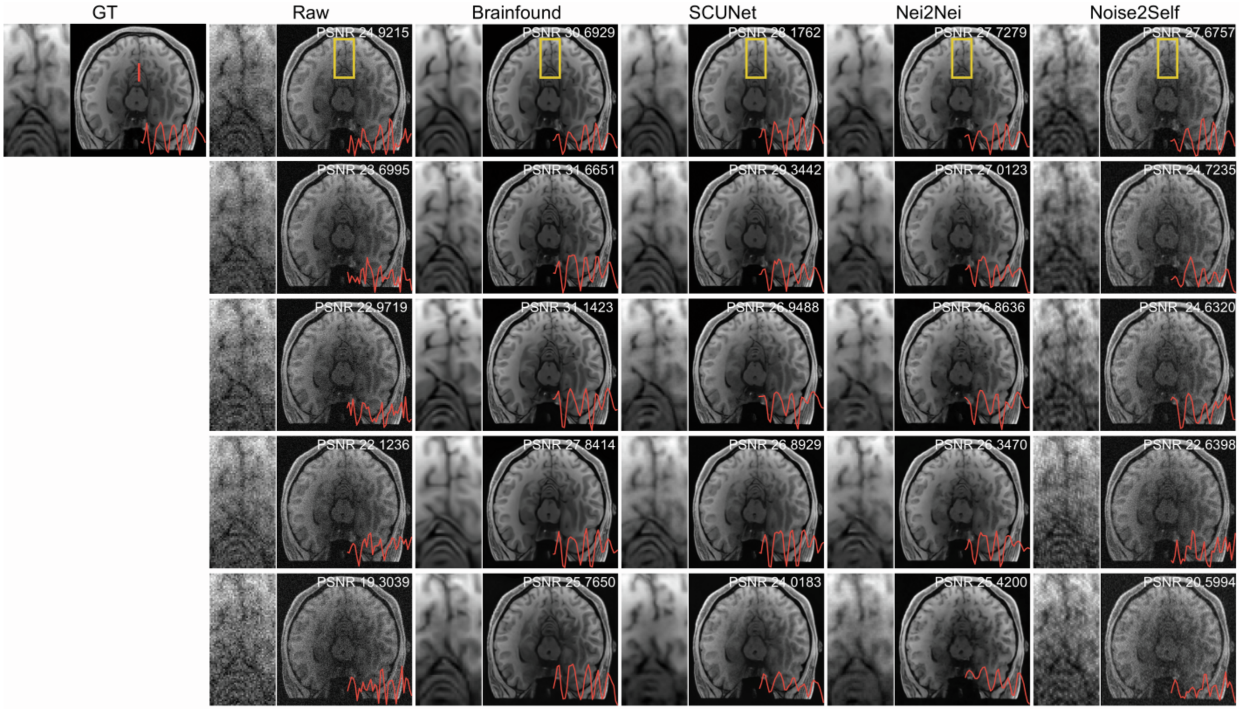

Fig. S14. Comparison of the denoising performance on 3T MRI images with simulated noise

Columns from left to right show the clean reference image, the noisy input image, the output of Brainfound, the output of SCUNnet, the output of Nei2Nei, and the output of Noise2Self. Rows 1 to 5 correspond to progressively increasing noise levels. The PSNR for each image is shown in the upper left corner. The region indicated by the yellow box is enlarged for visualization. Intensity profiles along the red line are shown for comparison.

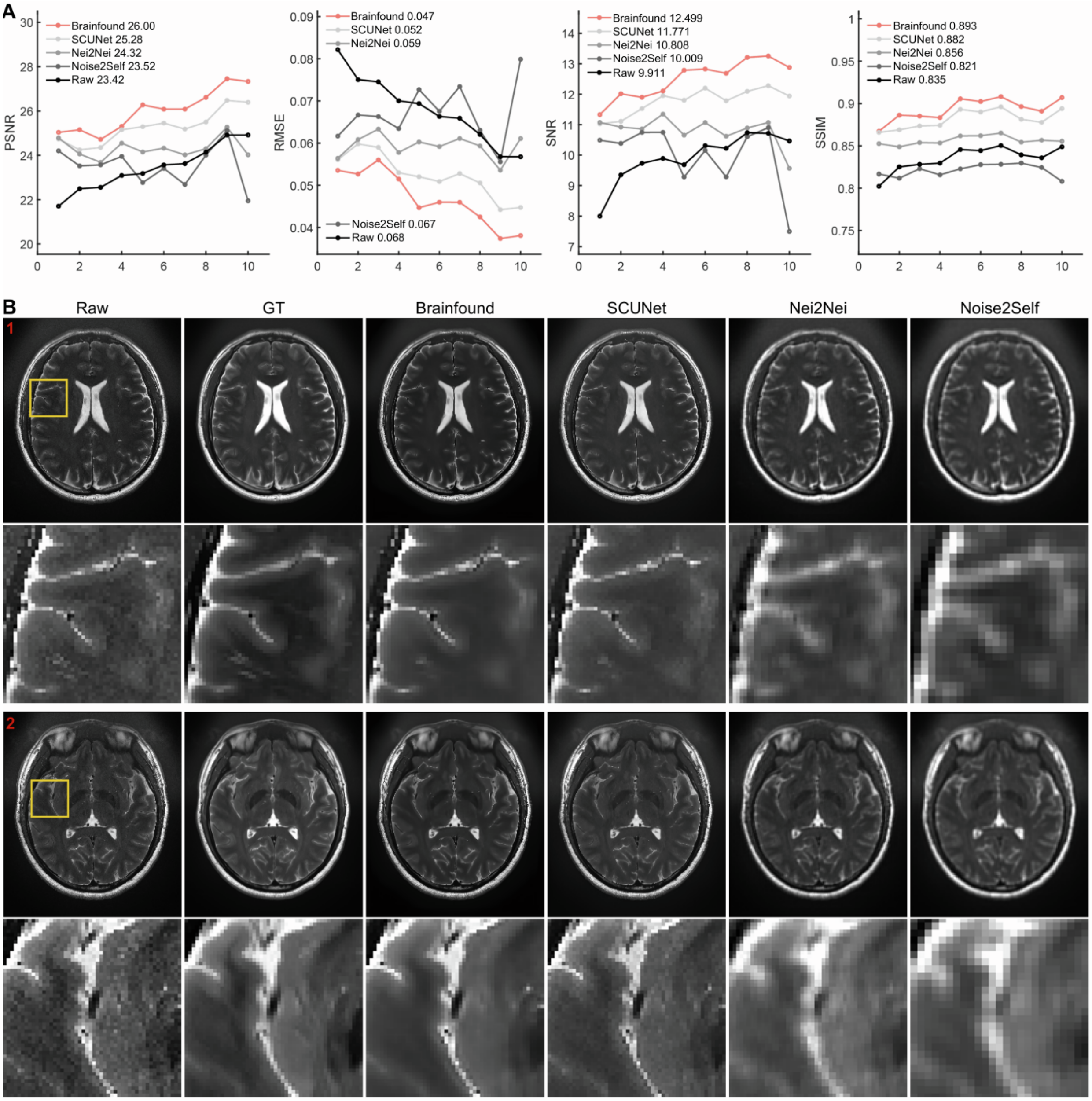

Fig. S15. The enhancement on 5T MRI T2 weighted images using Brainfound

(A) Quantitative comparison of Brainfound, SCUNet, Nei2Nei, and Noise2Self for MRI image enhancement on the test set captured by 5T MRI (n=10) at Beijing Friendship Hospital. Metrics are reported as PSNR, RMSE, SNR, and SSIM.

(B) Representative examples of 5T T2 weighted image denoising. Columns from left to right show the low SNR input, the high SNR reference, and the outputs of Brainfound, SCUNet, Nei2Nei, and Noise2Self. Regions indicated by yellow boxes in rows 1 and 3 are enlarged for visualization.

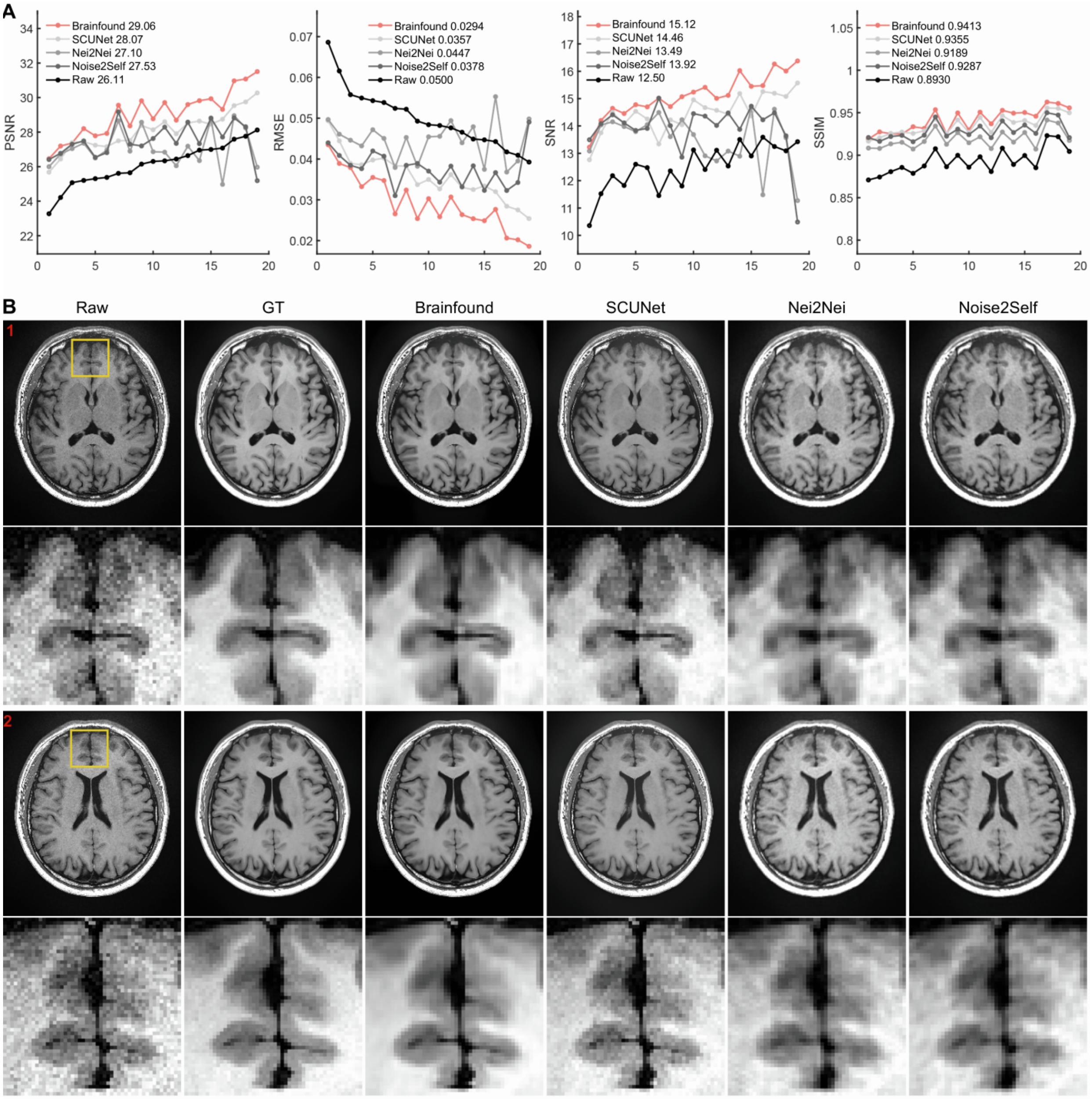

Fig. S16. The enhancement on 5T MRI T1 weighted images using Brainfound

(A) Quantitative comparison of Brainfound, SCUNet, Nei2Nei, and Noise2Self for MRI image enhancement on the external test dataset (n=19) captured by 5T MRI at Beijing Friendship Hospital. Metrics are reported as PSNR, RMSE, SNR, and SSIM.

(B) Representative examples of 5T T1 weighted image denoising. Columns from left to right show the low SNR input, the high SNR reference, and the outputs of Brainfound, SCUNet, Nei2Nei, and Noise2Self. Regions indicated by yellow boxes in rows 1 and 3 are enlarged for visualization.

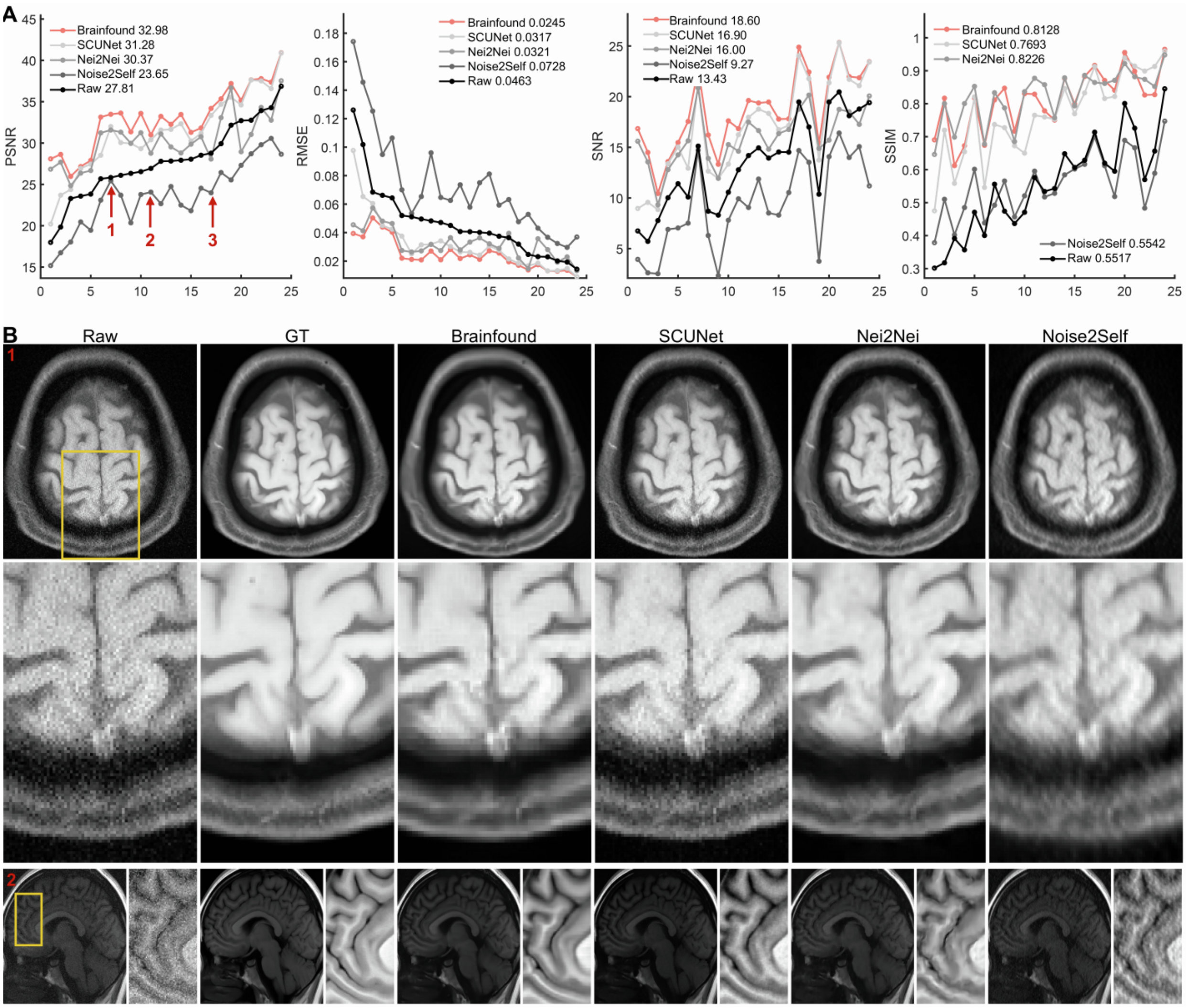

Fig. S17. The enhancement on real world 5T MRI images from an external center

(A) Quantitative comparison of Brainfound, SCUNet, Nei2Nei, and Noise2Self for MRI image enhancement on the external test dataset (n=25) captured at the Shanghai United Imaging center. Metrics are reported as PSNR, RMSE, SNR, and SSIM.

(B) Representative examples corresponding to the two cases indicated by arrows in panel (A). Displayed from left to right are the original noisy image, the high SNR GT image, the image enhanced by Brainfound, the image enhanced by SCUNet, the image enhanced by Nei2Nei, and the image enhanced by Noise2Self. The region outlined by the yellow box is enlarged for visualization.

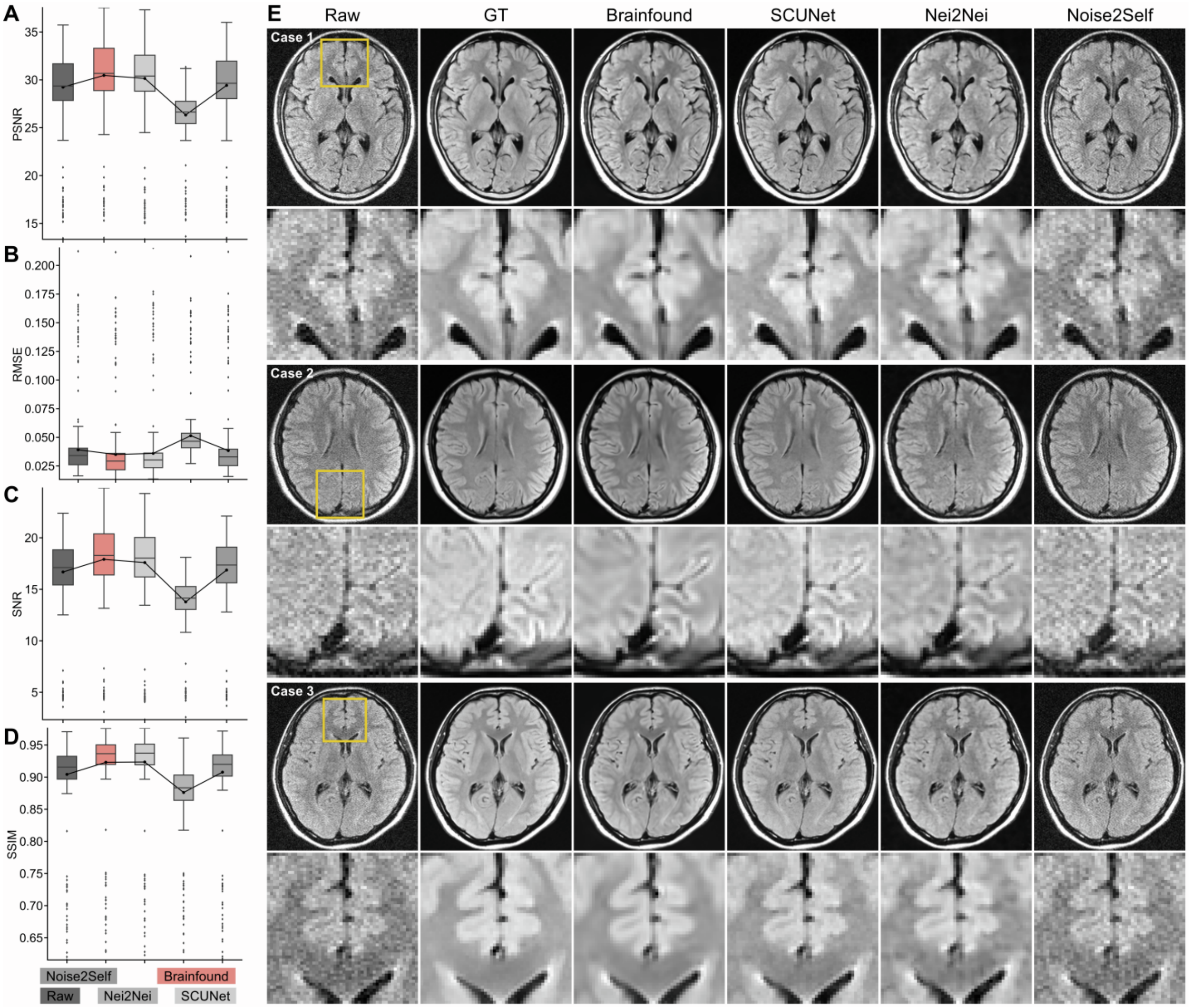

Fig. S18. Comparison of zero-shot enhancement on 0.3T FLAIR images

(A-D) Quantitative comparison of image enhancement results using four methods: Brainfound, Noise2Self, Nei2Nei, SCUNet on the 0.3T FLAIR dataset (with n=450). PSNR, RMSE, SNR, and SSIM were evaluated, respectively. Brainfound achieved the best scores in all metrics except SSIM.

(E) Representative denoising examples. From left to right: the original image, high SNR reference image, Brainfound-enhanced image, SCUNet-enhanced image, Nei2Nei-enhanced image, and Noise2Self-enhanced image. The region indicated by the yellow box is enlarged for visualization.

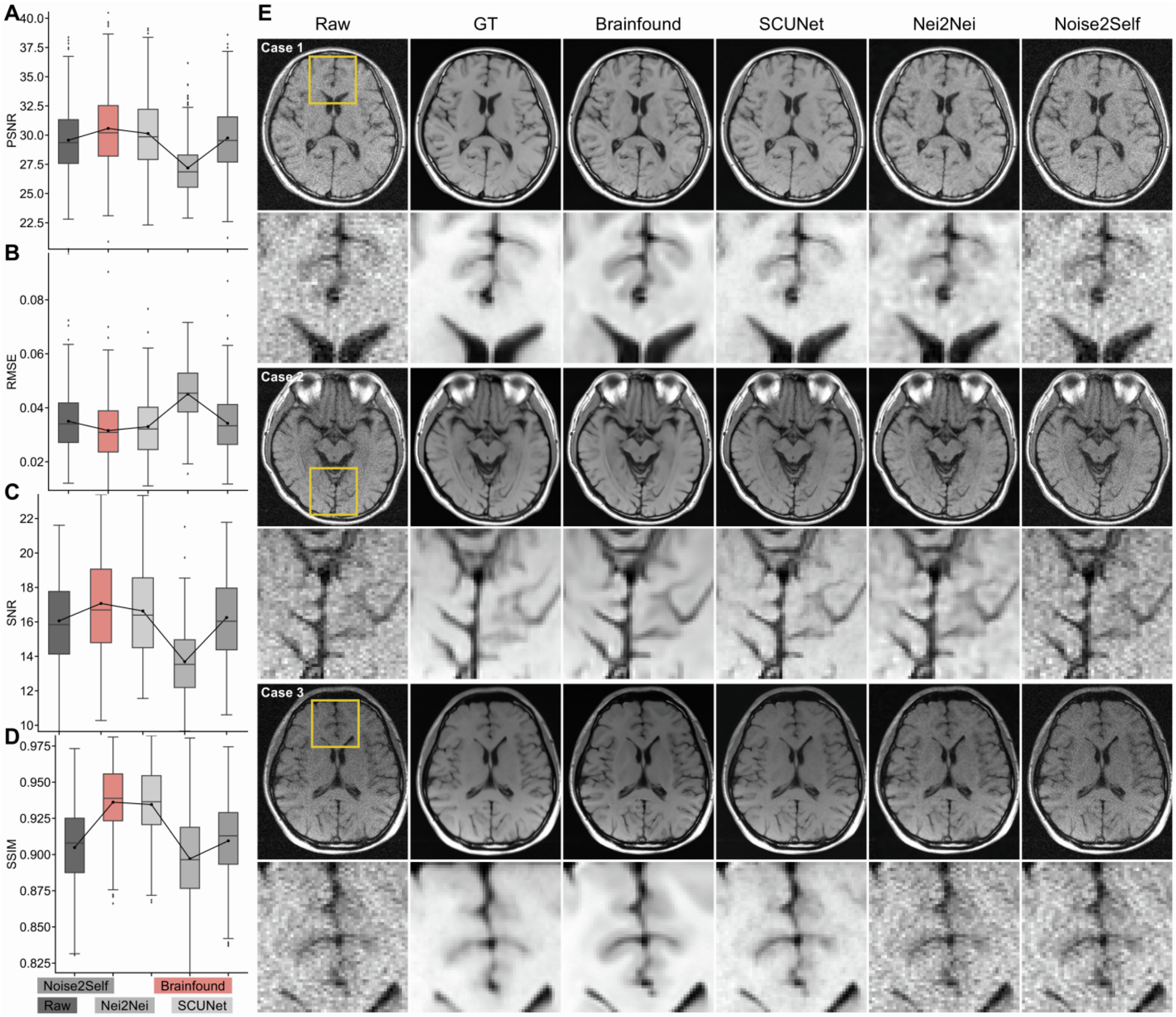

Fig. S19. Comparison of zero-shot enhancement on 0.3T T1WI

(A-D) Quantitative comparison of image enhancement results using four methods: Brainfound, Noise2Self, Nei2Nei, SCUNet on the 0.3T T1 weighted dataset (with n=450). PSNR, RMSE, SNR, and SSIM were respectively computed. Brainfound achieved the best scores in all metrics.

(E) Representative denoising examples. From left to right: the original image, high SNR reference image, Brainfound-enhanced image, SCUNet-enhanced image, Nei2Nei-enhanced image, and Noise2Self-enhanced image. The yellow-boxed area is enlarged for display.

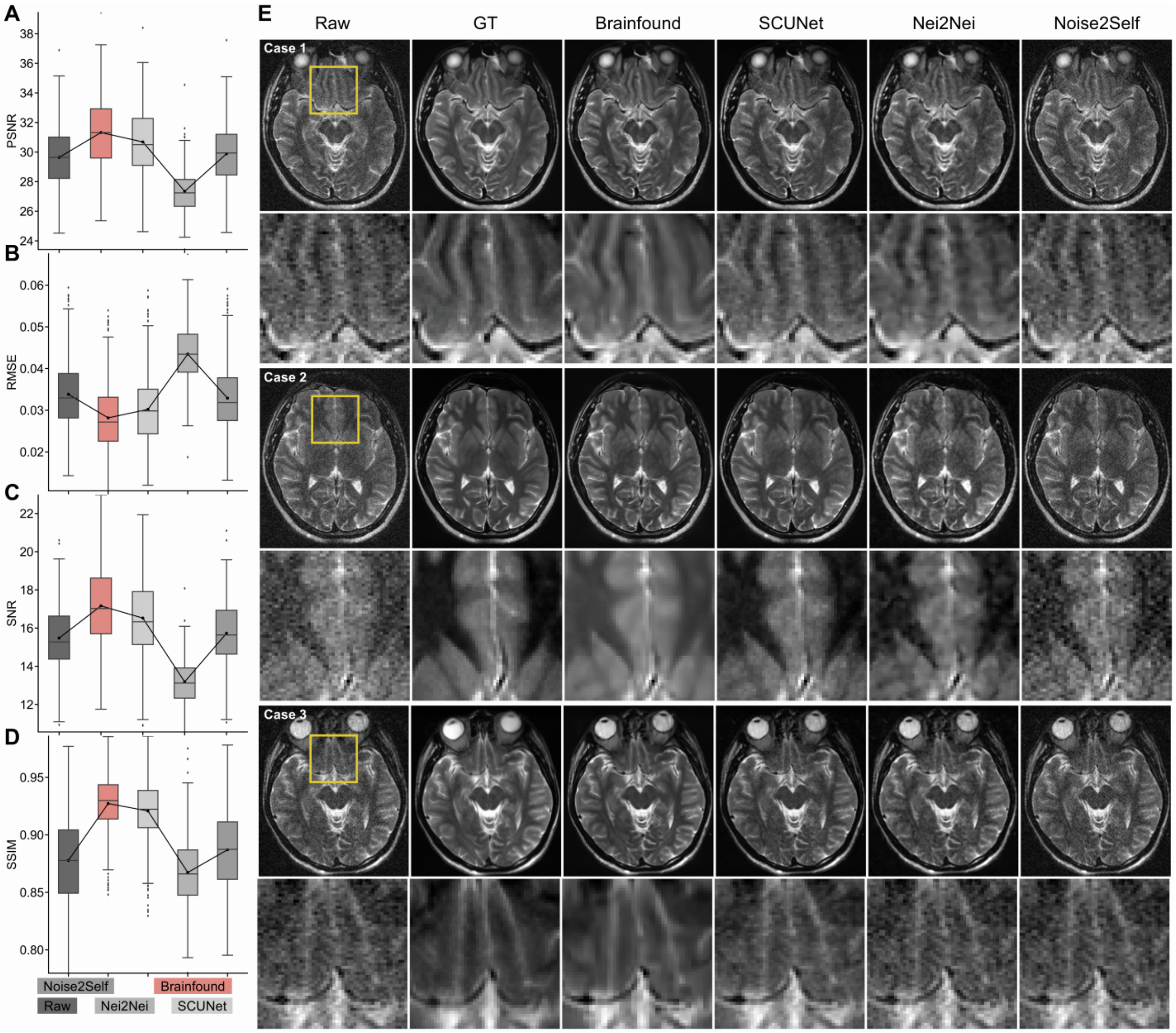

Fig. S20. Comparison of zero-shot enhancement on 0.3T T2WI

(A-D) Quantitative comparison of image enhancement results using four methods: Brainfound, Noise2Self, Nei2Nei, SCUNet (with n=450). PSNR, RMSE, SNR, and SSIM were respectively calculated. Brainfound achieved the best scores in all metrics.

(E) Representative denoising examples. From left to right: the original image, high SNR reference image, Brainfound-enhanced image, SCUNet-enhanced image, Nei2Nei-enhanced image, and Noise2Self-enhanced image. The yellow-boxed area is enlarged for display.

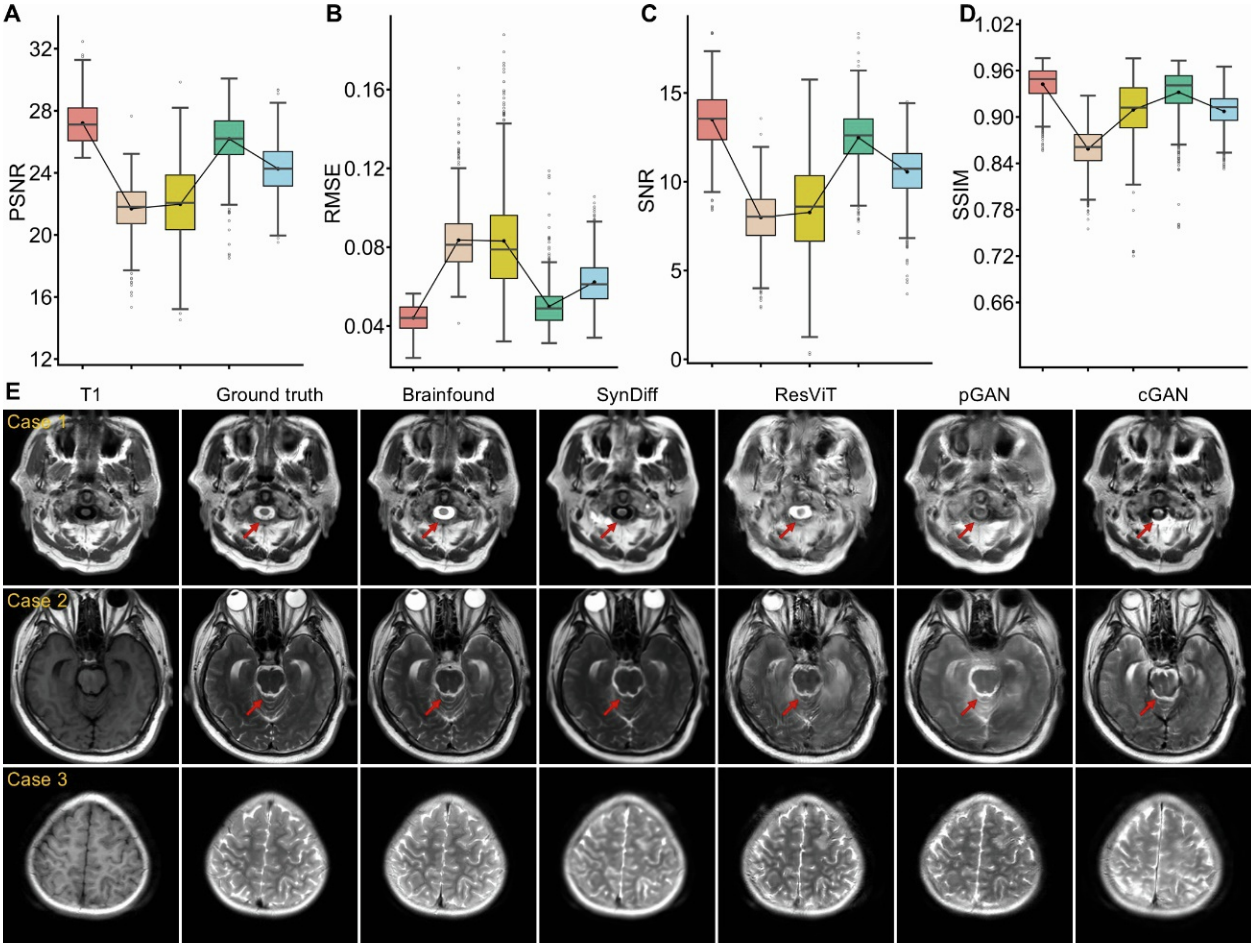

Fig. S21. Performance of Brainfound for MRI modality translation from T1WI to T2WI

(A-D) Quantitative evaluation of image modality translation with Brainfound, SynDiff, ResViT, pGAN, and cGAN (n=1936). Metrics include PSNR, RMSE, SNR, and SSIM.

(E) Representative T1 to T2 translation examples. From left to right: original T1WI, paired T2WI, results from Brainfound, SynDiff, ResViT, pGAN, and cGAN. In case 1, Brainfound accurately identifies the cerebrospinal fluid (CSF) around the spinal cord that shows low signal intensity on T1WI and converts these areas into high signal intensity on generated T2WI. In case 2, Brainfound generated a clearer image in which the pons and their surrounding structures look sharp and have good contrast. In case 3, Brainfound achieves a higher resolution in conversion tasks, while other methods result in slightly blurry images.

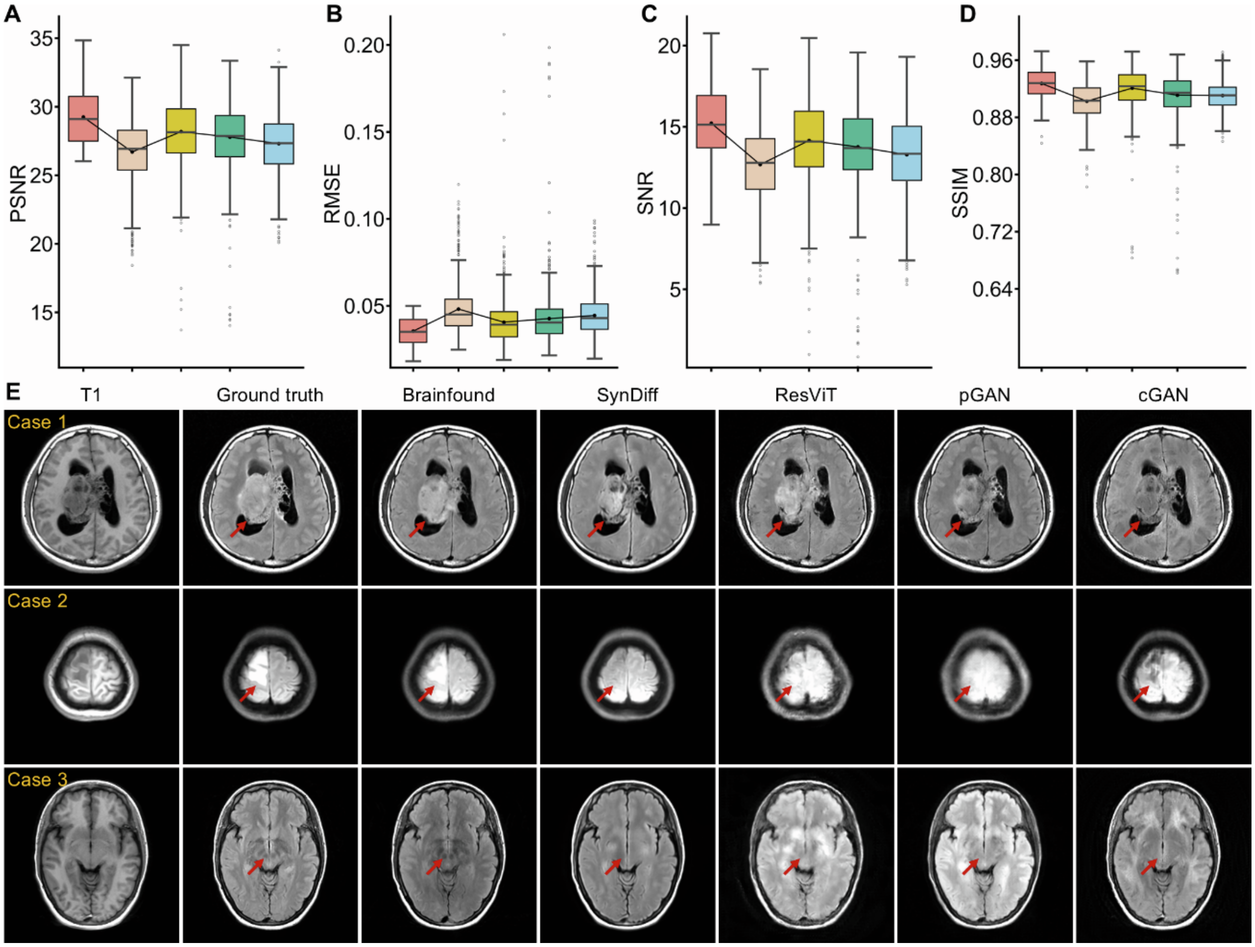

Fig. S22. Performance of Brainfound for MRI modality translation from T1WI to FLAIR

(A-D) Image modality translation was assessed quantitatively with Brainfound, SynDiff, ResViT, pGAN, and cGAN (n=1936). Brainfound showed superior performance across all metrics, including PSNR, RMSE, SNR, and SSIM.

(E) Three cases of T1WI-to-FLAIR image translation via five methods. In case 1, Brainfound more effectively transformed the situation of the tumor. In case 2, Brainfound accurately converted the edema lesion with low signal intensity on T1WI into high signal intensity on FLAIR. In case 3, Brainfound accurately generated the distinct darkened areas of the red nucleus and substantia nigra on the FLAIR image.

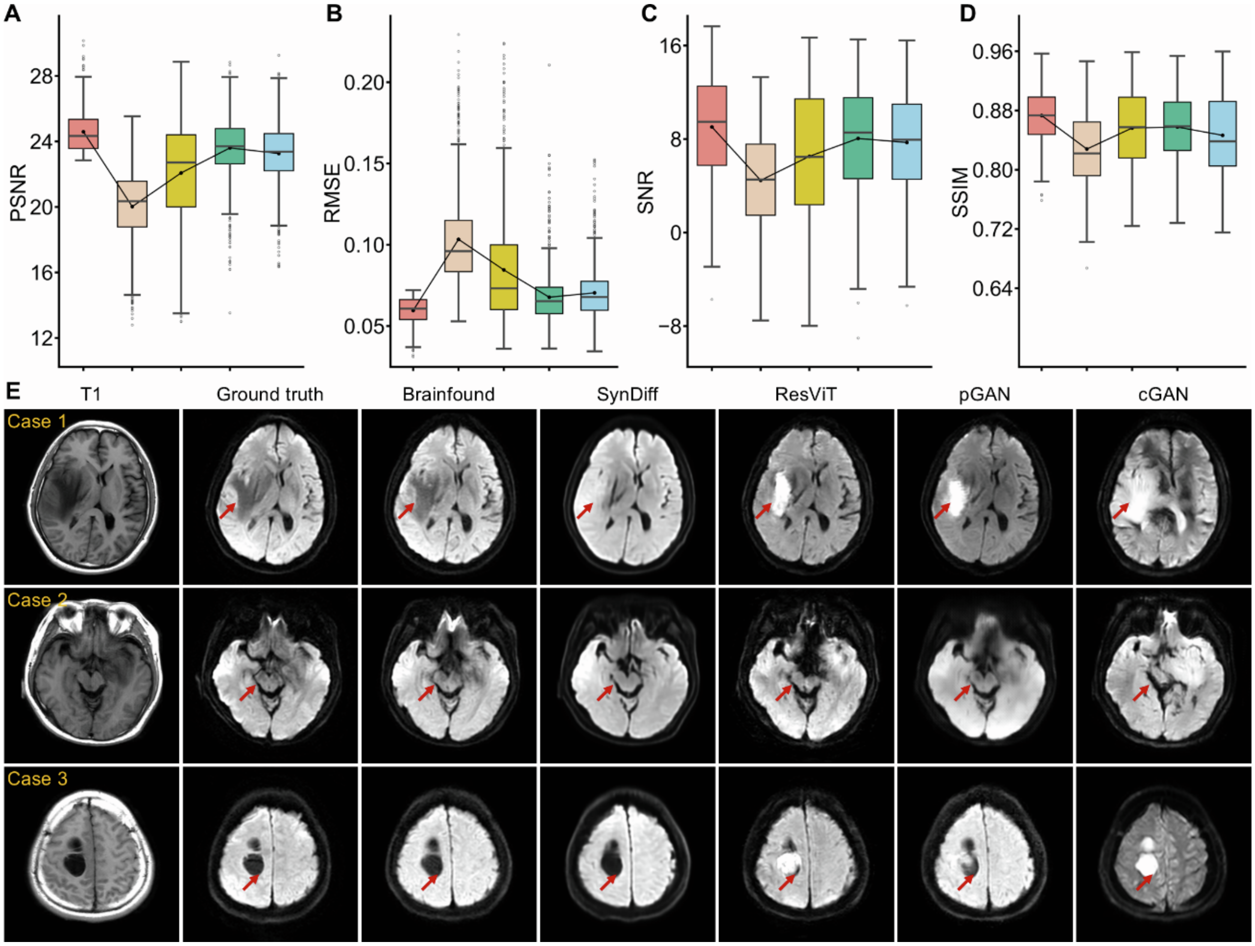

Fig. S23. Performance of Brainfound for MRI modality translation from T1WI to standard-b-value DWI

(A-D) Quantitative assessment of T1WI to standard-b-value DWI image translation results was carried out using five methods: Brainfound, SynDiff, ResViT, pGAN, and cGAN (n=1936). The metrics PSNR, RMSE, SNR, and SSIM were calculated, with Brainfound obtaining the best performance in all metrics.

(E) Three cases of T1WI to standard-b-value DWI Image translation via five methods. For case 1 and case 3, the peritumoral vasogenic edema and the cystic lesions typically do not show restricted diffusion (high signal) on DWI images, and Brainfound accurately identified the edema and cystic regions and output the images with corresponding hypointense lesions. In case 2, the images generated by Brainfound exhibit less distortion in the slices near the base of the skull, and the depiction of the brainstem, ambient cisterns, and medial temporal lobes is clearer and matches the ground truth better than the other models.

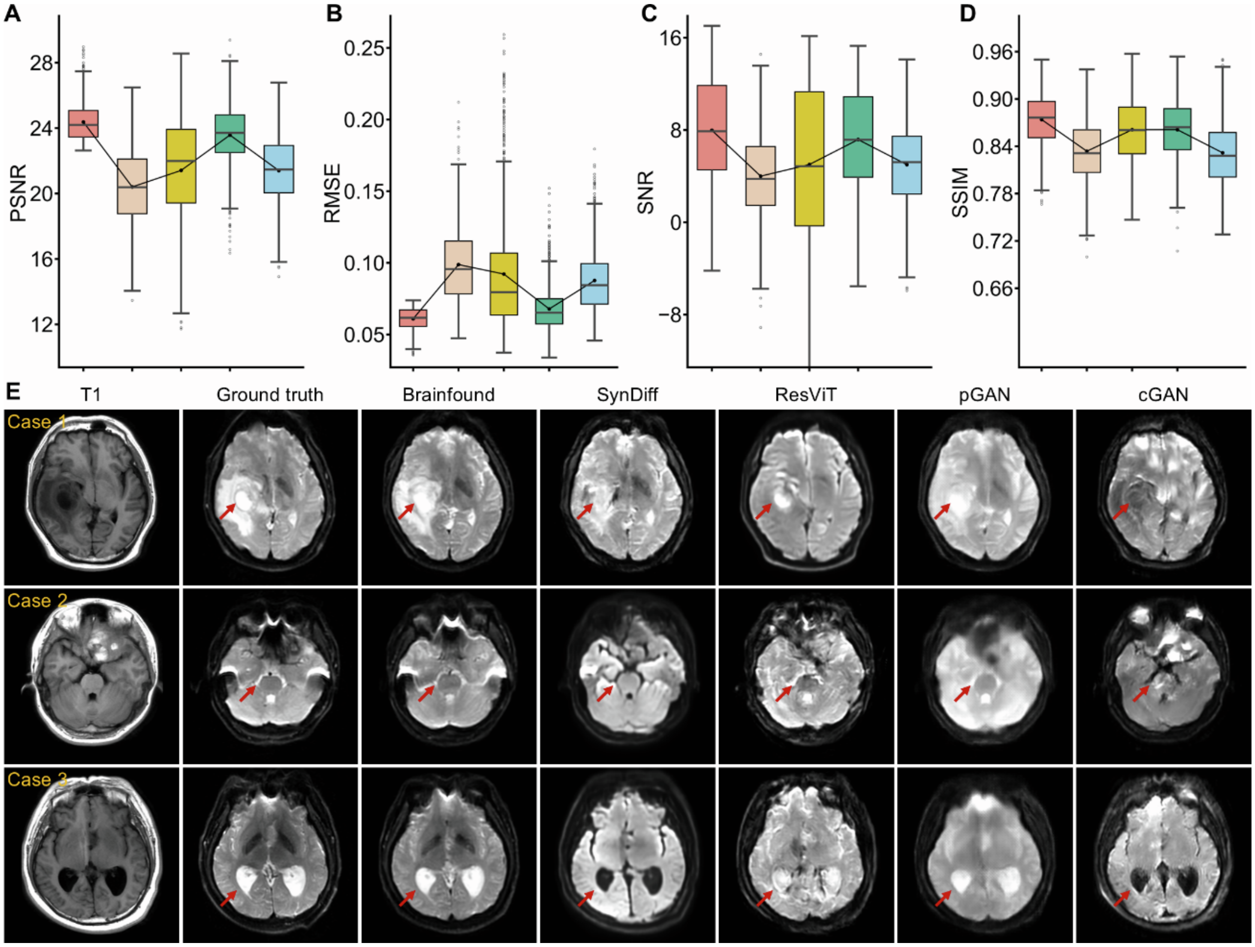

Fig. S24. Performance of Brainfound for MRI modality translation from T1WI to low-b-value DWI

(A-D) Quantitative comparison was made of T1WI to low-b-value DWI image translation results using five methods: Brainfound, SynDiff, ResViT, pGAN, and cGAN (n=1936). PSNR, RMSE, SNR, and SSIM were calculated individually. Brainfound achieved the highest scores in all metrics.

(E) Three cases of T1WI to low-b-value DWI Image translation via five models. As shown by the red arrows, the vasogenic edema and cystic lesions (case 1) and the CSF in the lateral ventricles (case 3) were accurately transformed into the high signal from the low signal on the original T1WI by Brainfound. In case 2, Brainfound provides a conversion that is closest to the ground truth.

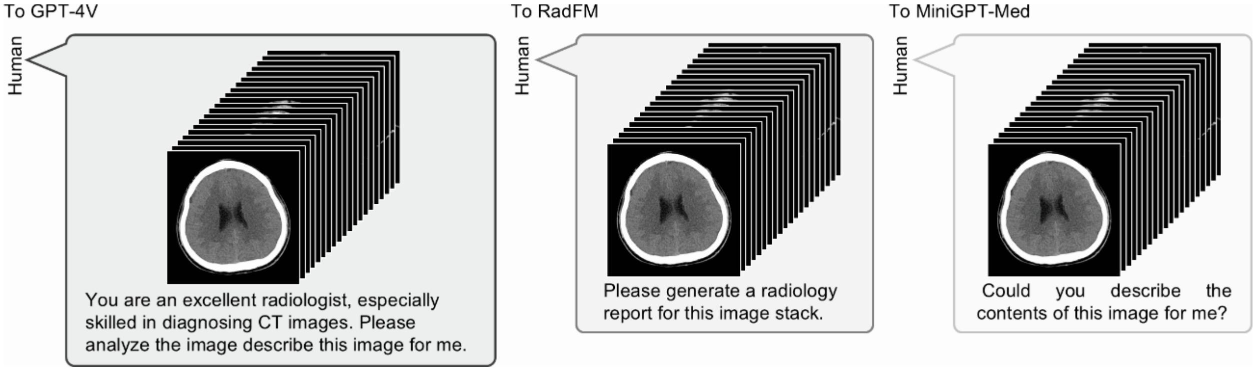

Fig. S25. Prompts used for radiology report generation

Columns from left to right show the prompts used for GPT 4V, RadFM, and MiniGPT-Med.

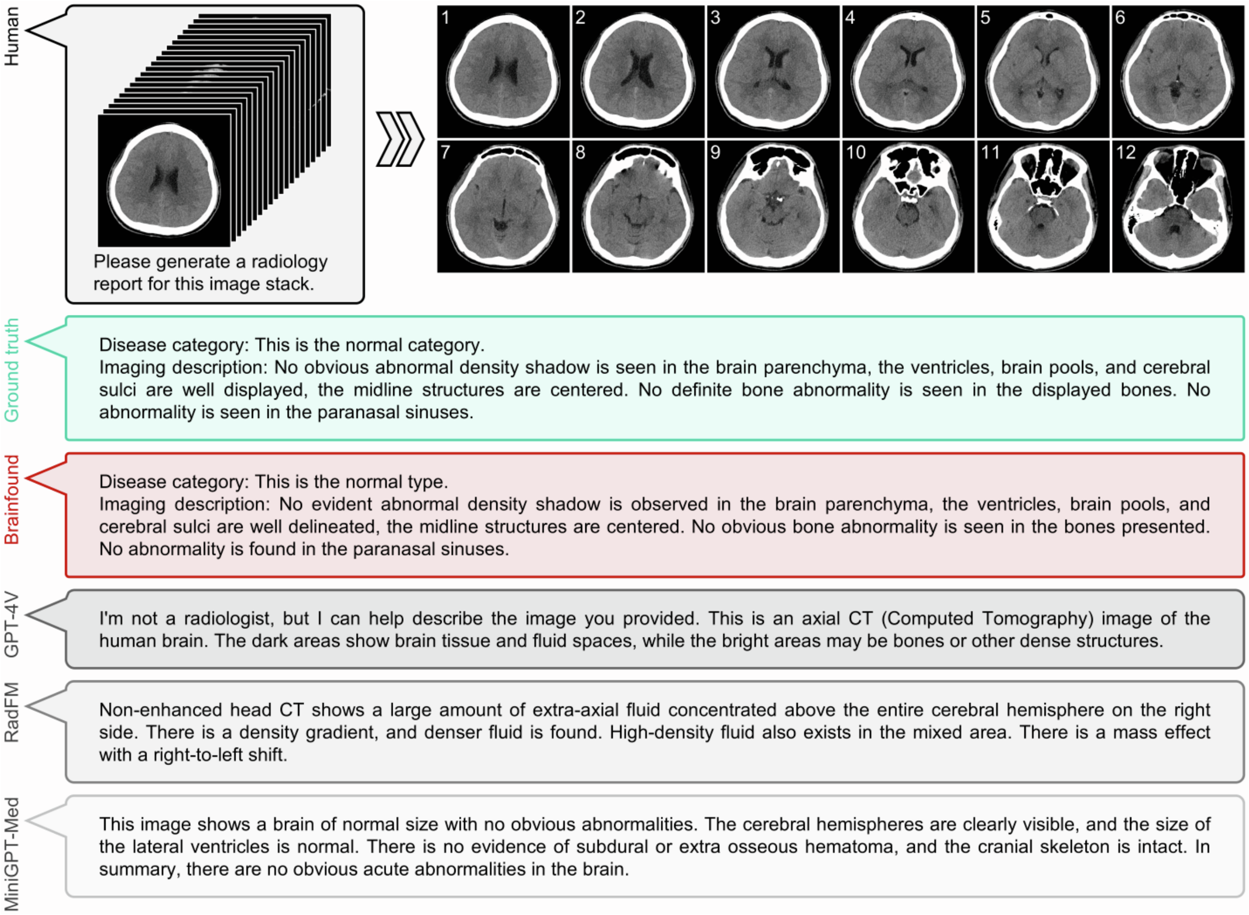

Fig. S26. Report generation example for a normal brain CT scan

The top panel shows the input CT scan and the prompt employed for report generation. A set of 12 representative slices from the same scan is shown on the right. The bottom panel presents the reference report written by an experienced doctor, together with reports generated by Brainfound, GPT4-V, RadFM, and MiniGPT-Med. Differences across generated reports are illustrated in terms of clinical formatting, terminology, and internal consistency.

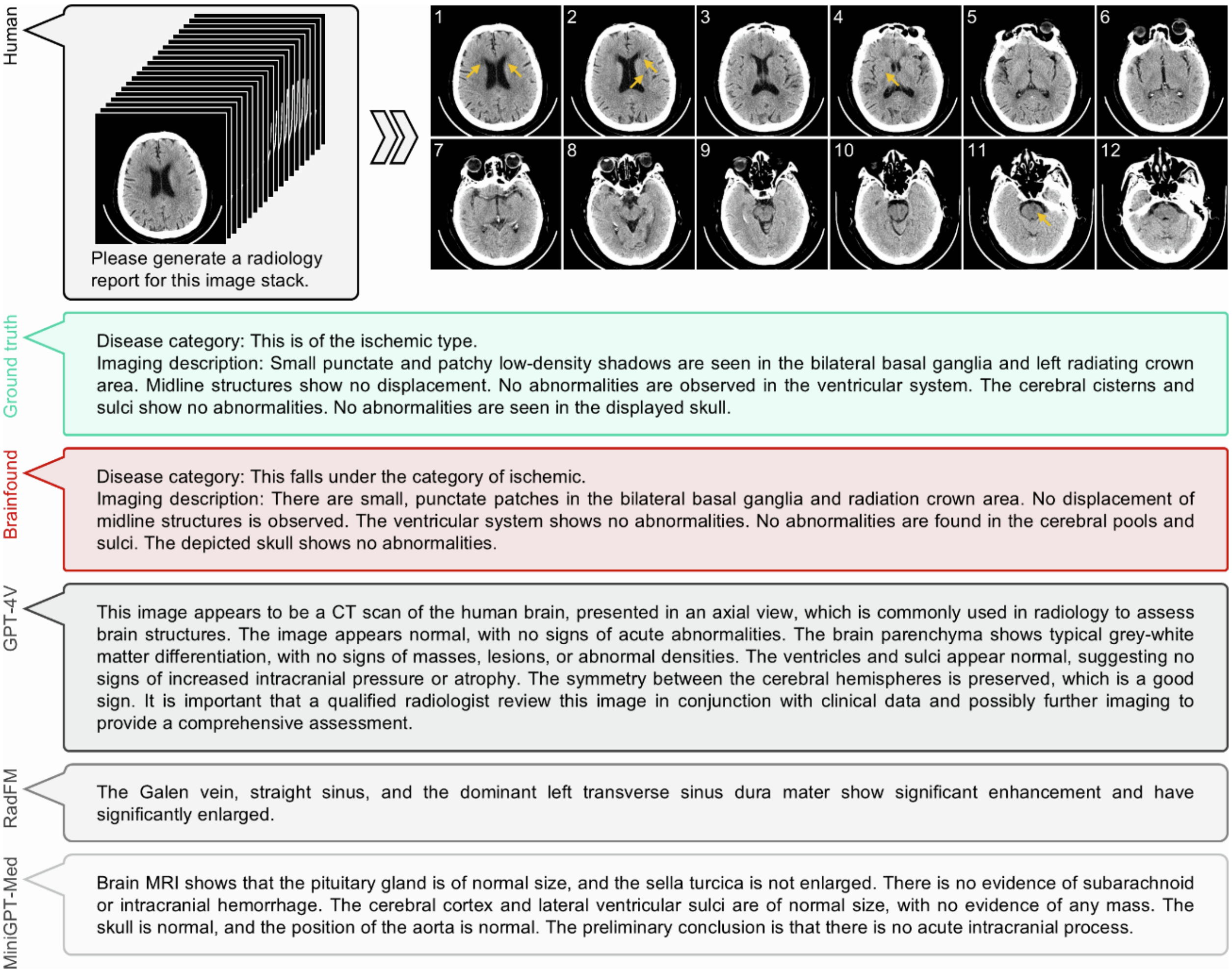

Fig. S27. Report generation example for an ischemic brain CT scan

The top panel shows the input CT scan and the prompt used in the examination. On the right, there are 12 typical brain CT images from the scan. Subsequently, reports crafted by an experienced doctor, along with those formulated by Brainfound, GPT4-V, RadFM, and MiniGPT-Med, are displayed. In this example, the RadFM output provides limited coverage of key imaging findings, and the MiniGPT Med output incorrectly refers to the input as an MRI sequence.

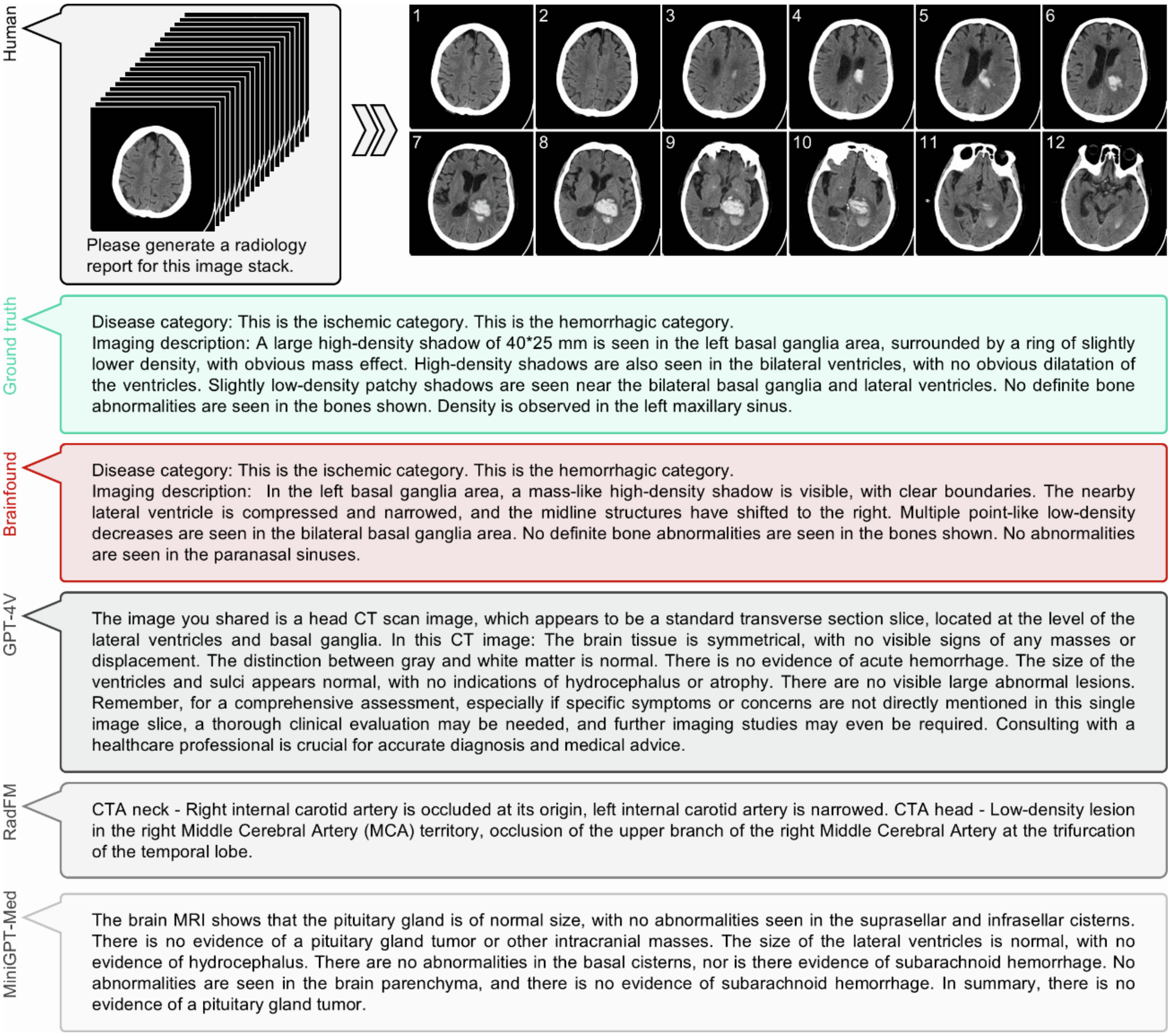

Fig. S28. Report generation example for a hemorrhagic brain CT scan

The upper portion of the display illustrates the input CT scan and the prompt used in the examination. On the right, there are 12 typical CT images from the scan. Subsequently, reports crafted by an experienced doctor, along with those formulated by Brainfound, GPT4-V, RadFM, and MiniGPT-Med, are showcased. In this example, the GPT 4V output does not explicitly list the diagnoses described in the reference report. The RadFM output refers to the input as a CTA study, and the MiniGPT Med output refers to the input as an MRI sequence.

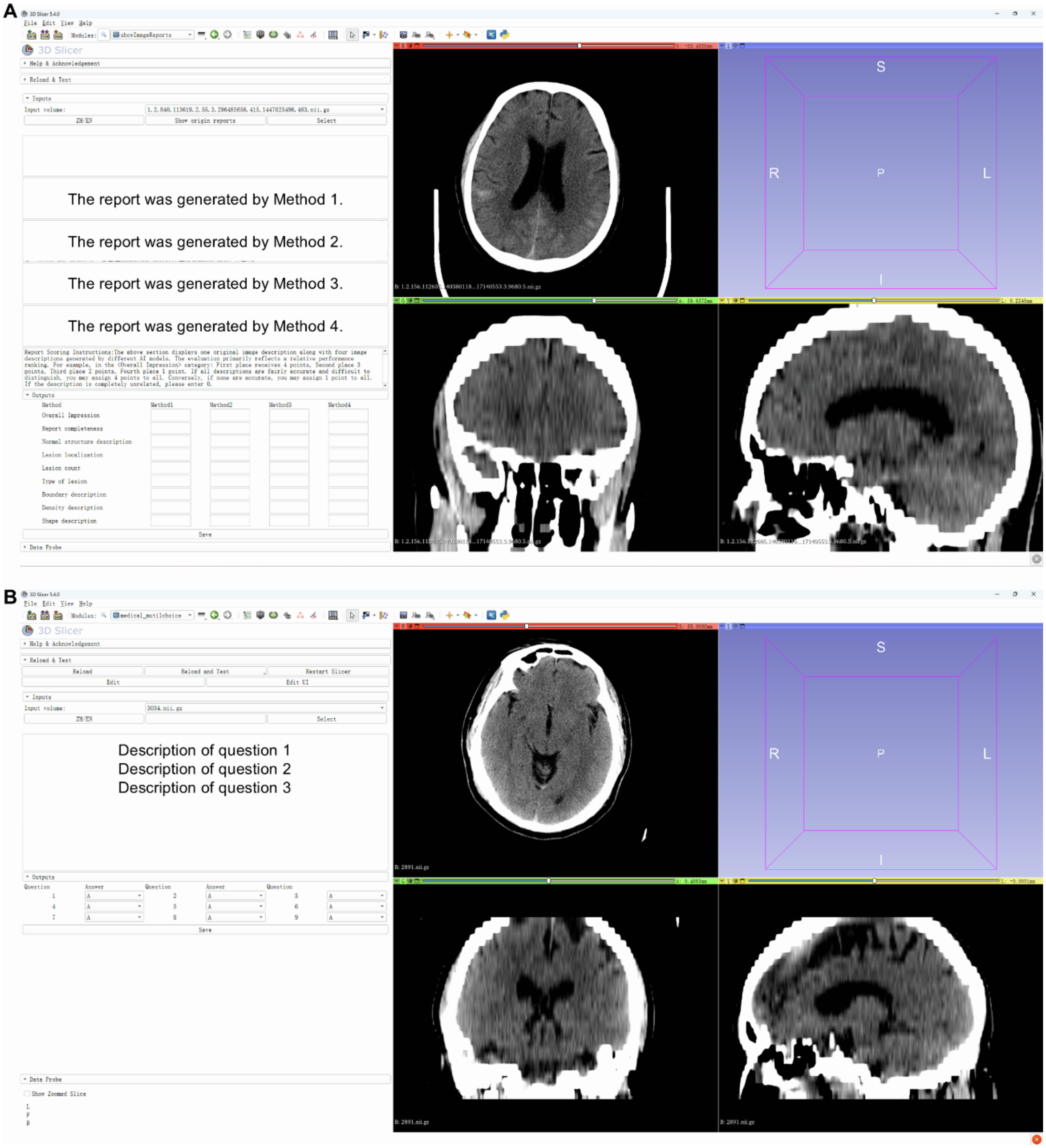

Fig. S29. The report evaluation and MCQ answering interfaces implemented in 3D Slicer

(A) Screenshot of the report evaluation interface. The CT scan associated with the current report is visualized in a three dimensional view, and reports generated by four methods are displayed for expert review together with a reference report written by an experienced radiologist. Report quality is rated across nine evaluation dimensions.

(B) Screenshot of the multiple choice question answering interface. This panel delineates three MCQs, each with multiple answer choices, where doctors are prompted to input their selections at the designated lower segment of the interface. For more details, refer to https://github.com/gingerbread000/SlicerMedicalReportGrading.

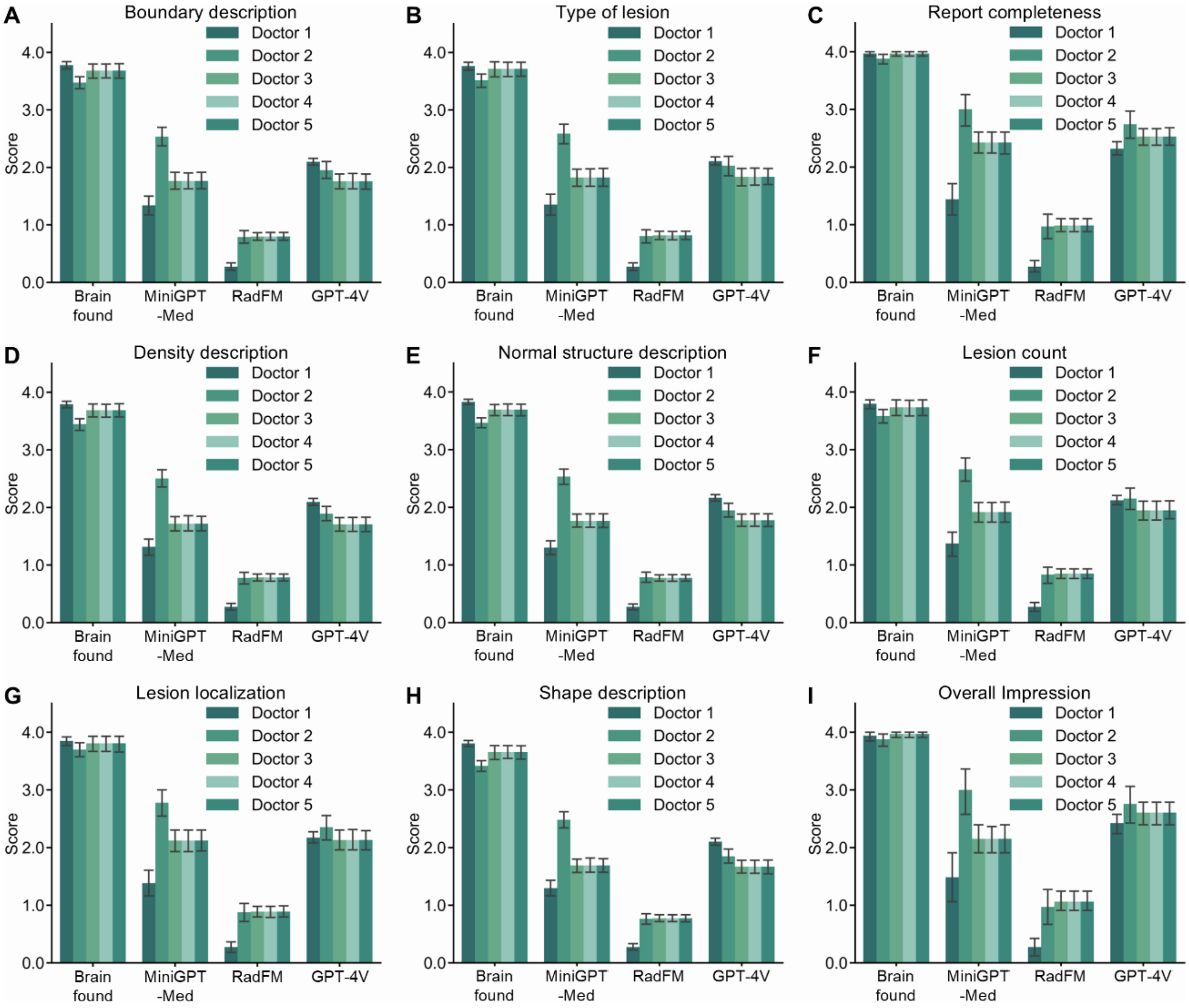

Fig. S30. Expert scoring of reports generated by four methods across nine evaluation dimensions

(A) Boundary description.
(B) Lesion type.
(C) Report completeness.
(D) Density description.
(E) Description of normal structures.
(F) Lesion count.
(G) Lesion localization.
(H) Shape description.
(I) Overall impression. Scores were provided by five experienced clinicians with a mean of 6.4 years of practice.

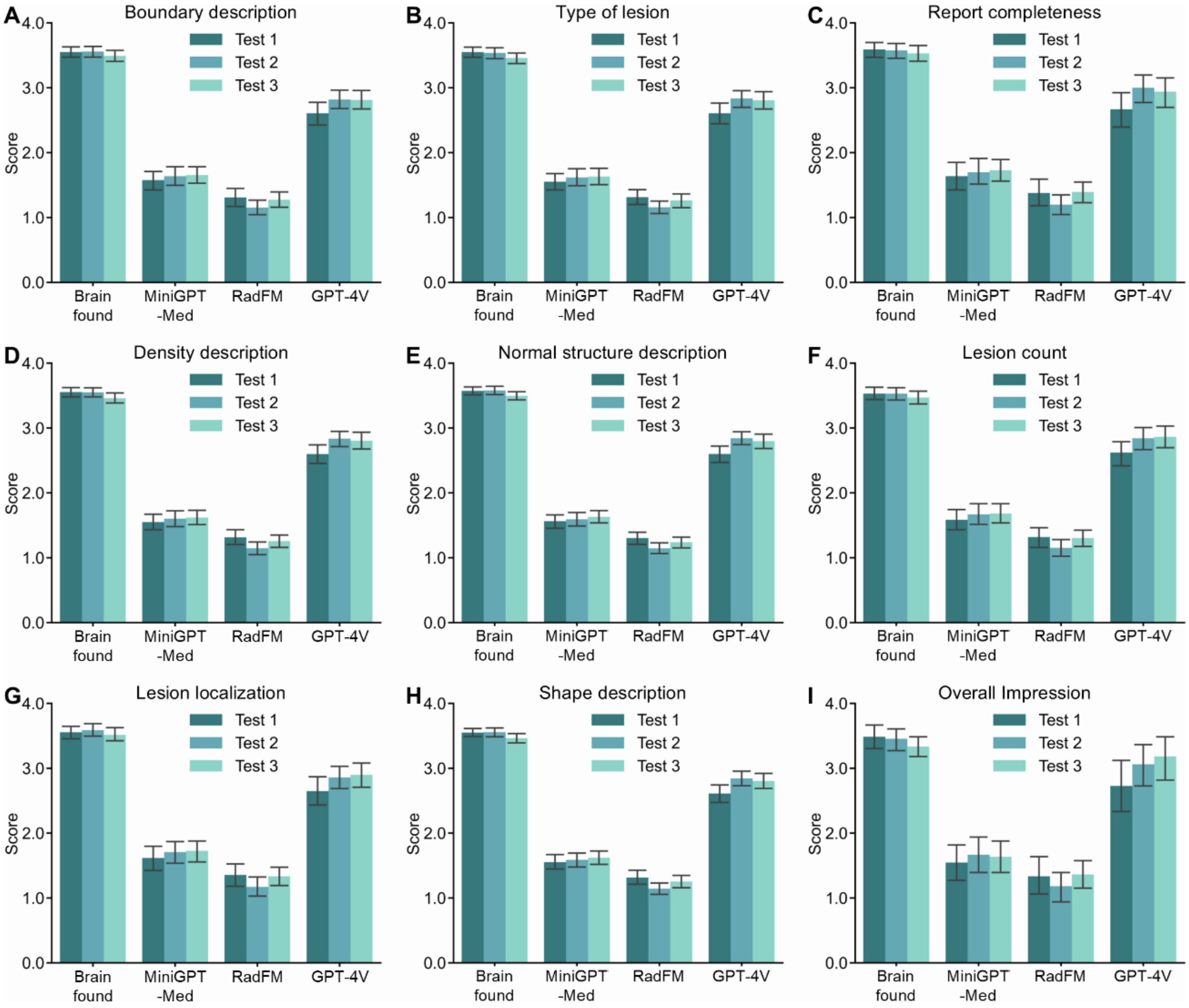

Fig. S31. GPT-4 based scoring of reports generated by four methods across nine evaluation dimensions

(A) Boundary description.

(B) Lesion type.

(C) Report completeness.

(D) Density description.

(E) Description of normal structures.

(F) Lesion count.

(G) Lesion localization.

(H) Shape description.

(I) Overall impression. The prompt for GPT-4 during the evaluation process is as follows: *You are an excellent radiologist, particularly skilled in determining whether a brain CT report is correct and compliant with standards. I will provide you with 5 reports, the first of which is written by a professional doctor after interpreting the CT. The other four reports are written by four different methods. I need you to score the other four reports based on the first report. Please score separately for the following aspects: overall*

impression, report completeness, normal structure description, lesion localization, lesion quantity, lesion type boundary description, density description, and shape description.

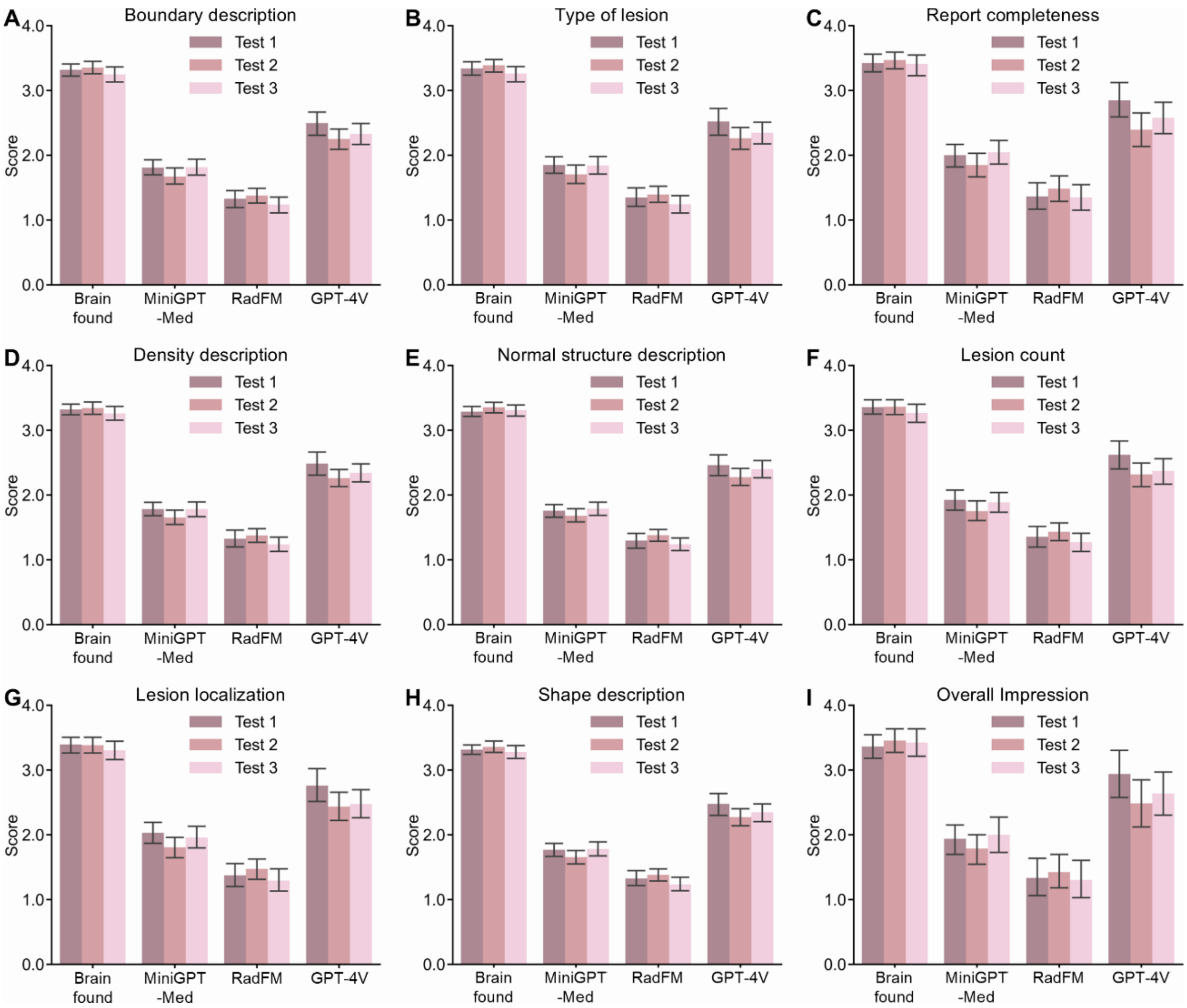

Fig. S32. GPT-4o based scoring of reports generated by four methods across nine evaluation dimensions

(A) Boundary description.

(B) Lesion type.

(C) Report completeness.

(D) Density description.

(E) Description of normal structures.

(F) Lesion count.

(G) Lesion localization.

(H) Shape description.

(I) Overall impression. The prompt for GPT-4o during the evaluation process is the same as GPT-4 in Supplementary Fig. 31.

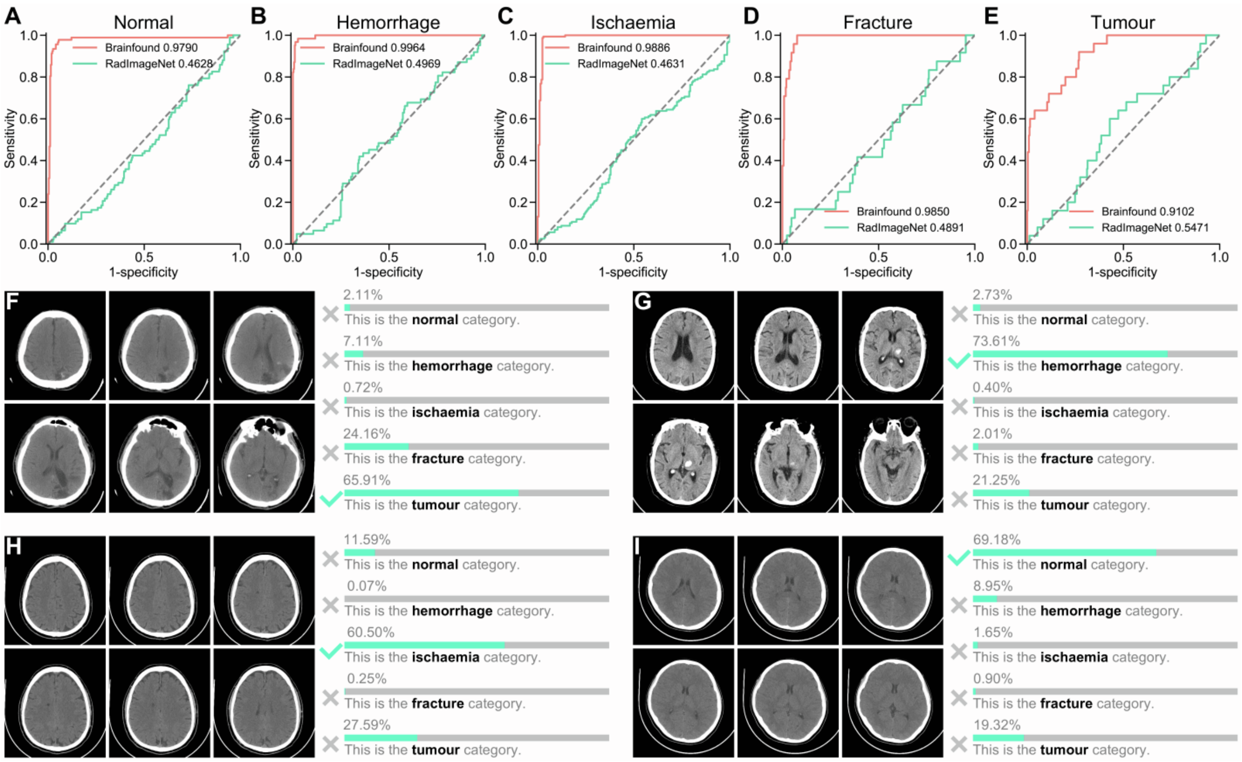

Fig. S33. Zero-shot classification performance on an external test set

(A-E) The zero-shot classification results of Brainfound, with RadImageNet serving as the comparison method on the external test set. The ROC curves, arranged from left to right, represent the categories of normal, hemorrhage, ischemia, fracture, and tumor.

(F) Predicted probability outputs for the tumor class.

(G) Predicted probability outputs for the hemorrhage class.

(H) Predicted probability outputs for the ischemia class.

(I) Predicted probability outputs for the normal class.

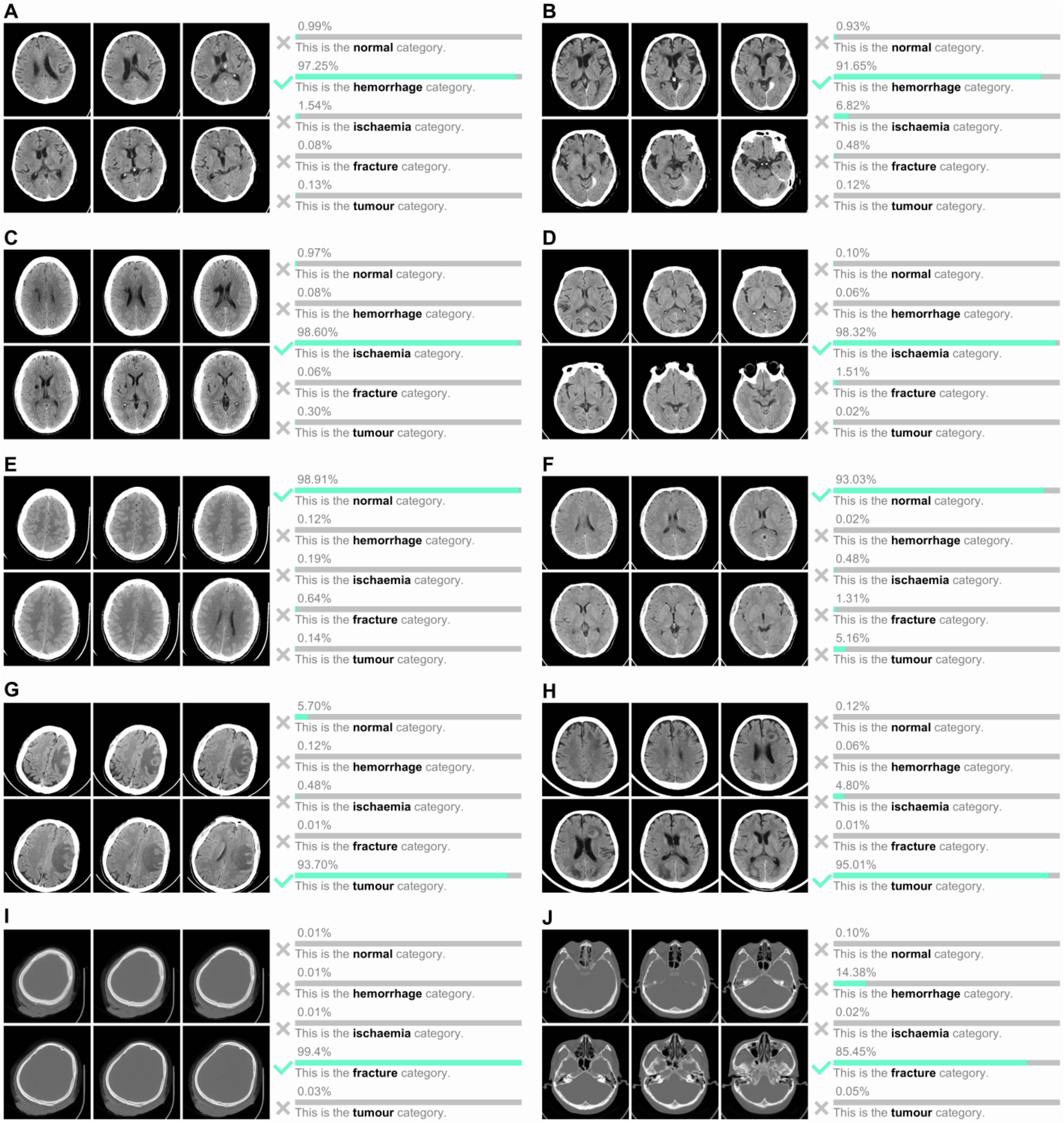

Fig. S34. Zero-shot classification examples using the aligned image encoder and text encoder in Brainfound

(A-B) Two cases for the classification of brain hemorrhage types. The output probabilities are 97.25% and 91.65%, respectively.
(B-D) Two cases for the classification of brain ischemia types. The output probabilities are 98.60% and 99.35%, respectively.
(E-F) Two cases for the classification of brain normal types. The output probabilities of 98.91% and 93.03%, respectively.
(F-H) Two cases for the classification of brain tumor types. The output probabilities are 93.70% and 95.01%, respectively.
(I-J) Two cases for the classification of brain fracture types. The output probabilities are 99.40% and 85.45%, respectively.

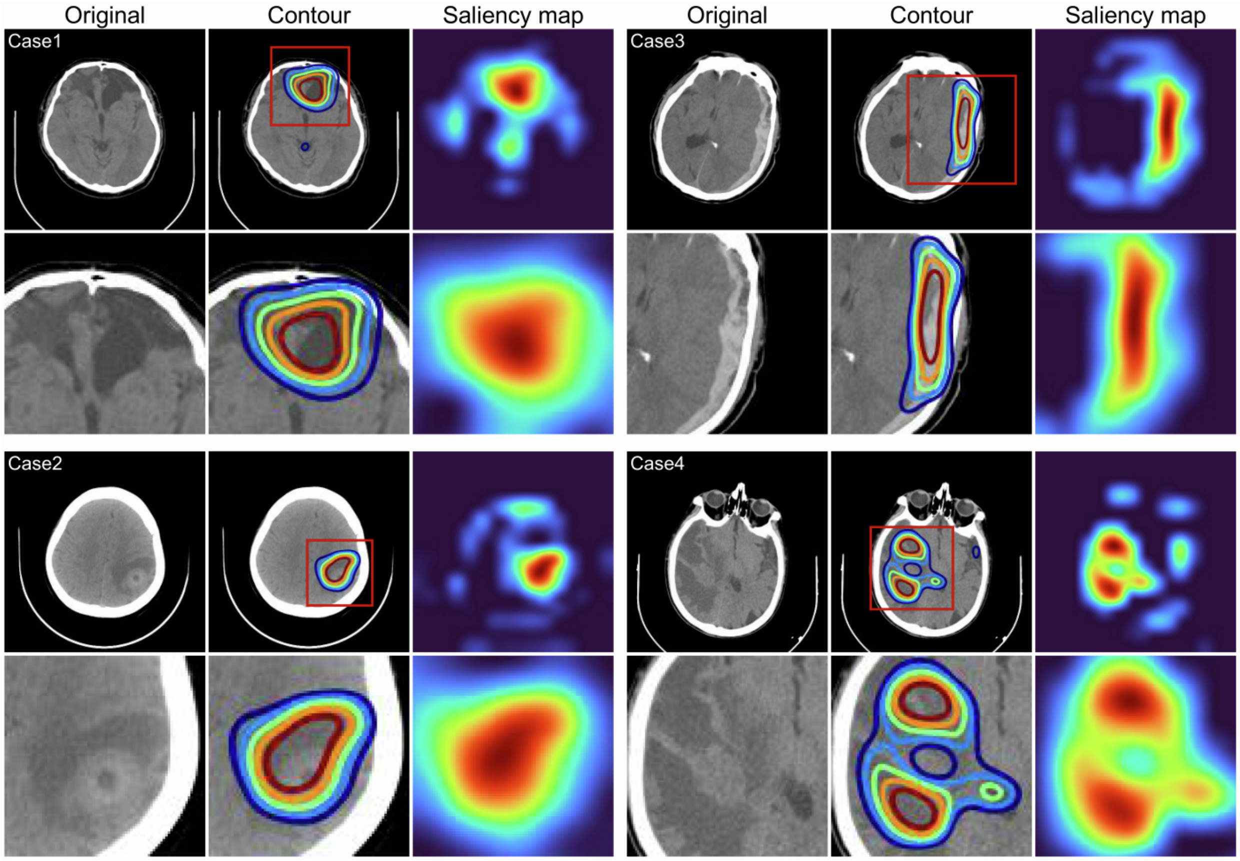

Fig. S35. Saliency maps generated by Brainfound for zero-shot classification

We showcased representative images of saliency maps for four brain CT images from Brainfound. The brain CT images are positioned in the first and fourth columns. The second and fifth columns show the saliency contours. The saliency maps developed by Brainfound are located in the third and sixth columns. The images in the second and fourth rows offer an enlarged perspective of the sections highlighted by red boxes in the first and third rows.

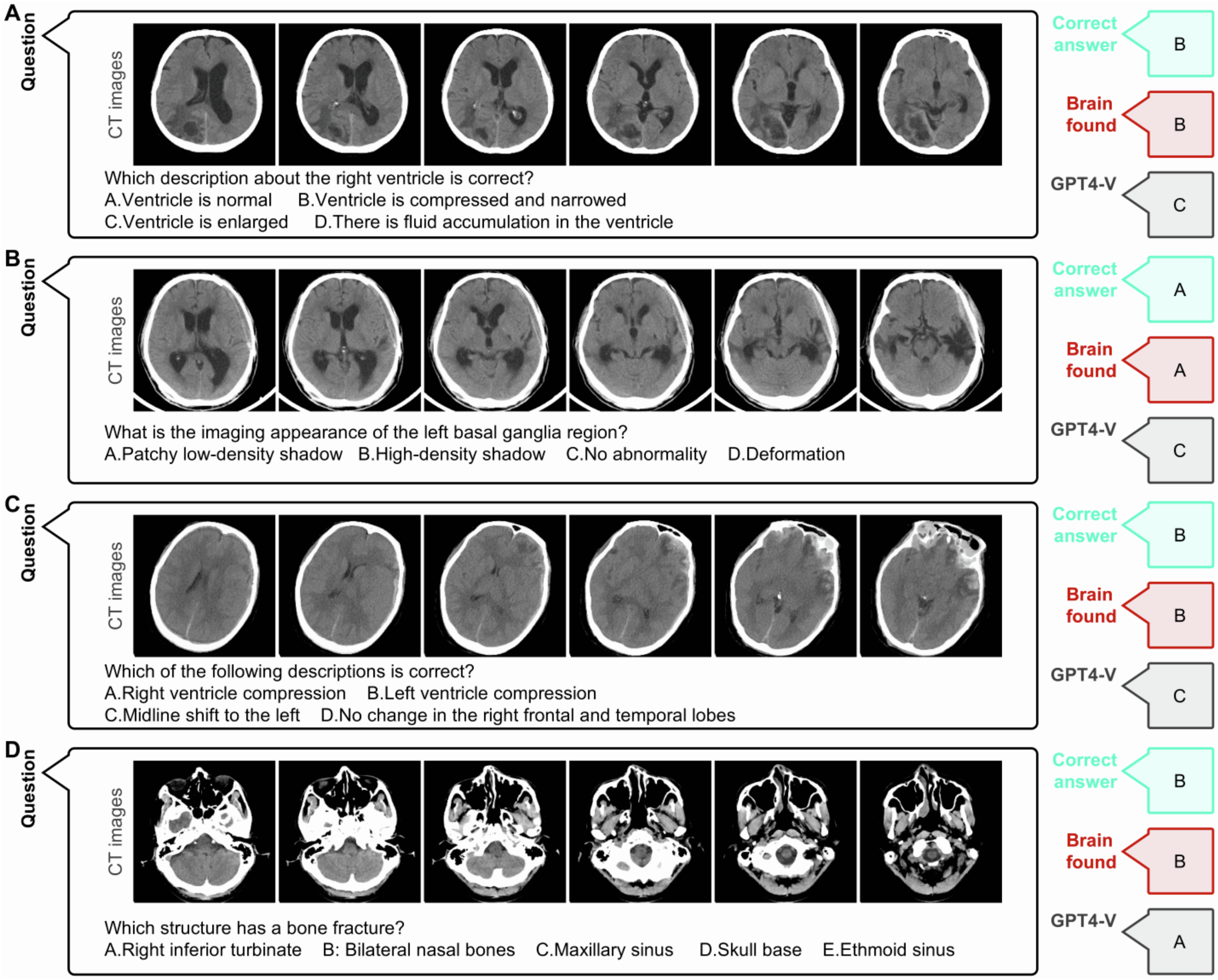

Fig. S36. The responses of Brainfound to multiple-choice questions on brain imaging, Part I

(A) With the CT images, Brainfound accurately identified the imaging characteristics of the left basal ganglia.
(B) Utilizing brain CT imaging, Brainfound determines which option is correct.
(C) Brainfound accurately selected the description of the right ventricle.
(D) Brainfound accurately identified the location of the fracture.

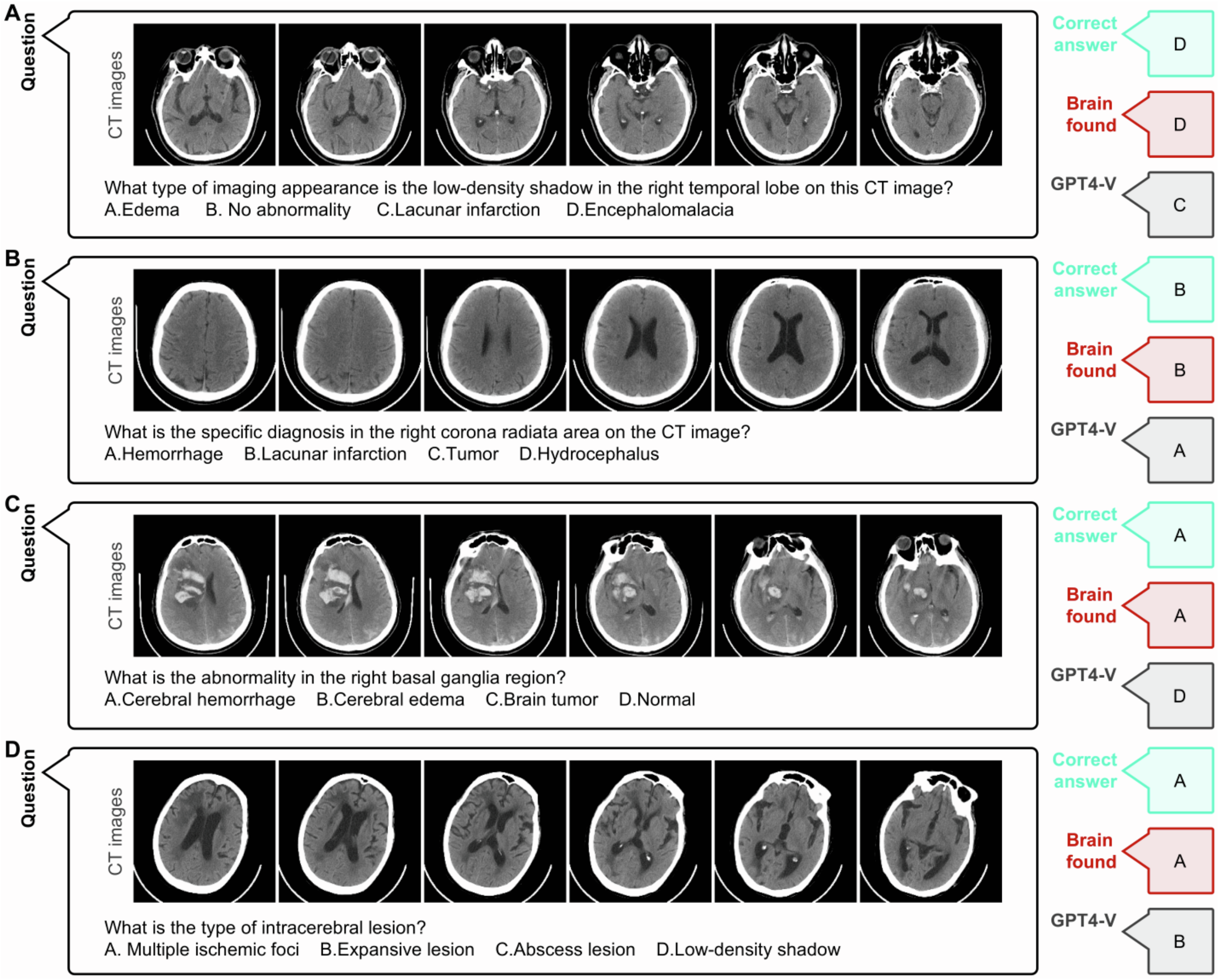

Fig. S37. The responses of Brainfound to multiple-choice questions on brain imaging, Part II

(A) Brainfound accurately determined the type of low-density shadow in the right temporal lobe depicted in the CT images.
(B) Brainfound accurately determined the specific diagnostic result for the right corona radiata area.
(C) Brainfound correctly identified the abnormality type in the right basal ganglia region.
(D) Brainfound accurately determined the type of brain lesion in the CT image.

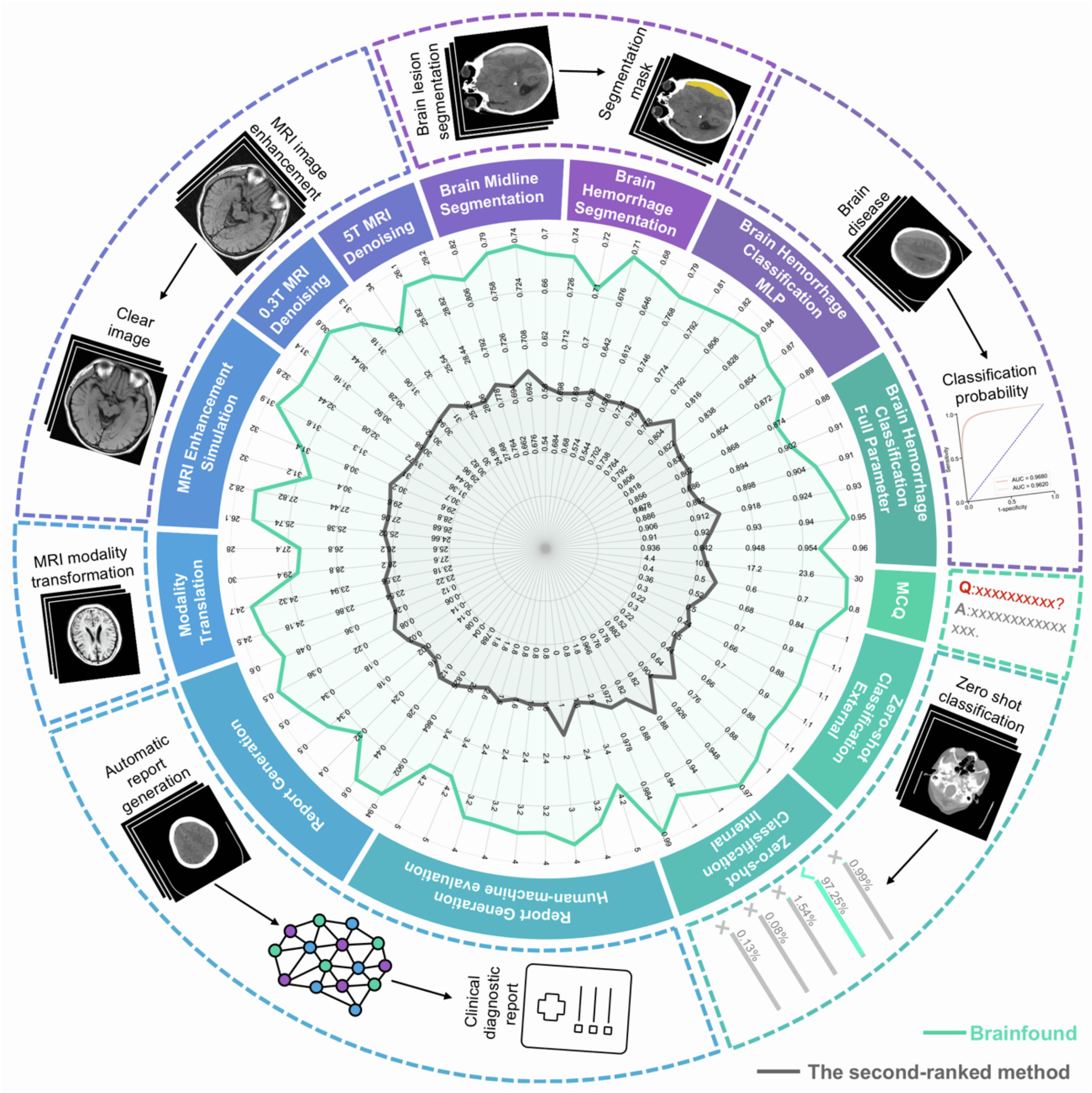

Fig. S38. Radar chart summarizing evaluation results across tasks

The radar chart displays all the experimental results. Two curves are plotted, one representing the results of Brainfound and the other representing the results of the second-ranked method in all comparisons. The names of the task types are labeled on the outside of the radar chart.

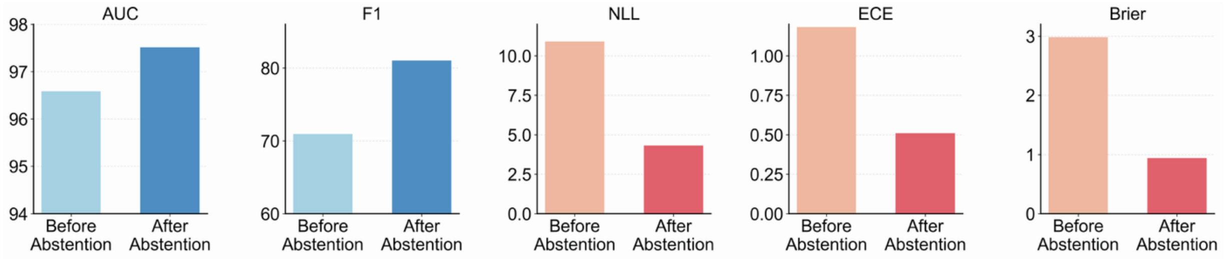

Fig. S39. Uncertainty-aware abstention improves both discrimination and calibration performance
The metrics in blue (AUC and F1) indicate better performance with higher values, whereas the metrics in red (NLL, ECE, and Brier score) indicate better performance with lower values.

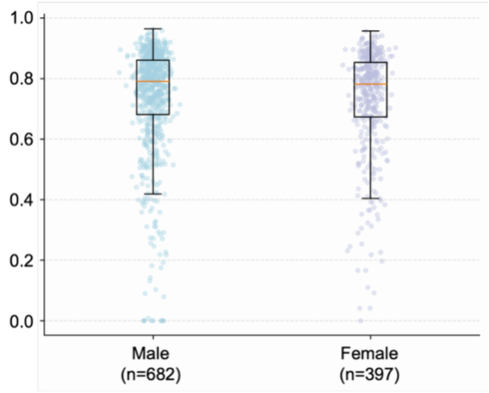

Fig. S40. Sex-based subgroup analysis of segmentation performance

Dice scores are highly comparable between male (n=682) and female (n=397) patients, with overlapping distributions and similar medians. No statistically significant difference was observed (two-sided Mann-Whitney U test, p = 0.297; negligible effect size).

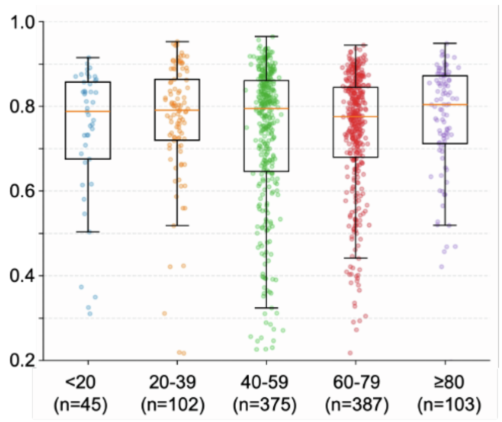

Fig. S41. Age-based subgroup analysis of segmentation performance.

Dice scores are stable across five age groups (<20 to ⩾80 years), with overlapping distributions and no monotonic age-related trend. No significant differences were observed (Kruskal–Wallis p = 0.323), and Dice showed no correlation with age, indicating no meaningful age-related performance bias.

Class	τ	Coverage (finetune set)	Coverage (non-abstained)
1	0.1873	0.9996	0.9995
2	0.0454	0.9448	0.9443
3	0.0201	0.9003	0.8994
4	0.1411	0.9957	0.9959
5	0.0322	0.7486	0.7471
6	0.0149	0.3415	0.3418

Table S1. The class-specific selection thresholds (τ) and the corresponding uncertainty coverage on the fine-tuning set and the non-abstained subset

Movie S1. The demo of Brainfound on free conversation around brain CT images

Two cases are presented: case 1 discusses what a cerebral infarction is and what a high-density linear signal shadow is. Case 2 is about cerebral hemorrhage, discussing what cerebral hemorrhage is and its effects on other areas
